# EvdS6 is a bifunctional decarboxylase from the everninomicin gene cluster

**DOI:** 10.1016/j.jbc.2023.104893

**Published:** 2023-06-05

**Authors:** Callie C. Dulin, Pankaj Sharma, Laura Frigo, Markus W. Voehler, T.M. Iverson, Brian O. Bachmann

**Affiliations:** 1 Department of Chemistry, Vanderbilt University, Nashville, Tennessee, USA; 2 Department of Pharmacology, Vanderbilt University, Nashville, Tennessee, USA; 3 Center for Structural Biology, Vanderbilt University, Nashville, Tennessee, USA; 4 Department of Biochemistry, Vanderbilt University, Nashville, Tennessee, USA

**Keywords:** decarboxylase, enzyme turnover, mutagenesis, natural product biosynthesis, nicotinamide adenine dinucleotide (NAD), oligosaccharide, protein structure, short-chain dehydrogenase/reductase (SDR), xylose synthase

## Abstract

The everninomicins are bacterially produced antibiotic octasaccharides characterized by the presence of two interglycosidic spirocyclic ortho-δ-lactone (orthoester) moieties. The terminating G- and H-ring sugars, L-lyxose and C-4 branched sugar β-D-eurekanate, are proposed to be biosynthetically derived from nucleotide diphosphate pentose sugar pyranosides; however, the identity of these precursors and their biosynthetic origin remain to be determined. Herein we identify a new glucuronic acid decarboxylase from *Micromonospora* belonging to the superfamily of short-chain dehydrogenase/reductase enzymes, EvdS6. Biochemical characterization demonstrated that EvdS6 is an NAD^+^-dependent bifunctional enzyme that produces a mixture of two products, differing in the sugar C-4 oxidation state. This product distribution is atypical for glucuronic acid decarboxylating enzymes, most of which favor production of the reduced sugar and a minority of which favor release of the oxidized product. Spectroscopic and stereochemical analysis of reaction products revealed that the first product released is the oxidatively produced 4-keto-D-xylose and the second product is the reduced D-xylose. X-ray crystallographic analysis of EvdS6 at 1.51 Å resolution with bound co-factor and TDP demonstrated that the overall geometry of the EvdS6 active site is conserved with other SDR enzymes and enabled studies probing structural determinants for the reductive half of the net neutral catalytic cycle. Critical active site threonine and aspartate residues were unambiguously identified as essential in the reductive step of the reaction and resulted in enzyme variants producing almost exclusively the keto sugar. This work defines potential precursors for the G-ring L-lyxose and resolves likely origins of the H-ring β-D-eurekanate sugar precursor.

The everninomicin family of oligosaccharide natural products contains ribosome-targeting antibiotics produced by variants of the actinobacterial taxa *Micromonospora carbonacea* (1, 2, 3). One family member, everninomicin A (EVA) (Fig. 1*A*), was developed well into phase III clinical trials because of its potency against multidrug-resistant Gram-positive bacteria, including multidrug-resistant *Staphylococcus aureus* and vancomycin-resistant *Enterococci* (4, 5, 6, 7, 8). While these clinical trials were eventually discontinued, the remarkable efficacy of EVA against clinically relevant drug-resistant pathogens has prompted ongoing research into this molecule and suggests that everninomicin analogs may be viable broad-spectrum antibiotics.

**Figure 1:**
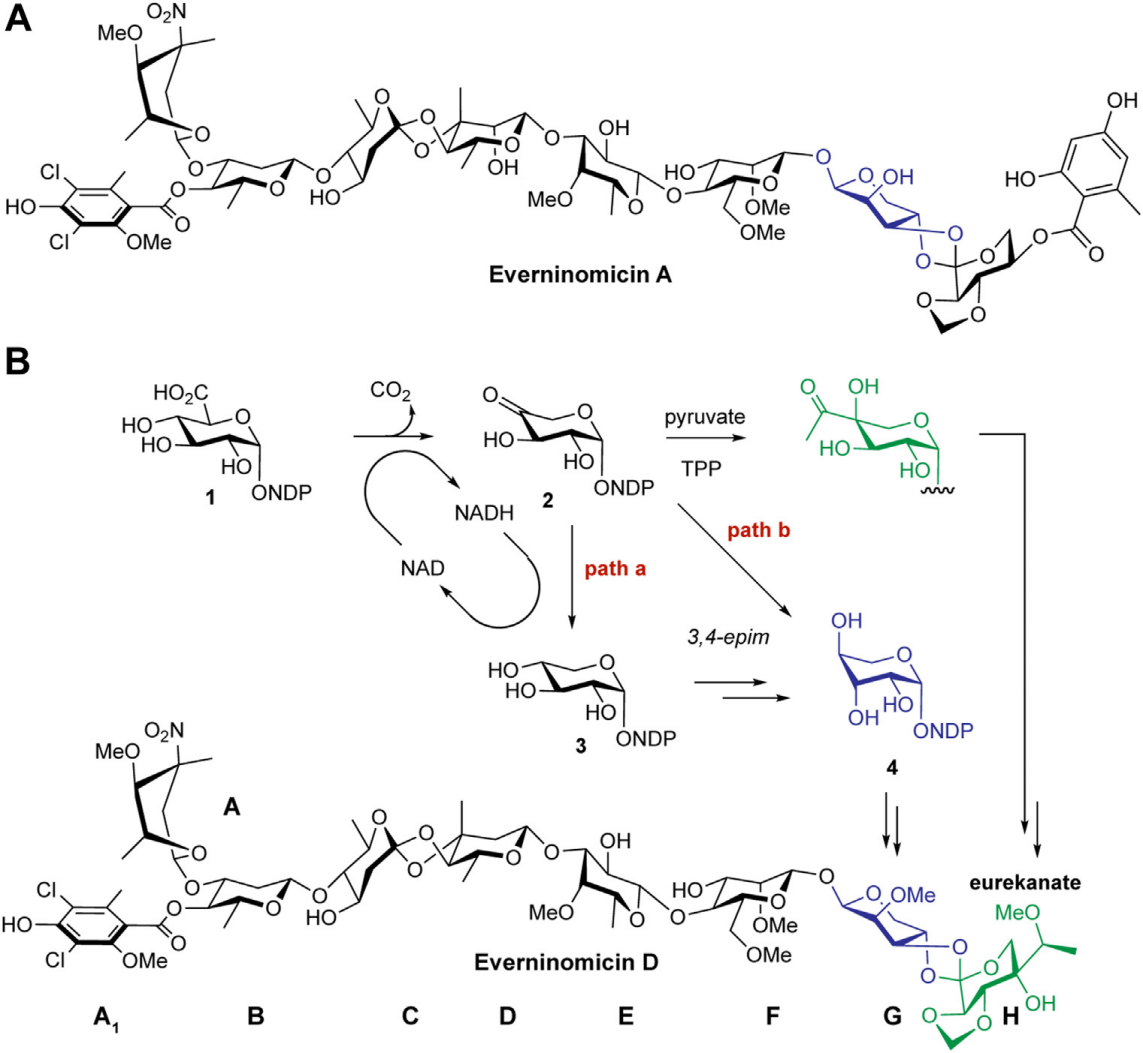
**Everninomicin A and D.***A*, the structure of everninomicin A, the original clinical candidate, rendered to approximate the conformation adopted in the ribosome-bound co-crystal structure. *B*, the structure of everninomicin D, showing the potential biosynthesis to both the G- and H-ring sugars. Path a follows other known NDP-D-glucuronic acid (1) decarboxylases in the formation of NDP-D-xylose (3) with subsequent enzyme(s) catalyzing the epimerization reactions at C-3” and C-4”. Path b suggests that the decarboxylase could also function as an C-3” epimerase and inverting C-4” reductase, leading directly to NDP-L-lyxose (4).

Recent insights into the mechanism of action and biosynthesis of everninomicins have outlined ways to potentially optimize ribosomal interactions, improve pharmacology and provide a means to modify the structure of the everninomicin scaffold. This includes the characterization of the target binding site of EVA and related oligosaccharides using cryogenic electron microscopy and X-ray crystallography (9, 10). Structures demonstrated that EVA interacts in a unique location of the 50S ribosomal subunit at a significant distance from the peptidyl-transferase center (PTC), where most ribosome-targeting antibiotics bind (9, 10). This is consistent with mechanistic studies using footprinting assays that showed EVA binds to hairpins 89 and 91 of the 23S rRNA (5, 11, 12). The potency and mechanism of the everninomicins, which may preclude cross-resistance with currently used PTC-targeting antibiotics, have prompted research into the biosynthesis of the family. Everninomicin biosynthetic studies have investigated the assembly of individual sugars, the extensive methylation pattern, and the formation of the orthoester rings (spirocyclic ortho-δ-lactones), which are essential pharmacophores in the everninomicins (13, 14, 15, 16, 17). Insights into the biosynthetic pathway for everninomicins provide a basis for generating analogs with improved pharmacological properties and at higher yields than the native strains.

EVA was withdrawn as a clinical candidate due to complications in generating a reproducibly safe formulation (18, 19). The cause of formulation-dependent toxicity was reported to be the formation of aggregates in formulary preparations. Formulations that contained increased aggregates, as confirmed by dynamic light scattering, were correlated with increased incidents of adverse reaction syndromes in mice (18). A potential mechanism of aggregation of EVA is *via* π-stacking interactions from flanking aromatic rings (Fig. 1*A*). Everninomicin D (EVD) is an equipotent analog of EVA, possessing a single dichloroisoeverninic acid aromatic ring, with the potential for decreased aggregation susceptibility (Fig. 1*B*) (4, 20). Instead of a second orsellinic acid moiety (A_2_-ring) as in EVA, the eastern hemisphere of EVD is terminated by a pentose sugar, L-lyxose (G-ring), linked *via* an orthoester moiety to another pentose-derived branched-chain heptose sugar, eurekanate (H-ring) (Fig. 1*B*, blue and green respectively). As EVD has demonstrated efficacy and low toxicity in multiple animal models, we propose it has significant potential for development as a lead antibiotic compound (4). Correspondingly, we have targeted understanding the distinguishing features of the biosynthesis of EVD, encoded by a 50-gene biosynthetic gene cluster (BGC) in *M. carbonacea* var. *aurantiaca*, in order to enable its further development as a clinical candidate (Fig. 2*B*) (13, 14, 16).

**Figure 2:**
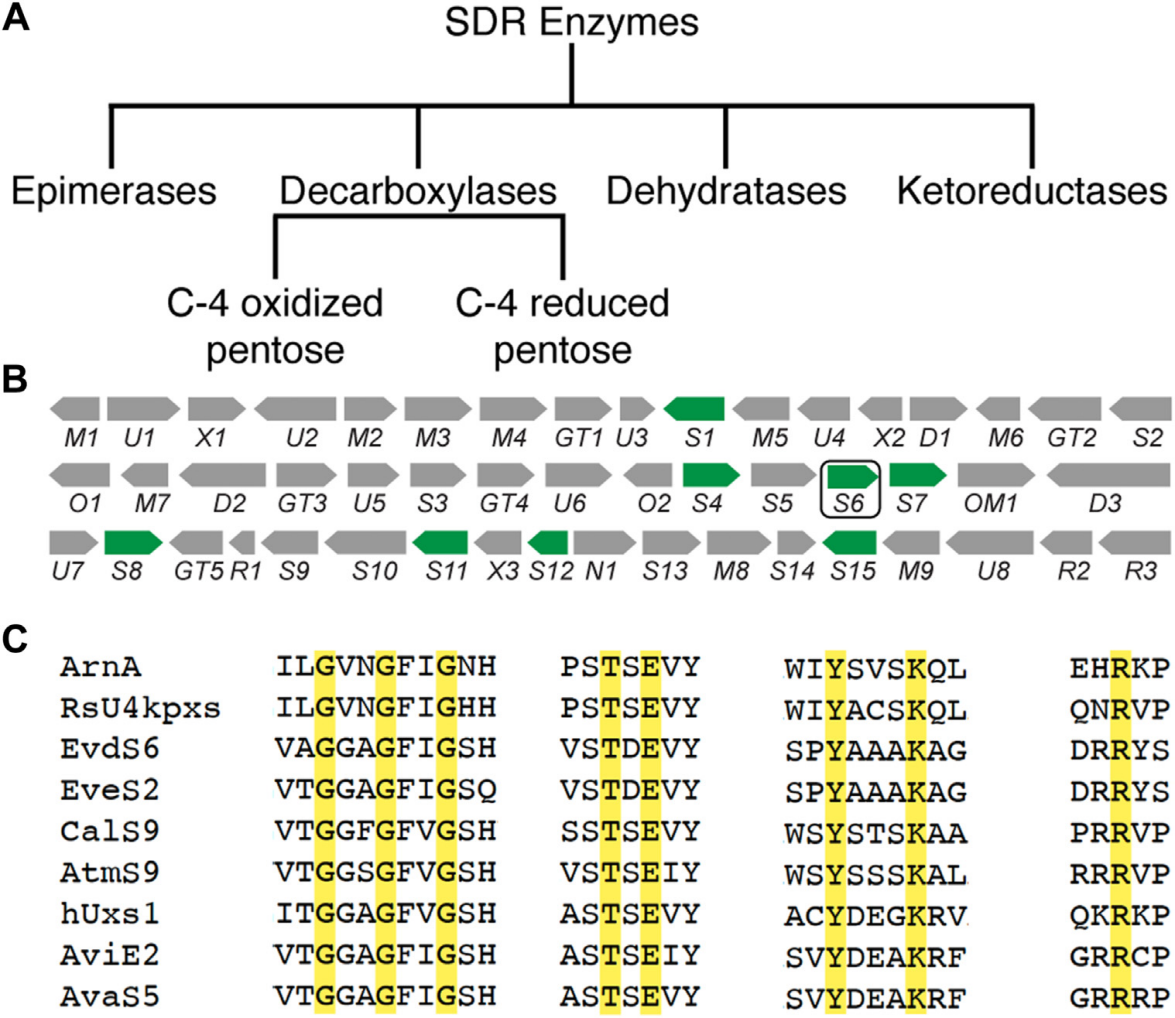
**SDR enzymes and the *evd* gene cluster.***A*, the different types of sugar-modifying SDR enzymes are highlighted. Decarboxylase enzymes can be further divided into those that produce a C-4” oxidized product and those that produce a C-4” reduced product, NDP-D-xylose. *B*, the *evd* biosynthetic gene cluster of *Micromonospora carbonacea* var. *aurantiaca*. The putative SDR enzymes are highlighted in *green*, with EvdS6 circled. *C*, specific sections of the decarboxylase enzyme sequence alignments that facilitated the identification of the *evd* decarboxylase, EvdS6. A complete alignment is shown in Fig. S1.

Determining the biosynthesis of the two terminating pentose sugars is integral to understanding the biosynthesis of EVD. Most reported pyranose pentose sugars are derived *via* glucuronic acid decarboxylases, which are short-chain dehydrogenase/reductase (SDR) enzymes that oxidize the C-4” of NDP-D-glucuronic acid (NDP-GlcA, **1**) to a ketone using NAD^+^, facilitating C-6” decarboxylation, with subsequent reduction at C-4” to generate NDP-D-xylose (**3**). The branched-chain sugar eurekanate is likely derived *via* a thiamine pyrophosphate (TPP)-dependent acetyl transfer to the glucuronic acid decarboxylase intermediate, a 4-keto-pentose product (**2**), in analogy to the biosynthesis of yersiniose A by the acetolactate synthase-type enzyme, YerE (21). The biosynthetic origin of the G-ring precursor (NDP-L-lyxose, **4**) in *Micromonospora* is unknown at this time; however, it can in principle be derived *via* one of two general pathways (Fig. 1*B*). L-lyxose could be biosynthesized by subsequent enzymes through C-3”/4” epimerization of NDP-D-xylose (**3**), the product of previously characterized glucuronic acid decarboxylases (Fig. 1*B*, path a). Alternatively, L-lyxose could be biosynthesized by the decarboxylase alone through C-3” epimerization of NDP-4”-keto-D-xylose (**2**) prior to a stereochemically-inverting reduction at C-4” (Fig. 1*B*, path b). With either pathway, the biosynthetic origins of the G- and H-rings could both depend upon the same decarboxylase enzyme.

To understand the biosynthesis of the G- and H-rings of everninomicin D, in this work we identified and characterized EvdS6, a novel glucuronic acid decarboxylase that was identified from among the eight SDR enzymes within the everninomicin D gene cluster (*evd*). Biochemical characterization of EvdS6 and stereochemical determination of its reaction products demonstrates that it produces a mixture of two products differing in the oxidation state at the C-4” position: UDP-D-xylose and UDP-4”-keto-D-xylose. These two sugars potentially define the precursors for the generation of the G- and H-rings, respectively. X-ray crystallographic studies showed that, despite significant sequence variation, the active site of EvdS6 closely aligns with other SDR enzymes, including two other decarboxylase enzymes. Mutagenesis studies were informed by the structure and identified residues essential for the reduction of half of the catalytic cycle. Among the mutants were biocatalysts capable of producing exclusively the oxidized product. EvdS6, as a bifunctional enzyme, produces the biosynthetic precursor to the H-ring of EVD and potentially also the G-ring, L-lyxose.

## Results

### SDR sequence motifs facilitate the identification of the decarboxylase candidate within the *evd* biosynthetic gene cluster

Glucuronic acid decarboxylases, or xylose-synthases as they are also known, belong to the superfamily of short-chain dehydrogenase/reductase (SDR) enzymes, one of the largest protein superfamilies, which is present across all kingdoms of life (22, 23, 24, 25). SDR enzymes catalyze alcohol redox reactions on a variety of different substrates including sugars, steroids, and prostaglandins. SDR enzymes are characterized by low sequence identity (15–30 %), even within the subclasses But with high structural similarity across the superfamily (22, 24). In the *evd* biosynthetic gene cluster, identifying the glucuronic acid decarboxylase was difficult due to the limited distinguishing sequence characteristics between the subclasses of SDR enzymes. Of the 50 genes in the cluster, 15 of them were previously annotated as involved in sugar biosynthesis. To identify a glucuronic acid decarboxylase, BLAST sequence alignment was used. This identified that ten of the sugar-modifying genes are generic dehydratases or epimerases. A distinguishing feature of this superfamily of enzymes is the catalytic dyad YxxxK, which is proposed to participate in the NAD^+^-dependent oxidation common to SDR enzymes (23). Of the ten dehydratases or epimerases in the *evd* BGC, eight of them belong to the SDR superfamily. All SDR enzymes also contain a glycine-rich motif, typically GxxGxxG, or a variation thereof, that highlights a Rossmann fold for cofactor binding (24). In addition to the tyrosine and lysine, most SDR enzymes have either a serine or a threonine in the active site forming a catalytic triad: [S/T]x_n_YxxxK (23). Sequence analysis identified the eight SDR superfamily genes in the *evd* gene cluster all contain the expected YxxxK motif that hallmarks the SDR superfamily as well as the GxxGxxG motif that identifies the NAD^+^ cofactor binding site. The SDR enzyme superfamily is often divided into subtypes including classical, extended, divergent, and other smaller subtypes (25, 26). Those enzymes that transform NDP-activated sugars are often of the extended SDR subtype, which includes epimerases, dehydratases, ketoreductases, and decarboxylases (Fig. 2*A*) (27). Distinguishing between enzyme subclasses of this superfamily, even within a subtype, can be challenging based on this common catalytic triad and limited sequence identity beyond that. This is further complicated by the fact that some SDR enzymes have been characterized to perform multiple redox transformations in one enzyme active site. This is exemplified by FlaA1 of *Helicobacter pylori*, which has been characterized as a five-inverting 4,6 dehydratase, meaning it epimerizes the C-5 position upon NADH reduction after dehydration across C-4 and C-6 (28). Mannose 3,5 epimerases also perform multiple transformations in a single enzyme active site (29). Overall, this complicates identifying an SDR enzyme subclass based on sequence alone.

The conversion of UDP-glucuronic acid to UDP-xylose by a glucuronic acid decarboxylase was first confirmed in the fungus *Cryptococcus laurentii* (30). Since then this class of enzyme has also been characterized in a number of bacteria, including *Escherichia coli*, *Streptomyces* species, *Ralstonia solanacearum*, *Sinorhizobium meliloti*, *Bacteroides fragilis*, *Rhodothermus marinus*, and *Actinomadura melliaura*, and in eukaryotes including humans (31, 32, 33, 34, 35, 36, 37, 38, 39, 40). Glucuronic acid decarboxylases begin with NAD^+^-dependent oxidation at C-4” of NDP-GlcA, followed by decarboxylation of the C-6” carboxylate *via* an enolate intermediate, and then reduction of the C-4” position with NADH generated in the first phase of the reaction, thus regenerating the NAD^+^ cofactor (30). With this reaction scheme, most glucuronic acid decarboxylases produce NDP-D-xylose (**3**); however, a few enzymes, ArnA, AtmS9, CalS9, and RsU4pxs, have been characterized to release the oxidized product NDP-4”-keto-D-xylose (**2**) (32, 35, 39). In the investigation of ArnA, an *E. coli* glucuronic acid decarboxylase, R619 was identified as having significant sequence identity across the enzyme subclass and was proposed to be the catalytic base that facilitates the decarboxylation reaction (31). Subsequent structural and mutagenesis studies of the human glucuronic acid decarboxylase, hUXS1, do not define this arginine as catalytic (40). However, sequence identity to this strictly conserved arginine residue allowed for the identification of CalS9 and AviE2 as the glucuronic acid decarboxylases from the calicheamicin and avilamycin BGCs, respectively (33, 34). These analyses also identified that only one enzyme in the *evd* biosynthetic gene cluster, EvdS6, has all the necessary domains and an arginine residue that aligned with the conserved arginine residue identified in ArnA (Fig. 2*C*). Based on this sequence exploration, EvdS6 was investigated as a potential glucuronic acid decarboxylase in the biosynthesis of everninomicin D.

### EvdS6 is a bifunctional SDR enzyme capable of producing precursors for both the G- and H-rings of everninomicin D

**Figure 3:**
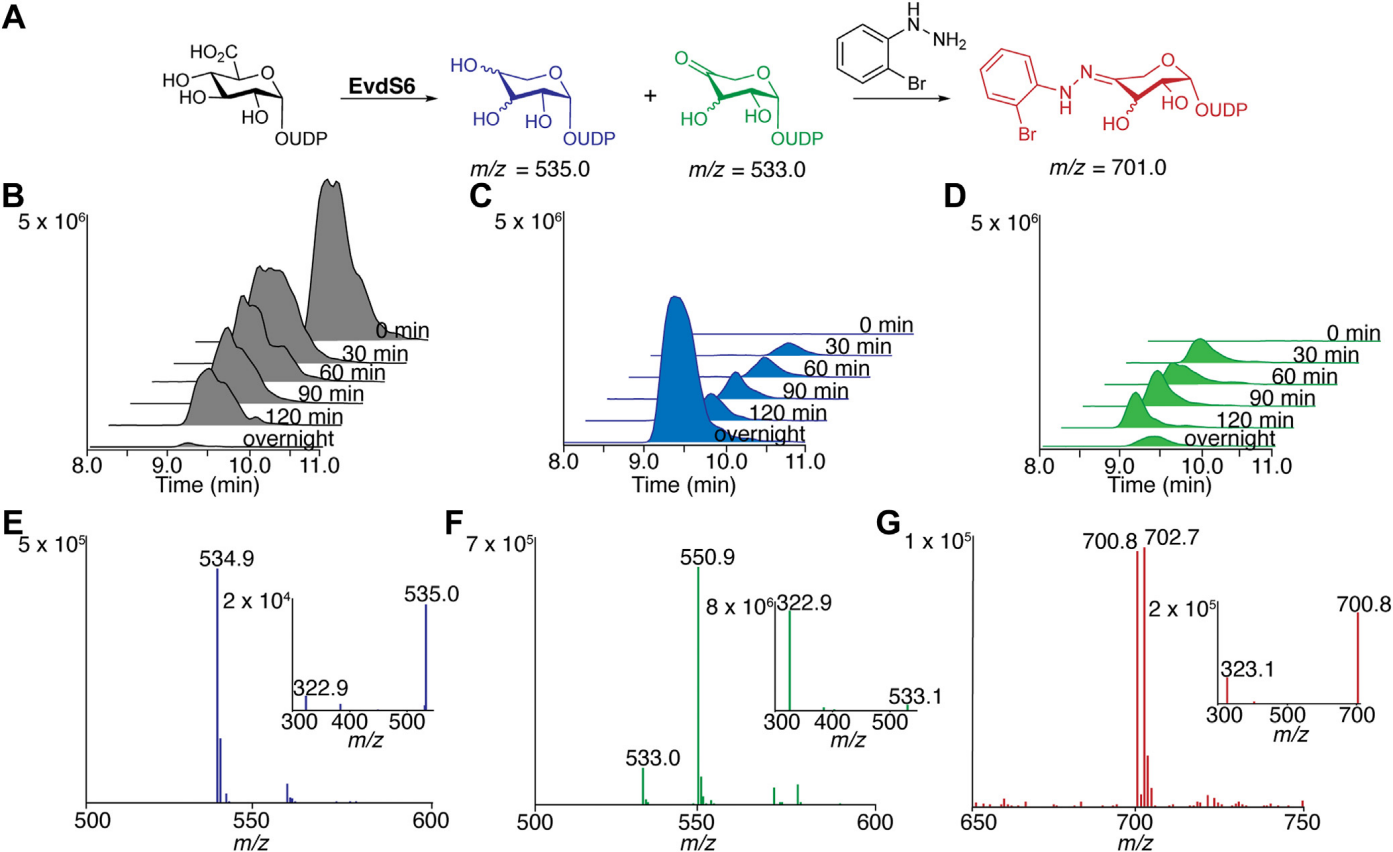
***In vitro* turnover of EvdS6.***A*, reaction scheme of EvdS6 turnover and the hydrazine addition to the 4-keto-pentose product. *B*, time course of the starting material, UDP-GlcA, EIC of m/z 579.0. *C*, time course of the reduced pentose product, EIC of m/z 535.0. *D*, time course of the oxidized 4-keto-pentose product, combined EICs of m/z 533.0 and 551.0. Time course reaction was set up with 1 mM UDP-GlcA, 0.1 mM NAD^+^, 50 mM HEPES pH 8.5, and 0.42 mg/ml EvdS6. *E*, MS and MS/MS (inset) of the reduced pentose product. *F*, MS and MS/MS (inset) of the oxidized 4-keto-pentose product. *G*, MS and MS/MS (inset) of the 2-bromophenyl hydrazone product.

To evaluate the function of EvdS6, the corresponding gene was cloned, and an N-terminal hexahistidine tag was added (Fig. S2). This gene was then heterologously expressed in *E. coli*. The proposed substrate of this decarboxylation enzyme is NDP-GlcA (**1**). The native enzyme could use either the uridine or thymidine diphosphate-activated glucuronic acid, but all studies herein used the UDP-GlcA as it was commercially available. HPLC/MS analysis of a time series incubation of purified EvdS6 with UDP-GlcA revealed two new compounds absent in the boiled enzyme control (Fig. 3, *B*–*D*). The turnover assays produced the chromatographically distinct compounds with mass-to-charge ratios (m/z) of 533.0 and 534.9 as monitored by HPLC-MS, using a graphitic matrix for separation (Fig. 3, *E* and *F*). Based on MS/MS fragmentation data, both new masses contain the UMP moiety (Figs. 3, *E* and *F*, insets and S3). EvdS6 appears to convert UDP-GlcA into a mixture of two different products that differ by 2 Da, which suggests the two products differ in an oxidation state. The m/z species of 533.0 and 534.9 are consistent with a UDP-4”-keto-pentose and a fully reduced UDP-pentose, respectively (Fig. 3*A*). The spectrum of the m/z 533.0 feature indicates a predominant m/z 550.9 peak, which correlates with the hydrated form of the UDP-4”-keto-pentose, known to be favored in solutions (Fig. 3*F*) (32). Upon extended incubation over 16 to 24 h, the ratio of products shifts from slightly favoring the m/z 533.0 product to a majority of the 534.9 product (Figs. 3*C* and S4). This provides additional evidence that the 533.0 m/z feature is a UDP-4”-keto-pentose, the canonical intermediate in the path to the m/z 534.9 product, a reduced UDP-pentose of unknown stereochemical configuration.

EvdS6 turnover was optimized by exploring buffer reagent, pH, cofactor, and reaction time. Incubation with NADP^+^ instead of NAD^+^, or without any exogenous cofactor at all, led to a fraction of the expected turnover, suggesting NAD^+^ as the cofactor (Fig. S4A). Turnover was screened in HEPES, Tris-HCl, and phosphate buffers, with no major differences noted between them. The observed turnover decreased significantly below pH 8.0, with no turnover observed below pH 7.0 (Fig. S4*B*). Salt additives were also screened, including NaCl, KCl, and MgCl_2_, with no apparent changes in reactivity. The reaction was followed by HPLC-MS for the first 2 h as well as overnight. In the first few hours, both products are produced in qualitatively similar amounts (Fig. S4*C*), slightly favoring the m/z 533.0 product; however, as stated earlier, with extended reaction timing, the reduced UDP-pentose (m/z 534.9) becomes the major product (Figs. 3, *C* and *D*, and S4*C*).

### Confirmation of the presence of the ketone moiety *via* reaction with 2-bromophenylhydrazine

To confirm the presence of a ketone moiety in the first reaction product, reactions from standard turnover assays were quenched and then incubated with 1 mM of 2-bromophenylhydrazine, which reacts selectively with ketones in solution to form a phenylhydrazone (Fig. 3*A*). After 1 h of incubation, the reactions were monitored by HPLC-MS. MS results showed the formation of a new m/z of 700.8, with the characteristic isotope envelope of a brominated compound (Fig. 3*G*). The expected m/z of the hydrazine addition to the UDP-4”-keto-pentose is 701.0 (Fig. 3*A*). This new mass was analyzed by MS/MS, and the characteristic UMP fragment of m/z 323.1 was noted (Fig. 3*G*, inset). These data suggest that one of the products of the EvdS6 reaction was able to form a hydrazone, confirming the presence of a product with a ketone moiety.

### EvdS6 produces NDP-4”-keto-D-xylose and NDP-D-xylose

**Figure 4:**
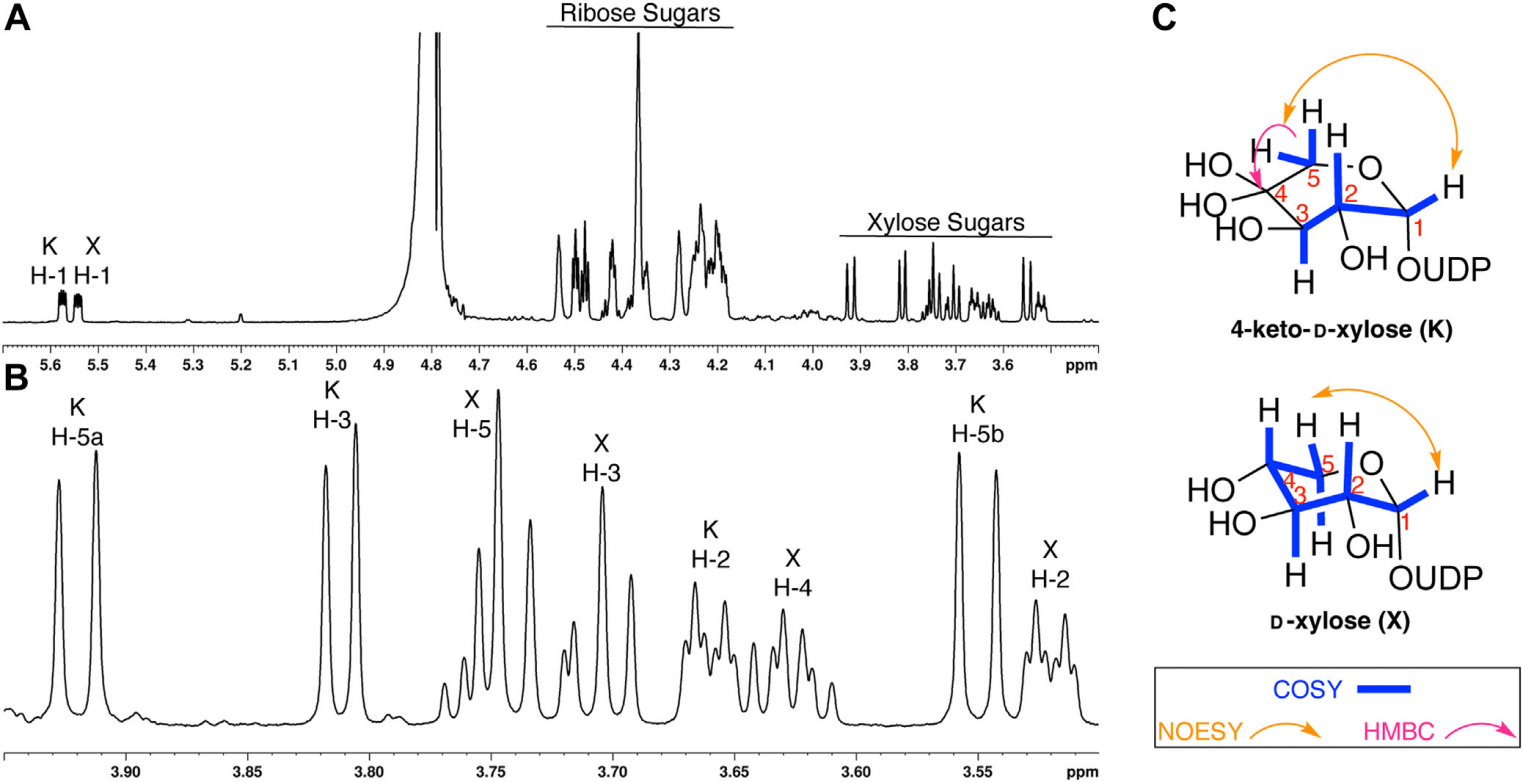
**NMR characterization of the EvdS6 UDP-pentose products.***A*, ^1^H NMR of the reaction mixture zoomed from the pentose anomeric protons to the xylose sugar protons (Full spectrum in Fig. S5). The ribose sugar protons of the two products, NAD^+^, and NADH are generally labeled. The xylose sugar protons are also labeled. *B*, ^1^H NMR zoomed in on only the xylose sugar proton peaks from 4.0 ppm to 3.5 ppm. K refers to UDP-4”-keto-D-xylose and X refers to UDP-D-xylose. *C*, NMR correlations noted from 2D spectra including COSY, NOESY, and HMBC experiments are summarized. Numbering is associated with numbering in the spectra shown.

The biosynthesis of EVD is predicted to require two different pentose sugar precursors, L-lyxose and 4-keto-D-xylose. Mass spectrometric analysis and hydrazone derivatization studies indicated that the two major EvdS6 enzymatic reaction products are consistent with a reduced UDP-pentose and an oxidized UDP-keto-pentose product, observed primarily as the hydrate in solution (Fig. 3). Therefore, it was essential to establish the structure of the reaction products of EvdS6, including stereochemistry, to map the relationship of the products to the EVD scaffold. Unfortunately, the reaction products could not be separated using C_18_ HPLC and were unstable during purification using ion exchange chromatography. Reaction products remained stable in the solution for days but were unstable during concentration or lyophilization. To facilitate comprehensive structural analysis, a 600 μl scale enzymatic reaction of EvdS6 was incubated for 20 h, the enzyme was removed using a 3 K molecular weight cutoff centrifuge filter, and 100 μl D_2_O was added prior to NMR analysis for deuterium locking and shimming. The water signal was suppressed using a pre-saturation scheme and spectra were analyzed to deconvolute the reaction components. The reaction mixture spectrum possessed two anomeric pentose sugar peaks in addition to the ribose sugars (Fig. 4*A*). COSY analysis traced spin systems from the H-1” of each pentose sugar product. In the case of the reduced pentose, the spin system traced the entire five-carbon network. Coupling values, measured *via* ^1^H-NMR and using spectrum resulting from a 2D-J-resolved pulse sequence, as well as selective 1D-TOCSY indicated that the configuration of the fully reduced sugar was UDP-D-xylose. Long-range atom connectivities were confirmed by NOESY and HMBC cross-peaks (Fig. 4*C*). The keto-pentose sugar was comprised of two spin systems, a 3-carbon framework from the H-1” and a H-5” methylene. The H-5” methylene could be connected to the H-1” spin system *via* NOESY interactions from H-1” to H-5” and HMBC interactions to the geminal hydroxy hydrate carbon at 95.4 ppm (Fig. 4*C*). Analysis of J-coupling of vicinal protons indicated that the configuration of this sugar was UDP-4”-keto-D-xylose. Correlation tables and spectra for these compounds are found in Supplementary information (Table S3 and Figs. S5–S19) and match reported values (32, 35). Notably, the UDP-4”-keto-D-xylose (**2**) product is the expected precursor for the H-ring of EVD, with a TPP-dependent acetyl transfer forming the branching backbone, similar to YerE in the biosynthesis of yersiniose A, and methylenedioxy bridge formation predicted to result in the final eurekanate structure (21). The reduced sugar, UDP-D-xylose (**3**), is a potential precursor to the G-ring, with two epimerization reactions at C-3” and C-4” resulting in the final UDP-L-lyxose (**4**) structure.

### EvdS6 exhibits the SDR enzyme architecture

**Figure 5:**
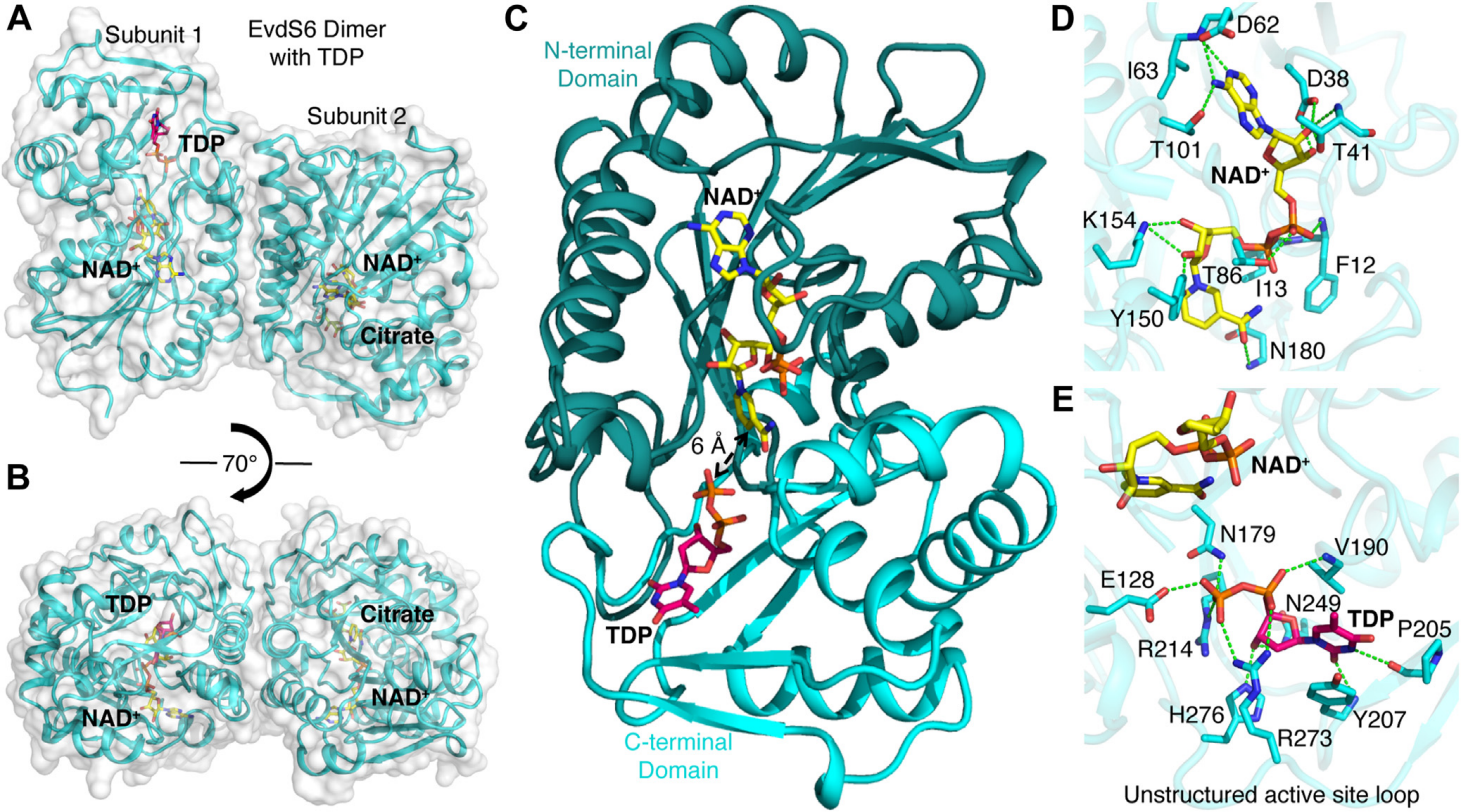
**The structure of EvdS6 co-crystallized with TDP.***A*, structure of EvdS6 dimer with co-factor NAD^+^ and TDP is shown in ribbon (*cyan*) and space-filling (*white*) representation. The co-factor NAD^+^ is shown as a *stick* representation in *yellow*. The ligand TDP is also shown as a *stick* representation in *magenta*. The citrate is shown as a *stick* representation in *green*. *B*, alternative view of EvdS6 dimer rotated 70° around x-axis as shown in (*A*). *C*, ribbon representation of a monomer of EvdS6. The N-terminal domain composed of residues between V1-Y181 and V219- A242 is shown in *dark cyan*. The C-terminal domain between residues G182-H218 and G243-L323 is shown in *cyan*. The co-factor NAD^+^ is wedged into the N-terminal domain while ligand TDP is bound at the interdomain interface. *D*, residues of N-terminal domain of EvdS6 (represented as *stick*, *cyan*) involved in H-bonding interactions with co-factor NAD^+^. *E*, residues of EvdS6 (represented as *stick*, *cyan*) involved in H-bonding interactions with TDP. Active site residues R273 and H276 belong to an unstructured region between V270-D284.

X-ray crystallographic studies were undertaken to assess how the architecture of EvdS6 supports the production of both UDP-4”-keto-D-xylose and UDP-D-xylose. Structures of EvdS6 were determined, with copurified NAD^+^, either without additional ligand or with nucleotide diphosphate (Table S4). In these EvdS6 crystal structures, the enzyme crystallized with two protein chains in the asymmetric unit. At a global level, EvdS6 displays the SDR enzyme architecture and is a dimer with a buried surface area of 2371 Å^2^ and 2362 Å^2^ in ligand-free and nucleotide diphosphate-bound states, respectively (Figs. 5, *A* and *B*, and S20). In line with the SDR architecture, each protomer consists of two domains: the N-terminal domain consisting of residues V1-Y181 and V219-A242 and the C-terminal domain between residues G182-H218 and G243-L323 (Fig. 5*C*). The N-terminal domain is organized around a typical Rossmann fold, which consists of seven parallel β-strands alternating with seven α-helices. The two major α-helices in the N-terminal domain run anti-parallel between the subunits, establishing interactions strong enough for the dimerization of this enzyme (Fig. S21). The overall structure of EvdS6 aligns closely with other members of the SDR enzyme superfamily, especially around the Rossmann fold of the N-terminal domain (41).

### The EvdS6 active site is located at the domain interface

The active site of EvdS6 is formed by residues from both N-terminal and C-terminal domains. The cofactor NAD^+^ is non-covalently bound with the adenine, ribose, and phosphate groups interacting strongly with the N-terminal domain and the nicotinamide ring located at the interface of the N- and C-terminal domains (Figs. 5, *C* and *D* and S26). To better understand the mechanism of catalysis, the commercially available UDP-GlcA was co-crystallized with EvdS6. This resulted in the appearance of new electron density in the active site of each of the two protomers in the dimer. Notably, the electron density differed substantially in the two molecules of the dimer. Surprisingly, the electron density at the active site of the first protomer is clearly consistent with TDP rather than UDP, but no density for the glucuronic acid was observed. TDP density was not observed in to *apo*-enzyme structure and its m/z was not detectable in the stock solution of commercially obtained UDP-GlcA *via* HPLC/MS, suggesting it may have been present as a minor contaminant. The relevance of TDP to the reaction is not known, but these data could suggest that EvdS6 preferentially binds to TDP-sugars.

In the second molecule of the dimer, electron density suggested that the different EvdS6 molecules in the crystal were bound to different species, resulting in a mixed state. Although the density was not readily interpretable, it appears that about 40 to 50% of the molecules in the crystal are likely bound to citrate from the buffer (Fig. S27). In addition, there appeared to be multiple binding positions for NDP-linked sugars, with two distinct positions for a five-membered ribose and one distinct position for a six-member ring clearly observed in the density (Fig. S28). Positioning the ribose or glucuronic acid of UDP-GlcA onto these clear rings did not find positions for the substrate that could easily be reconciled with the electron density, suggesting that these species may be reaction intermediates in the process of turnover (Fig. S28). The position of the TDP and this unassigned electron density allow the nucleotide diphosphate-binding residues to be identified and help to suggest the active site residues important for catalysis.

**Figure 6:**
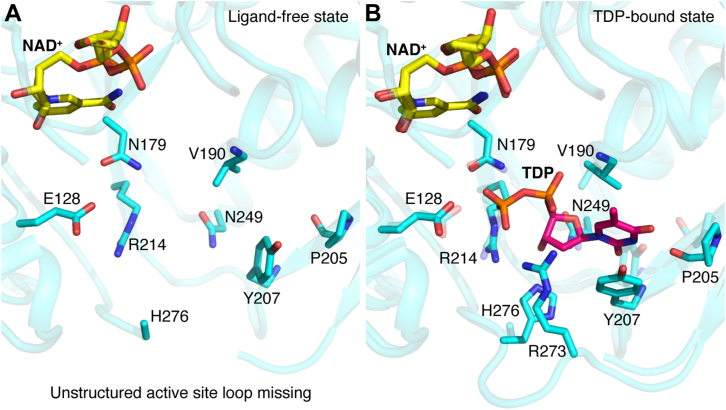
**Active site architecture of EvdS6 in the ligand-free and TDP-bound states.***A*, active site of EvdS6 in the ligand-free state. The active site residues of EvdS6 are represented as *stick* in *cyan*. In the ligand-free state, some residues of the unstructured active site loop become highly flexible as interpreted due to the lack of any traceable electron density for R273-G275. *B*, superimposed view of TDP-bound and ligand-free active sites of EvdS6. The residues of the TDP-bound active site are represented as a *stick*, *cyan* while residues of the ligand-free active site are represented as a semitransparent *stick*, *cyan*. NAD^+^ is shown in *yellow* and TDP is shown in *magenta*.

In terms of the nucleotide diphosphate-binding residues, the TDP binds at the interdomain interface of EvdS6 (Figs. 5, *C* and *E*, and S24) and has multiple direct interactions with residues of both the N-terminal domain (E128 and N179), and the C-terminal domain (V190, P205, Y207, R214, N249, R273, and H276). The thymine ring of TDP forms a hydrogen bond through its carbonyl oxygen at position two with the backbone amide of Y207; additionally, this aromatic side chain participates in a parallel π-stacking interaction with the thymine ring of TDP (Fig. 5*E*). The carbonyl oxygen of P205 is at a hydrogen bond distance to N3 of TDP (Fig. 5*E*). The ribose 3′ hydroxyl group of TDP is located at ∼2.8 Å from the -NH_2_ of N249 and H276 (Fig. 5*E*). The phosphoryl oxygens interact with V190, R273, E128, N179, and R214 (Fig. 5*E*). Notably, the binding of TDP is associated with the structuring of a loop between V270 and D284; direct interaction between the side chains of R273 and H276 with TDP facilitates this ordering (Figs. 6 and S22). In the ligand-free structure, no traceable electron density for residues R273, K274, or G275 or the sidechain of H276 was observed in one of the subunits (Figs. 6*A* and S25). The change from disordered to directly interacting with the TDP suggests that this flexible loop could be involved in binding and coordinating the substrate in the active site (Fig. 6*B*).

The position of TDP with respect to NAD^+^, the location of the mixed electron density, and the locations of residues conserved across the entire family suggests the identity of some of the residues necessary for decarboxylation catalysis (Fig. 6). Here, the catalytic dyad common to all SDR, YxxxK, contains Y150 and K154. The Y150 of this motif is positioned between the nicotinamide ring of NAD^+^ and the phosphoryl groups of TDP (Figure 5, Figure 6, and S23). Y150 is approximately 5.1 Å from the nicotinamide ring and 7.4 Å from the β-phosphoryl group of the bound TDP ligand. This β-phosphoryl group is about 7.5 Å from the nicotinamide ring as well. In close physical proximity to Y150 within the active site is T126, with significant sequence identity across the subclass it is believed to be the threonine that forms the SDR catalytic triad Tx_n_YxxxK. T126 is approximately 4.6 Å away from the nicotinamide ring and 4.2 Å from Y150. There are two other residues in the active site with homology across the subclass: E128 and N179. E128 is 6.0 Å from the nicotinamide ring and 3.4 Å from the TDP β-phosphoryl group. N179 is 6.4 Å from the nicotinamide ring and 3.5 Å from the β-phosphoryl group of TDP. From this, Y150, T126, E128, and N179 are proposed to contribute to binding glucuronic acid as well as catalyzing the decarboxylation (Fig. S23).

### Active site residues modulating C-4” reductive half of UDP-D-xylose synthase

**Figure 7:**
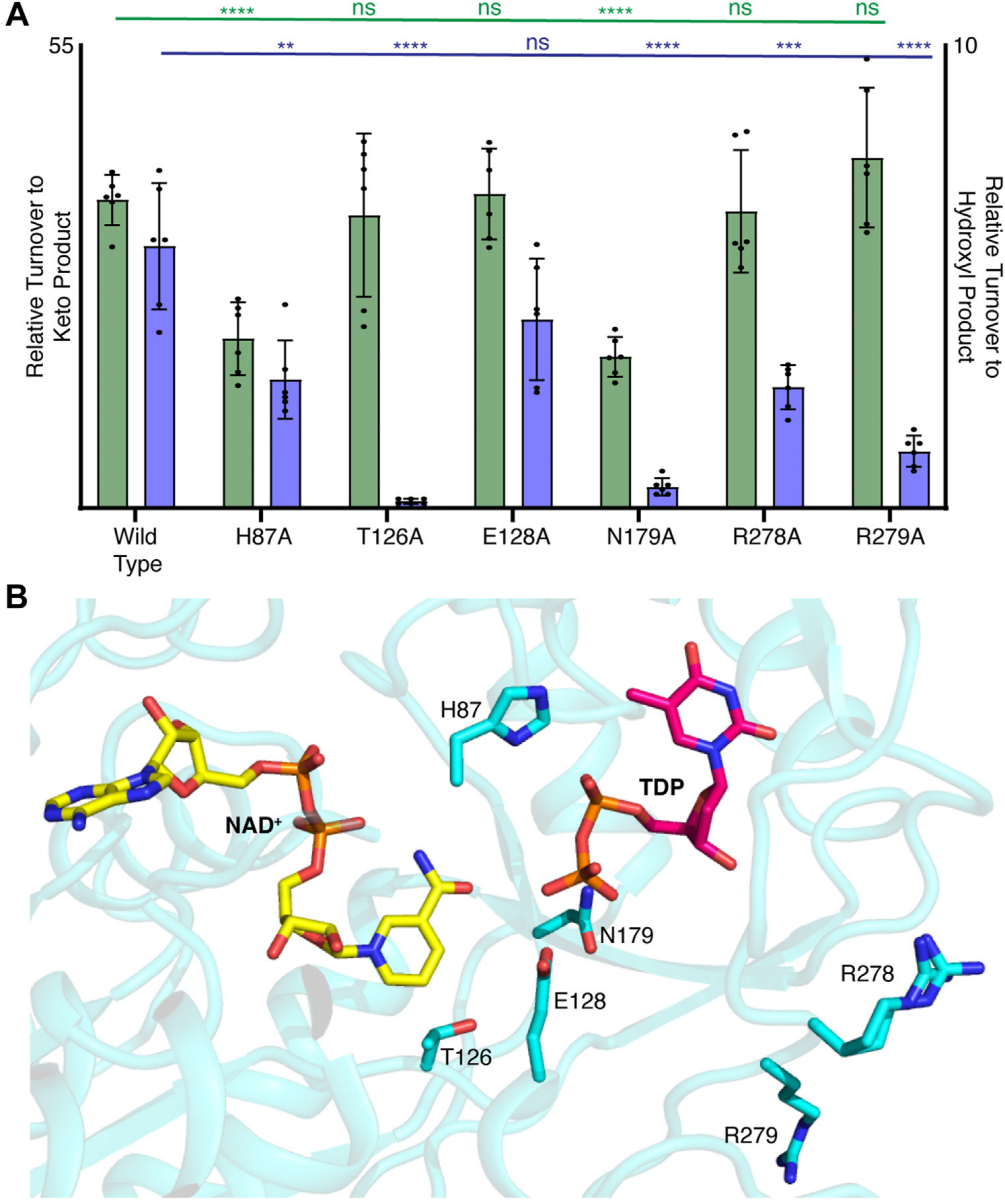
**Turnover of EvdS6 mutants.***A*, relative turnover of the mutants at 30 min is compared to wild-type EvdS6 turnover. Oxidized product in *green*, *left* axis; reduced product in *blue*, *right* axis. Averages of six MS experiments, three biological replicates each with two technical replicates. Percentage of turnover was calculated using the area under the curve of the EICs for the starting material and both products. Reactions were set up with 1 mM UDP-GlcA, 1 mM NAD^+^, 50 mM HEPES pH 8.5. Reactions were incubated for 30 min to achieve less than 50% turnover. Statistical analyses were done using a two-way *t* test comparing mutant turnover values to wild-type turnover within each product. ∗∗∗∗ denotes *p* < 0.0001; ∗∗∗ denotes *p* < 0.0002; ∗∗ denotes *p* < 0.0021; ∗ denotes *p* < 0.0332; ns denotes not statistically signficant. *B*, EvdS6 active site with mutated residues shown. TDP is shown in *magenta*, NAD^+^ is shown in *yellow*.

Six EvdS6 mutants were generated based on sequence analysis and the crystal structures reported above. A detailed sequence alignment implicated T126 in the C-4” oxidation and reduction steps, based upon a review of the SDR superfamily catalytic triad (23, 24). Upon mutation of T126 to alanine, there was no noted change in the amount of the oxidized UDP-4”-keto-D-xylose product produced; however, there was minimally detectable formation of the reduced product, UDP-D-xylose, (Fig. 7*A*). This implicates T126 in the NADH-dependent reduction event. The β-hydroxyl of T126 could coordinate the intermediate C-4” ketone to facilitate the hydride transfer. The residue R279 aligned with R619 of ArnA; however, in the TDP-bound EvdS6 crystal structure, R279 is a significant distance away from the putative sugar-binding pocket. The mutation of R279 to alanine did not cause a change in the production of the oxidized product, UDP-4”-keto-D-xylose. The R279A mutation did result in a decrease in the production of the reduced product, UDP-D-xylose. Neighboring R279 is another arginine, R278, which was also mutated into an alanine. The R278A mutant did not significantly alter the production of the oxidized product, UDP-4”-keto-D-xylose. Though not as significant as R279A, the production of the reduced UDP-D-xylose was lowered when compared to the wild type. Both arginine residues are located on the flexible active site loop in the structure (Figs. 7*B* and S25), which may be involved in substrate binding, as supported by the TDP-bound structure. The mutations of these arginine residues could increase the mobility of this loop and result in the faster release of the oxidized product, thus reducing the formation of the reduced product.

To investigate and identify interactions involved in decarboxylation, an additional three residues were mutated based on the crystal structure of EvdS6. Mutation of H87 to alanine led to a change in overall turnover, achieving only about 20 % turnover to the oxidized product compared to the 36 % turnover of the wild type in the same 30 min time frame. However, this mutation did not alter the ratio of the oxidized and reduced products produced, as seen with the other mutations. H87 has no significant sequence similarity across the subclass of decarboxylases; this mutation might induce moderate structural changes in the active site that reduce catalytic potential. The mutation of E128 to alanine did not result in any significant changes to EvdS6 turnover, which was unexpected due to its sequence identity across the SDR decarboxylases and its proximity to the postulated sugar-binding site. However, this lack of notable change in activity could be due to the presence of D127, which is an atypical residue at this position in glucuronic acid decarboxylases. Most decarboxylases have a serine residue at this position, making a TSE triad whereas EvdS6 has a TDE triad. This glutamate residue has been suggested to coordinate water for the C-5” protonation step (40). Though D127 does not appear to be directly involved in the active site as seen in the crystallography data, it is possible that in the E128A mutant D127 shifts to coordinate a water molecule, which could explain why there was not a significant change in activity in this mutant. Because of the lack of observed interactions in the active site and the lack of sequence identity of D127, this residue was not targeted in this initial study. Mutation of N179 to alanine also leads to a loss of turnover, again only achieving about 20 % turnover to the oxidized product as compared to the 36 % turnover noted for the wild-type EvdS6. More interestingly, there was minimal evidence of the reduced UDP-D-xylose moiety produced in this mutant. This also suggests that N179 could be involved in the NADH-dependent reduction event, along with T126.

## Discussion

The ongoing efforts to develop EVD for the clinic will be advanced by the ability to modify the parent compound to optimize its pharmacological properties. Critical to synthetic biology approaches to modifying the eastern half of the molecule is understanding the origins of the G- and H-ring pentose sugars in EVD, identifying the glucuronic acid decarboxylase enzyme, determining the stereochemistry of the reaction product, and understanding the structural determinants of activity. Here, sequence analysis identified EvdS6 as a likely glucuronic acid decarboxylase candidate belonging to the SDR superfamily (Figs. 2*C* and S1). Enzymatic characterization demonstrated that this enzyme produces a mixture of two products at comparable levels *in vitro*, and these products differ by 2 Da (Fig. 3). Bromophenyl hydrazone derivatization confirmed the presence of a ketone in one of the products, and NMR studies enabled the stereochemical determination of the two products as UDP-4”-keto-D-xylose and UDP-D-xylose (Figs. 3*F* and 4). The structures of EvdS6 in the ligand-free and TDP-bound states informed mutagenesis studies, which, in turn, revealed important active site residues for the reaction (Figs. 6 and 7).

Comparison of the EvdS6 active site to the active sites of ArnA and hUxs1, the two other decarboxylases for which structures have been elucidated, shows minimal variation, especially in the positioning of the side chains of the catalytic triad Tx_n_YxxxK (Fig. S23). These three residues, along with most of the residues immediately interacting with the cofactor and substrate, overlap in their orientation despite significant sequence deviation (Fig. S24). Although ArnA produces the 4-keto-pentose, hUxs1 produces the reduced pentose, and EvdS6 produces a mixture of the two reaction products, it was not clear from the structures why the reaction products differ between enzymes of this subclass (Figs. S23 and S24). It is possible that the distinguishing factor in producing the oxidized 4-keto-pentose instead of the reduced pentose has less to do with the active site architecture and more to do with the relative kinetics of the oxidative decarboxylation and reduction reactions, and with the rate of release of the oxidized product occurring faster than the reduction half of the reaction in some enzymes. One possibility is related to a flexible active site loop that gains order upon substrate binding (Fig. S25). Because this loop coordinates the substrate, a change in dynamics may control the rate of release of the oxidized product. It is possible that greater flexibility in this loop allows for faster release of the 4-keto-pentose product. Evidence in support of this was observed in the mutagenesis of the flexible loop residues R279 and R278 to alanine, which both produced significantly less of the reduced UDP-D-xylose than the wild-type enzyme (Fig. 7). This suggests the structure of this loop might play a role in the release of UDP-4”-keto-D-xylose. Mutagenesis experiments did not highlight a single catalytic residue implicated as essential for decarboxylation; however, two residues, T126 and N179, were critical for the subsequent reduction that distinguishes the two products. Indeed, the T126A and N179A mutants both had a nearly complete loss in the production of UDP-D-xylose (Fig. 7) One possibility is these residues are involved in the hydride transfer from NADH to the substrate.

**Figure 8:**
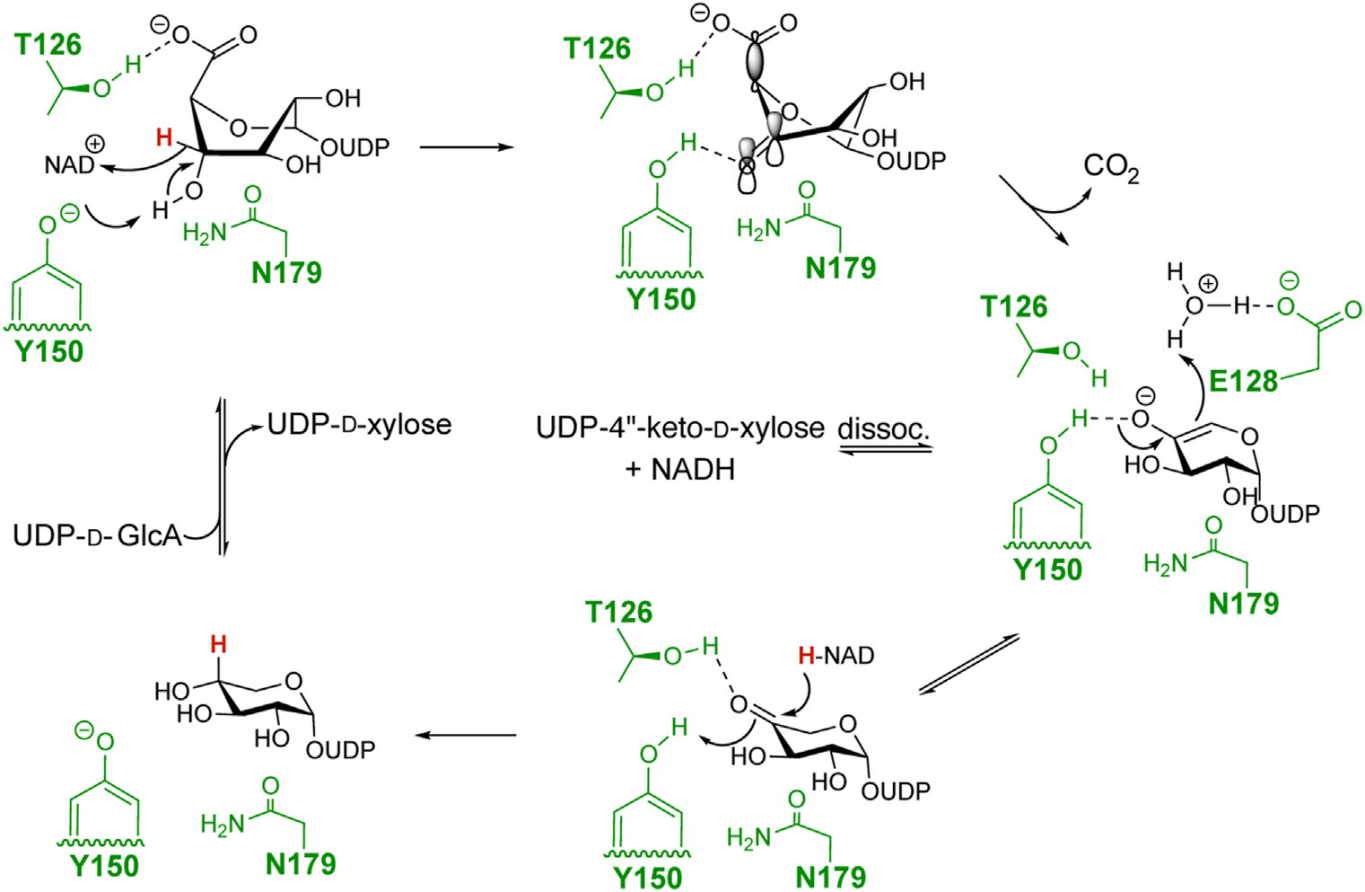
**Decarboxylase mechanism.** Potential mechanism of the decarboxylase reaction catalyzed by EvdS6 highlighted residues shown in *green*. The Michaelis complex is shown binding the boat conformation and the first intermediate depicts the twist boat confirmation, which establishes optimal orbital overlap between the C-5”-C-6” σ-bond and the C-4”-O π-bond that is required for decarboxylation.

Structural and mutagenesis studies herein contribute to an ongoing understanding of the mechanism of glucuronic acid decarboxylases. In the absence of co-structural data with bound NDP-glucuronic acid, mechanistic proposals are speculative. Based on previous structural and mutagenesis studies of the SDR superfamily, the tyrosine residue of the YxxxK catalytic dyad motif is proposed to be responsible for the deprotonation of the substrate C-4” hydroxyl group, enabling the transfer of the C-4” methine hydrogen to the NAD^+^ cofactor (Fig. 8) (22, 23). Based upon structural, mutagenesis, and molecular dynamics studies, Eixelsberger and coworkers proposed that conformationally induced stereo-electronic effects are important contributors to decarboxylation catalysis (27). In this regard, the breaking C-5”-C-6” σ-bond must be aligned with the newly established π-bond of the C-4” ketone for the elimination of CO_2_ and the generation of the pentose enolate intermediate (Fig. 8). Thus, a conformational change to the twist-boat conformer, in the related human enzyme, hUxs1, promotes this decarboxylation event by optimally aligning these bonds (40, 42). The enolate intermediate is then protonated at the C-5” position by an active site water molecule, which may be coordinated by E128. Finally, in the reductive half of the typical catalytic cycle, after decarboxylation and C-5” protonation, the hydride is transferred back to C-4” from the NADH cofactor. Mutagenesis results reported here implicate T126 in this reduction reaction. It is possible that the β-hydroxyl of T126 coordinates the ketone making it activated for hydride transfer. This is in agreement with the proposal that the analogous threonine in hUxs1 coordinates the ketone after decarboxylation (40). Additionally, the N179A mutation resulted in a decrease in overall turnover and specifically produced minimally detectable UDP-D-xylose. N179 could potentially be involved in the orientation of the substrate in the active site, especially after the C-6” decarboxylation event. Alternatively, it is possible that the mutation of this residue allowed for significant expansion of the active site cavity to allow for a water molecule to hydrate the C-4” ketone, which would impede the reduction event. It is of note that the mutation of H87, which is not conserved in other similar decarboxylases, led to a reduction in the overall turnover to both products. This residue is in close proximity to the sugar-binding pocket and thus its mutation might have hindered the conformational change necessary to eliminate CO_2_. Future studies with bound NDP-glucuronic acid will aid in establishing the degree to which conformation and proposed active site interactions play a role in catalysis.

EvdS6 produces comparable levels of the two reaction products over the course of typical reaction times (up to 2 h) *in vitro*. It has been proposed that the enzymes that release the keto product might bind NAD^+^ less tightly, with fewer contacts, and ultimately release both NDP-4”-keto-D-xylose and the cofactor NADH (43). However, with extended reaction timing and depletion of the NDP-GlcA substrate, NDP-4”-keto-D-xylose can re-enter the active site and drive the reaction to the formation of NDP-D-xylose. This was observed with ArnA, which was previously thought to only produce the keto product but extended incubation showed that ArnA will produce detectable levels of the reduced product, and conversely with CalS9, which was previously shown to only produce the reduced product but was later confirmed to release the oxidized NDP-4”-keto-pentose (39, 43). The dissociation of the keto-product is required by the data herein, which showed that UDP-4”-keto-D-xylose is produced and released first but with extended (overnight) reaction UDP-D-xylose becomes the predominate product of EvdS6.

In the biosyntheses of the enediyne calicheamicin and indolocarbazole AT2433, the enzymes CasS9 and AtmS9, respectively, produce the 4-keto-pentose sugar for transamination by subsequent pyridoxal phosphate-dependent enzymes to produce a 4-amino-pentose product (39). ArnA, in the biosynthesis of lipid A of *E. coli*, also requires the 4-keto-pentose in order to be transaminated to the 4-amino-pentose (31, 32). Conversely, in the biosynthesis of avilamycin, a highly related orthosomycin family member, which only contains the pentose sugar L-lyxose, AviE2 was shown to exclusively produce the reduced D-xylose product. This would also need to undergo epimerization at C-3” and C-4” to form L-lyxose (33). This supports that the biosynthesis of L-lyxose in everninomicin D may also begin with D-xylose (Fig. 1, path a). However, reduction/epimerization of the 4-keto-pentose product (**2**, path b) by one of the remaining unassigned SDR enzymes cannot be ruled out at this time.

The biosynthesis of the eurekanate of everninomicin D is distinct from other branched-chain sugars in that it begins with a pentose-generating decarboxylase enzyme. In addition to the H-ring of avilamycin, other sugars that are branched by the addition of an acetyl group are hexose-derived, and the keto-sugar precursor is a product of 4,6 dehydratases. This can be seen in the proposed biosynthesis of the sugars of kosinostatin and trioxacarcin (44, 45). The biosynthesis of a sugar branched by the addition of an acetyl moiety that is pentose-derived is thus unique to the everninomicin D pathway.

**Figure 9:**
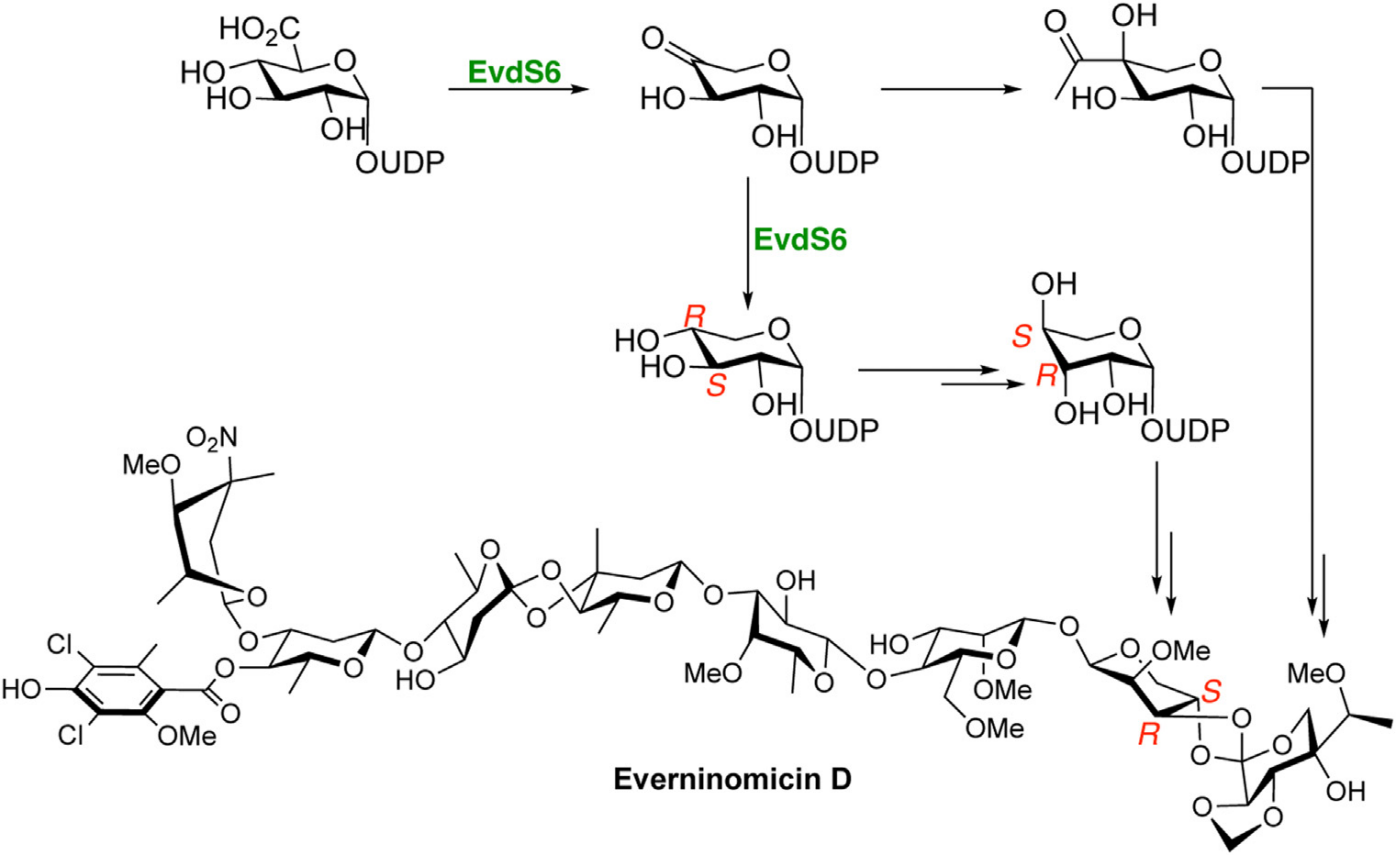
**Potential relationship of EvdS6 products to the G- and H- rings of everninomicin D.** EvdS6 produces both UDP-4”-keto-D-xylose, which can be transformed into the eurekanate, and UDP-D-xylose, which might be transformed into L-lyxose.

In producing both the oxidized UDP-4”-keto-D-xylose and the reduced UDP-D-xylose, EvdS6 potentially produces sugar precursors for both pentose-derived sugars of the EVD scaffold, the G- and H-rings (Fig. 1*B*). To complete the pathway, a series of epimerization reactions of UDP-D-xylose, at C-3” and C-4” or reduction/epimerization of UDP-4”-keto-D-xylose, would be required to provide the G-ring sugar, L-lyxose (Fig. 9). Within the *evd* gene cluster, there are seven other SDR enzymes, whose functions have not yet been assigned, that could possibly catalyze these epimerization reactions (Fig. 2*B*). The UDP-4”-keto-D-xylose moiety is the hypothesized substrate for the TPP-dependent addition of the branching acetyl group of the H-ring sugar, eurekanate (Fig. 9), in analogy to the biosynthesis of yersiniose A (21). A putative TPP-dependent heterodimeric enzyme, comprised of EvdU5/EvdS3, which has significant sequence identity to pyruvate dehydrogenase, is hypothesized to catalyze this transformation. Future work in our laboratory will validate EvdU5/EvdS3 in eurekanate formation as well as the timing of C-acetylation in relation to the glycosyltransferase, methyltransferase, and oxygenase activities required in eurekanate elaboration. Likewise, the precursor relationship of D-xylose to L-lyxose can be validated by identifying the epimerases required. By providing the potential precursors to the G- and H-rings of EVD, EvdS6 is integral to the early biosynthesis of this complex octasaccharide. This work elucidates one of the early biosynthetic steps in the pathway toward EVD and in clarifying the products of EvdS6 informs the remaining biosynthetic transformations required to form the sugars of the eastern terminus, L-lyxose and eurekanate.

## Experimental procedures

### General methods and bacterial culture conditions

All reagents were obtained from Sigma Aldrich unless otherwise specified. UDP-Glucuronic acid was obtain from Biosynth. Gibson Assembly kit and restriction enzymes were purchased from NEB. DNA and protein concentrations were determined using a Qubit 4 fluorometer (Thermo Fisher Scientific) and reagents from Invitrogen. The bacterial strains used in this study are listed in Table S2. *E. coli* Tuner (DE3) cells (Novagen) heterologously expressing *evdS6* were grown using LB agar (10% tryptone, 5% yeast extract, 10% NaCl, and 2% agar) or LB broth (10% tryptone, 5% yeast extract, and 10% NaCl) with kanamycin (50 μg/ml) to maintain pET-EvdS6 vector.

### Cloning

*EvdS6* was cloned by Gibson Assembly using the NEB Gibson Assembly kit. The *evdS6* gene was amplified using the PCR with the primers EvdS6_For and EvdS6_Rev, listed in Table S1. The PCR product was purified from a 1% agarose gel using Qiagen’s QiaQuick Gel Extraction Kit. The pET28a(+) vector backbone was obtained by digestion of a previous expression plasmid pET-EvdM5 (16). pET-EvdM5 was miniprepped from 3 ml of *E. coli* Top10 culture using the Qiagen QiaPrep Spin Miniprep Kit and then linearized by restriction enzyme digest using BamHI and NdeI. The vector restriction digest was purified from a 1% agarose gel using the QiaQuick Gel Extraction Kit. The linearized vector backbone and the *evdS6* PCR product were ligated following the NEB Gibson Assembly protocol as published using 64 ng of the linear pET28a(+) vector and 160 ng of the *evdS6* PCR cassette. After incubation in the thermocycler at 50 °C for 60 min, the Gibson Assembly reaction mixture was transformed into the chemically competent *E. coli* 5-alpha cells included in the NEB kit using heat shock at 42 °C for 30 s. Transformed cells were plated on LB with a final concentration of 50 μg/ml kanamycin and incubated at 37 °C overnight. Individual colonies were then inoculated into liquid LB with 50 μg/ml kanamycin and grown overnight shaking at 37 °C. Cultures were miniprepped using the Qiagen QiaPrep Spin Miniprep Kit and plasmids were sequenced by GenHunter Corporation to confirm the correct assembly. Glycerol stocks were made with the *E. coli* 5α cells containing the confirmed pET-EvdS6 plasmid by resuspending pelleted cells in 600 μl of 20 % glycerol.

### Expression

A culture of *E. coli* 5α (NEB) containing pET-EvdS6 was grown overnight in LB with 50 μg/ml kanamycin. This culture was miniprepped using Qiagen’s QiaPrep Spin Miniprep Kit to obtain fresh samples of the pET-EvdS6 plasmid. The pET-EvdS6 plasmid (5 μl) was transformed into Tuner (DE3) competent cells by electroporation using the BioRad GenePulser Xcell (2 mm cuvette, 2.5 kV, 25 μF, and 200 Ω) and plated on LB agar with 50 μg/ml kanamycin for selection. Colonies were grown out in LB broth overnight at 37 °C and the presence of the pET-EvdS6 plasmid was confirmed by miniprep and digestion using the restriction enzymes XbaI and HindIII. Following plasmids confirmation, 0.5 l LB broth in 2 l Fernbach flasks were inoculated at 1% with the overnight culture. The 0.5 l cultures were grown at 37 °C until they reached an OD_600_ of 0.6, at which point they were induced with 0.1 mM of IPTG. After induction, expression cultures were grown overnight at 16 °C. Cultures were harvested by centrifugation at 3000*g* and stored at −20 °C until purification.

### Purification

Cell pellets from the above expression cultures were resuspended in 25 ml of lysis buffer (50 mM Tris HCl pH 7.5, 100 mM NaCl, 50 mM imidazole), and 5 mg/ml of lysozyme was added. The resuspended sample was frozen overnight at −80 °C. The sample was thawed at room temperature and then further lysed by passage through French Press (three passages, pressure maintained between 16–25 kpsi). After lysis, the sample was centrifuged at 70,000*g* for 30 min. The supernatant was filtered through a 0.45 μM PES filter (Genesee Scientific) prior to loading onto an equilibrated Cytiva His-Trap FF Crude column on a Bio-Rad NGC Quest 10 chromatography system (equipped with a System Pump 10 and a single UV wavelength and conductivity monitor). The column was washed with five column volumes of buffer A (50 mM Tris HCl pH 7.5, 100 mM NaCl, 50 mM imidazole) before the protein was eluted using a 0 to 100% gradient of elution buffer B (50 mM Tris HCl pH 7.5, 100 mM NaCl, 500 mM imidazole) over the course of 10 column volumes. As monitored by A_280_, fractions with protein present were combined and concentrated using a 10 kDa MWCO Amicon centrifugal filter. The concentrated sample was then passed through a GE HiTrap Desalting column that was preequilibrated with storage buffer (50 mM HEPES pH 8.5, 100 mM NaCl, 10% glycerol). Fractions containing protein but minimal conductivity were pooled and further concentrated using a 10 kDa MWCO Amicon centrifugal filter. The final sample was divided into 250-μl aliquots and flash-frozen using a bath of dry ice and ethanol. Final purified protein samples were stored at −80 °C until used in enzymatic assays.

### Turnover assays

Unless otherwise stated, all turnover assays were set up in the following way. Stock solutions of 500 mM HEPES pH 8.5, 50 mM UDP-GlcA, and 50 mM NAD+ were prepared. These stock solutions were then combined and diluted using sterile water to a final concentration of 50 mM HEPES pH 8.5, 1 mM UDP-GlcA, and 1 mM NAD+ in a total reaction volume of 100 μl. Protein from a freshly thawed sample was added to the reaction last to a final concentration of 0.5 mg/ml. Reactions were allowed to incubate at 30 °C in a dry heat block for 1 h unless otherwise stated. Reactions were quenched by the addition of 100 μl methanol, incubated on ice for 10 min, and then centrifuged at 13,000*g* for 10 min. Samples were then analyzed *via* mass spectrometry.

### HPLC-MS analysis

Turnover reactions were monitored by HPLC-MS using a Hypercarb column (5 μm, 3 mm × 50 mm, Thermo Fisher Scientific). Replicates of 20 μl samples were injected using the ThermoPal autosampler into the HPLC system. The sample was then eluted using the following HPLC gradient running at 300 μl/min using buffer A: 95 H_2_O: five Acetonitrile, 10 mM ammonium acetate, 1% diethylamine; buffer B: five H_2_O: 95 Acetonitrile, 10 mM ammonium acetate, 1% diethylamine: 0 to 5 min 100 % buffer A, 5 to 15 min linear gradient 0 to 50 % buffer B, 15 to 16 min linear gradient to 100 % buffer B, 16 to 21 min 100 % buffer B, 21 to 22 min linear gradient to 100 % buffer A, 22 to 30 min 100 % buffer A. The HPLC was then directly connected to a TSQ Quantum Access Max (Thermo Scientific) mass spectrometer equipped with a Heated Electrospray Ionization II (HESI II) source. The mass spectrometer was operated in negative mode with 1 s scans, scanning from 200 to 1000 m/z. The electrospray needle was maintained at 3500 V and the ion transfer tube was maintained at 350 °C and 35 V.

### Reaction with 2-bromophenylhydrazine

After quenching with methanol, 200 μl of standard EvdS6 enzymatic assay reactions were used for hydrazone derivatization. The derivatization reaction was initiated by adding 2 μl of 200 mM 2-bromophenylhydrazine for a final hydrazine concentration of 1 mM. This reaction was stirred at room temperature for 1 h. The reaction was then directly monitored by HPLC-MS. A 20 μl sample of the reaction was loaded onto a C_18_ column (250 × 4.6 mm, 5 μm C_18_ 100 Å, Phenomenex Luna) using the ThermoPal Autosampler and a linear gradient from 0 to 100 % buffer B over 15 min was used to elute the sample. Buffer A: 95 H_2_O: five Acetonitrile, 10 mM ammonium acetate; Buffer B: five H_2_O: 95 Acetonitrile, 10 mM ammonium acetate. After HPLC the sample is split 750 μl going to a Accella PDA (Thermo Scientific) and 250 μl going to a TSQ Quantum Access Max (Thermo Scientific) mass spectrometer equipped with a HESI II source. The mass spectrometer was operated in negative and positive modes with 1 s scans, scanning from 200 to 2000 m/z. The electrospray needle was maintained at 3500 V and the ion transfer tube was maintained at 350 °C and 35 V.

### NMR analysis

NMR experiments were performed on Bruker AV-III 800 MHz spectrometers equipped with triple resonance cryogenically cooled probe CPTCI. 600 μl of a sample in 50 mM sodium phosphate buffer pH 8.5, 500 μl H_2_O and 100 μl D_2_O was added to a 5 mm NMR tube. All experiments were run at 298 K and chemical shifts were referenced against the 4,4-dimethyl-4-silapentane-1-sulfonic acid standard. Standard Bruker pulse sequences were applied utilizing pre-saturation of the water signal at a 50 Hz cw irradiation field for all homonuclear and the HMBC experiments. Selective excitation of the 1D TOCSY experiments was optimized and 45 to 75 ms Gaus_180 pulses were applied. NMR spectra were processed using Topspin 3.5 (Bruker BioSpin).

### Crystallography

The protein was purified *via* size exclusion chromatography using a Superdex 200 Increase 10/300 Gl column equilibrated in 25 mM HEPES, pH 7.5, and 100 mM NaCl. The protein sample was then concentrated to ∼9 mg/ml using a 30 kDa molecular weight cut-off concentrator. EvdS6 was crystallized using the hanging drop vapor-diffusion method at room temperature (∼25 °C) by combining 1 μl 9 mg/ml of the protein (25 mM HEPES pH 7.5, 100 mM NaCl) in a 1:1 ratio with the mother liquor. The mother liquid for EvdS6 crystals in the ligand-free state was composed of 0.1 M HEPES pH 7.0 and 10 % w/v PEG 6000 (Molecular Dimensions, JCSG+, C4). EvdS6 in the ligand-bound state was crystallized by incubating protein (9 mg/ml) with the substrate UDP-GlcA in a 1:10 M ratio for 30 min prior to setting up crystallization trays. Crystals were obtained by mixing 1 μl of the protein/substrate mix with 1 μl of mother liquor (0.03 M citric acid, 0.07 M BIS-TRIS propane, pH 7.5, and 20 % w/v PEG 3350 (Hampton Research, Peg/Ion HT H4)) at room temperature. Crystals were harvested, cryo-protected with 10 % of a 1:1 mixture of ethylene glycol and glycerol, and flash cooled in liquid nitrogen prior to data collection.

Diffraction data were collected at LS-CAT beamline ID-G (λ = 0.97857) equipped with MARMOSAIC 300 CCD detector at 100 K. The data were indexed, integrated, and scaled using HKL2000 (46). Unit cell parameters and data collection statistics are listed in Table S4.

Both EvdS6 structures were determined by molecular replacement using the PHASER subroutine (47) program in PHENIX (48) and the coordinates of DesIV (dTDP-glucose 4,6-dehydratase) from *Streptomyces venezuelae* (PDB entry 1R66 (49)) as the search model. Ligands (TDP and citrate) were modeled manually into the |*F*_*o*_| – |*F*_*c*_| difference electron density in COOT (50). Both the structures were refined by standard methods by alternating model building in COOT (50) and refinement in Phenix (48).

### Mutagenesis

Mutagenesis was performed using the Agilent QuikChange kit. Primers were designed following the kit protocol and using the online Agilent QuikChange Primer Design tool. Primers are all listed in Table S1. The QuikChange kit procedure was closely followed. To the reaction mixture (reaction buffer, dNTPs, QuikSolution) in PCR tubes, 50 ng of pET-EvdS6 and 125 ng of each primer were added to a final volume of 50 μl. PCR reactions were initiated by the addition of 1 μl *PfuTurbo* DNA polymerase and then incubated in the thermocycler for the following protocol: 95 °C for 30 s, 95 °C for 30 s, 55 °C for 1 min, 68 °C for 5 min, repeat steps 2 through 4 for 18 cycles. After the reactions cooled, 1 μl of Dpn I restriction enzyme was added to each reaction tube. Tubes were microcentrifuged for 1 min and then incubated at 37 °C for 1 h. Transformed each reaction into *E. coli* XL1-Blue Supercompetent cells following transformation protocol. Cells were aliquoted by 100 μl and 1.7 μl of β-mercaptoethanol was added to each aliquot. Cells were incubated on ice for 10 min. After incubation on ice, 5 μl of the mutagenesis PCR reaction was added to each aliquot of competent cells. These transformations were then incubated on ice for 30 min. Cells were heat pulsed at 42 °C for 45 s in a hot water bath then incubated on ice again for 2 min. To recover the transformations, 0.9 ml of SOC media was added to the cells and they were incubated with shaking at 37 °C for 1 h. Transformations were plated onto LB agar with a final concentration of 50 μg/ml kanamycin. Agar plates were incubated overnight before individual colonies were then inoculated into liquid LB with 50 μg/ml kanamycin. Liquid cultures were incubated with shaking at 37 °C overnight. Plasmids were isolated from cultures using the Qiagen QuikPrep Miniprep Spin Kit. Mutant plasmids were then sequenced by GenHunter Corporation (Nashville, TN). Stocks in 20 % glycerol were made of the cultures containing the correct mutant plasmids for storage at −80 °C.

## Data availability

The crystal structures have been deposited to the RCSB Protein Databank (https://www.rcsb.org/). The PDB ID for the ligand-free structure is 8SHH. The PDB ID for the TDP-bound structure is 8SK0.

## Supporting information

This article contains supporting information.

## Conflict of interest

The authors declare that they have no conflicts of interest with the contents of this article.
