# Supplementary material for: EvdS6 is a bifunctional decarboxylase from the everninomicin gene cluster
Source: J Biol Chem. 2023 Jun 5;299(7):104893. doi: 10.1016/j.jbc.2023.104893 (PMC10338323; doi:10.1016/j.jbc.2023.104893)
Supplement: Supporting Figures S1–S28 and Tables S1–S4 [file mmc1.docx]

**Supporting Information**

**EvdS6 is a bifunctional decarboxylase from the everninomicin gene cluster**

Callie C. Dulin^1^, Pankaj Sharma^2^, Laura Frigo^2^, Markus W. Voehler^1,3^, T. M. Iverson^2,4^,

and Brian O. Bachmann*^1,4^

**Table of Contents**

Table S1: Primers used for cloning and mutagenesis of EvdS6.

Table S2: Bacterial Strains used in this study.

Table S3: NMR correlation tables of the products produced by EvdS6.

Table S4: Crystallographic data collection and refinement statistics.

Figure S1: Multiple Sequence Alignment of glucuronic acid decarboxylases.

Figure S2: EvdS6 plasmid map and purification.

Figure S3: MSMS Spectra of Products.

Figure S4: Turnover optimization.

Figure S5: ^1^H spectrum of the EvdS6 reaction.

Figure S6: Expansion of ^1^H spectrum of the EvdS6 reaction.

Figure S7: Expansion of ^1^H spectrum of the EvdS6 reaction.

Figure S8: ^13^C{^1^H} spectrum of the EvdS6 reaction.

Figure S9: Selective 1D TOCSY spectrum for the peak at 5.575 ppm

Figure S10: Selective 1D TOCSY spectrum for the peak at 5.542

Figure S11: COSY spectrum of the EvdS6 reaction.

Figure S12: Expansion of the COSY spectrum of the EvdS6 reaction.

Figure S13: HSQC spectrum of the EvdS6 reaction.

Figure S14: Expansion of the HSQC spectrum of the EvdS6 reaction.

Figure S15: HMBC spectrum of the EvdS6 reaction.

Figure S16: Expansion of the HMBC spectrum of the EvdS6.

Figure S17: NOESY spectrum of the EvdS6 reaction.
Figure S18: Expansion of the NOESY spectrum of the EvdS6 reaction.

Figure S19: Homonuclear J-resolved spectrum of the EvdS6 reaction.

Figure S20: The structure of EvdS6 in the ligand-free state.

Figure S21: Interaction interface between the EvdS6 dimer.

Figure S22: Comparison of flexibility of EvdS6 crystal structures.

Figure S23: Active site residues.

Figure S24: Residues involved in substrate binding.

Figure S25: Active site loop.

Figure S26: Electron density of NAD^+^.

Figure S27: Electron density of bound ligands.

Figure S28: Unresolved electron density at the active site of EvdS6 in the ligand-bound state.

**Table S1: Primers used for cloning and mutagenesis of EvdS6.**

| Primer | Sequence |
| --- | --- |
| EvdS6_For | GCCTGGTGCCGCGCGGCAGCGTGCCGAGGGTCTTCGTGG |
| EvdS6_Rev | GTCGACGGAGCTCGAATTCGTCAGTCGTGCGGGACGGC |
| H87A_For | GCGGCCGAGACCGCCGTCGACCGGTC |
| H87A_Rev | GACCGGTCGACGGCGGTCTCGGCCGC |
| T126A_For | TACACCTCGTCGGCGGAGACCTGGACG |
| T126A_Rev | CGTCCAGGTCTCCGCCGACGAGGTGTA |
| E128A_For | GTCTCCACCGACGCGGTGTACGGCAGC |
| E128A_Rev | GCTGCCGTACACCGCGTCGGTGGAGAC |
| N179A_For | CATCACCAGGTGCGGCGCCAACTACGGTCCATAC |
| N179A_Rev | GTATGGACCGTAGTTGGCGCCGCACCTGGTGATG |
| R278A_For | GGAAAGCGAGTAGGCGCGGTCGTGGCCC |
| R278A_Rev | GGGCCACGACCGCGCCTACTCGCTTTCC |

**Table S2: Bacterial Strains used in this study.**

| Strain | Purpose |
| --- | --- |
| *M. carbonacea* var. *aurantiaca* | Everninomicin D producing strain |
| *E. coli* BL21 (DE3) - Novagen | Expression strain |
| *E. coli* Tuner (DE3) - Novagen | Expression strain |
| *E. coli* Top10 - Invitrogen | Plasmid Storage |

**Table S3: NMR correlation tables of the products produced by EvdS6.**

| **4-keto-xylose** | **^1^H ppm** | **Multiplicity** | **J Values** | **^13^C ppm** |
| --- | --- | --- | --- | --- |
| 1 | 5.575 | dd | ^3^J_1,P_ = 3.7  ^3^J_1,2_ = 6.9 | 98.6 |
| 2 | 3.660 | dt | ^4^J_2,P_ = 3.2  ^3^J_2,3_ = 10.2 | 73.3 |
| 3 | 3.812 | d |  | 75.4 |
| 4 |  |  |  | 95.4 |
| 5 | 3.920  3.550 | d  d | ^2^J_5,5_ = 13.1 | 68.2 |

| **Xylose** | **^1^H ppm** | **Multiplicity** | **J Values** | **^13^C ppm** |
| --- | --- | --- | --- | --- |
| 1 | 5.542 | dd | ^3^J_1,P_ = 3.8  ^3^J_1,2_ = 7.0 | 98.4 |
| 2 | 3.520 | dt | ^4^J_2,P_ = 3.1  ^3^J_2,3_ = 9.6 | 74.4 |
| 3 | 3.704 | t | ^3^J_3,4_ = 9.6 | 75.9 |
| 4 | 3.626 | m |  | 69.3 |
| 5 | 3.750 | m |  | 64.9 |

**Table S4: Crystallographic data collection and refinement statistics.** Numbers in parentheses indicate values for the highest resolution shell of data.

|  | EvdS6  (ligand-free state) | EvdS6  (ligand-bound state) |
| --- | --- | --- |
| **PDB ID** | 8SHH | 8SK0 |
| **SBGrid entry** | 1031 | 1030 |
| **Data collection** |  |  |
| Space group | P2_1_2_1_2_1_ | P2_1_2_1_2_1_ |
| Cell dimensions |  |  |
| *a, b, c* (Å) | 56.8, 77.5,146.9 | 59.9, 76.4, 148.5 |
| α, β, γ (°) | 90, 90, 90 | 90, 90, 90 |
| Resolution (Å) | 50-1.92 (1.95-1.92) | 50-1.51 (1.54-1.51) |
| R_sym_ | 0.072 (0.826) | 0.073 (0.604) |
| R_pim_ | 0.037 (0.453) | 0.040 (0.362) |
| I/σ | 27.5 (1.5) | 25.3 (2.1) |
| Completeness (%) | 99.8 (98.6) | 98.9 (99.7) |
| Redundancy | 4.7 (4.2) | 4.0 (3.4) |
| CC_1/2_ | 0.994 (0.675) | 0.996 (0.725) |
| **Refinement** |  |  |
| Resolution (Å) | 38.7-1.93 | 33.97-1.51 |
| No. reflections | 48902 | 106073 |
| R_work_/R_free_ | 0.190/0.233 | 0.1732/0.1988 |
| Ramachandran |  |  |
| Favored | 96.88 | 97.56 |
| Allowed | 2.65 | 2.44 |
| Outliers | 0.47 | 0.0 |
| No. atoms  Protein  Water | 4923  264 | 5088  830 |
| *B*-factors (mean)  Protein  Water | 36.90  41.13 | 17.35  29.40 |
| RMS deviations |  |  |
| bond lengths (Å)  bond angles (°) | 0.007  0.872 | 0.006  0.886 |

Single crystal was used for each structure. Values in parentheses are for highest-resolution shell.


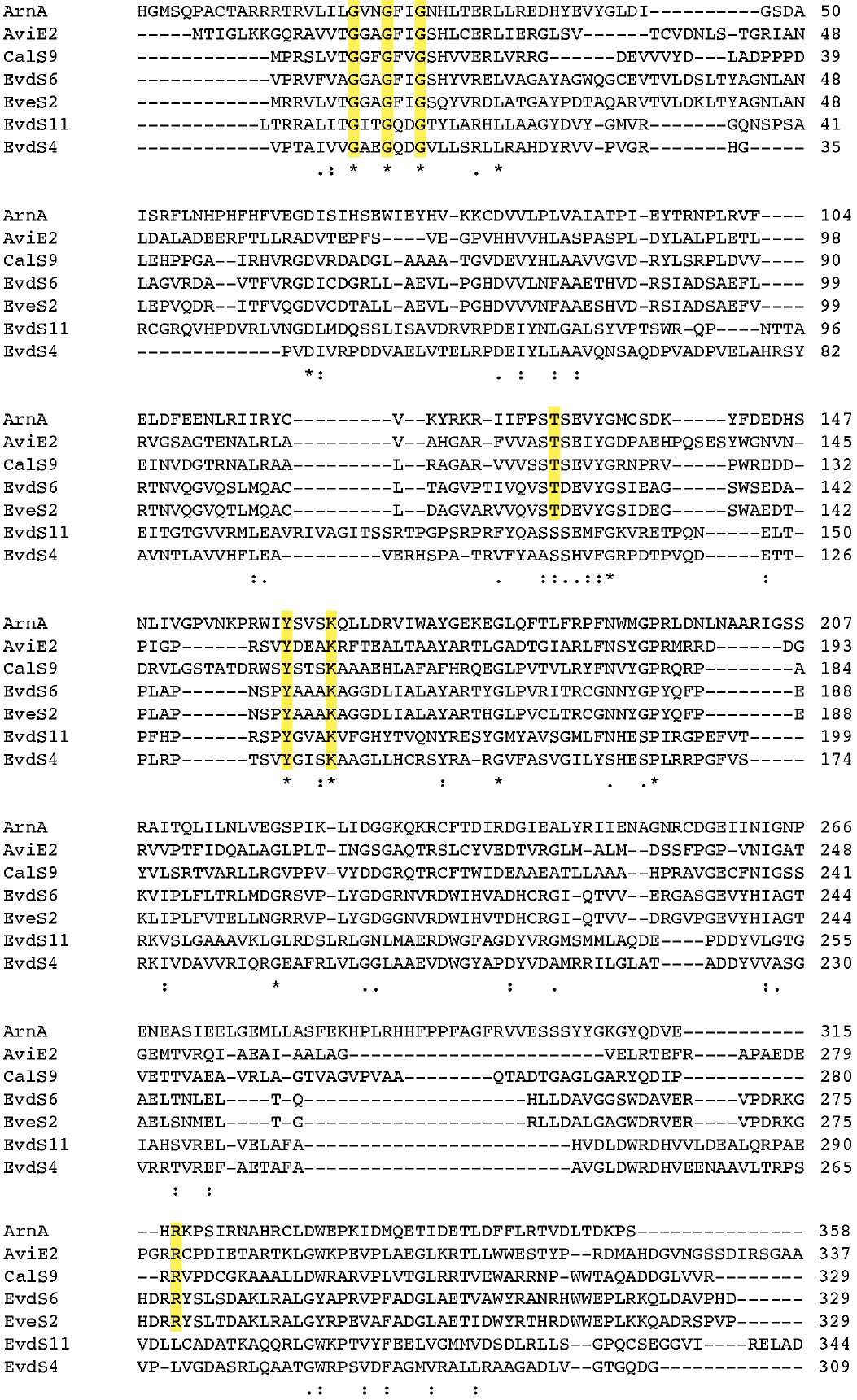


**Figure S1: Multiple Sequence Alignment of glucuronic acid decarboxylases.** Sequence identities used to identify EvdS6 as the glucuronic acid decarboxylase are highlighted in yellow. ArnA: *E. coli*, Lipid A. AviE2: *Streptomyces viridochromogenes* Tü57, avilamycin. CalS9: *Streptomyces* sp. KCTC 0041BP, calicheamicin. EveS2: *Micromonospora carbonacea* var. *africana*, everninomicin A, proposed decarboxylase.


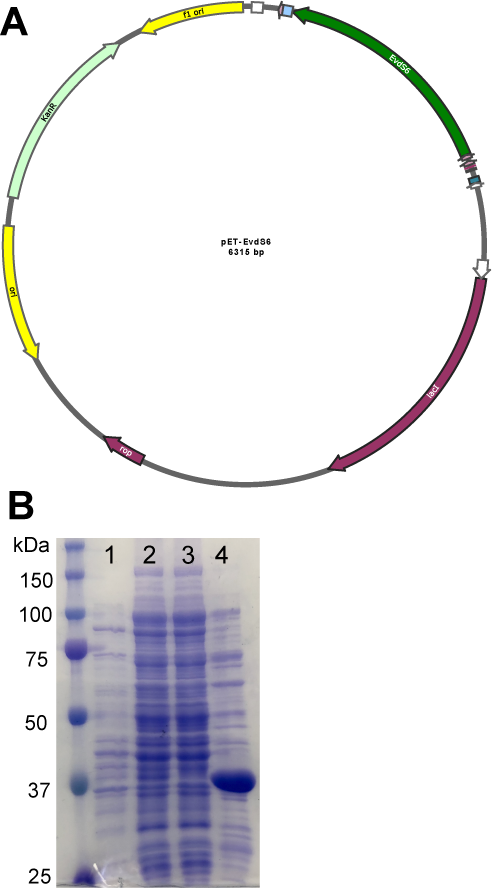


**Figure S2: EvdS6 plasmid map and purification. A.** pET-EvdS6 plasmid map used for expression. **B.** Purification of EvdS6. 1: Uninduced. 2: Crude lysate. 3: His-Trap flow thru. 4: Purified and concentrated EvdS6.

**Figure S3: MSMS Spectra of products. A.** MSMS spectra of the oxidized product, [M-H]^-^ 533. **B.** MSMS spectra of the reduced product, [M-H]^-^ 535. **C.** MSMS spectra of the reduced product hydrate, [M-H]^-^ 551. Common UMP fragment is m/z 323.


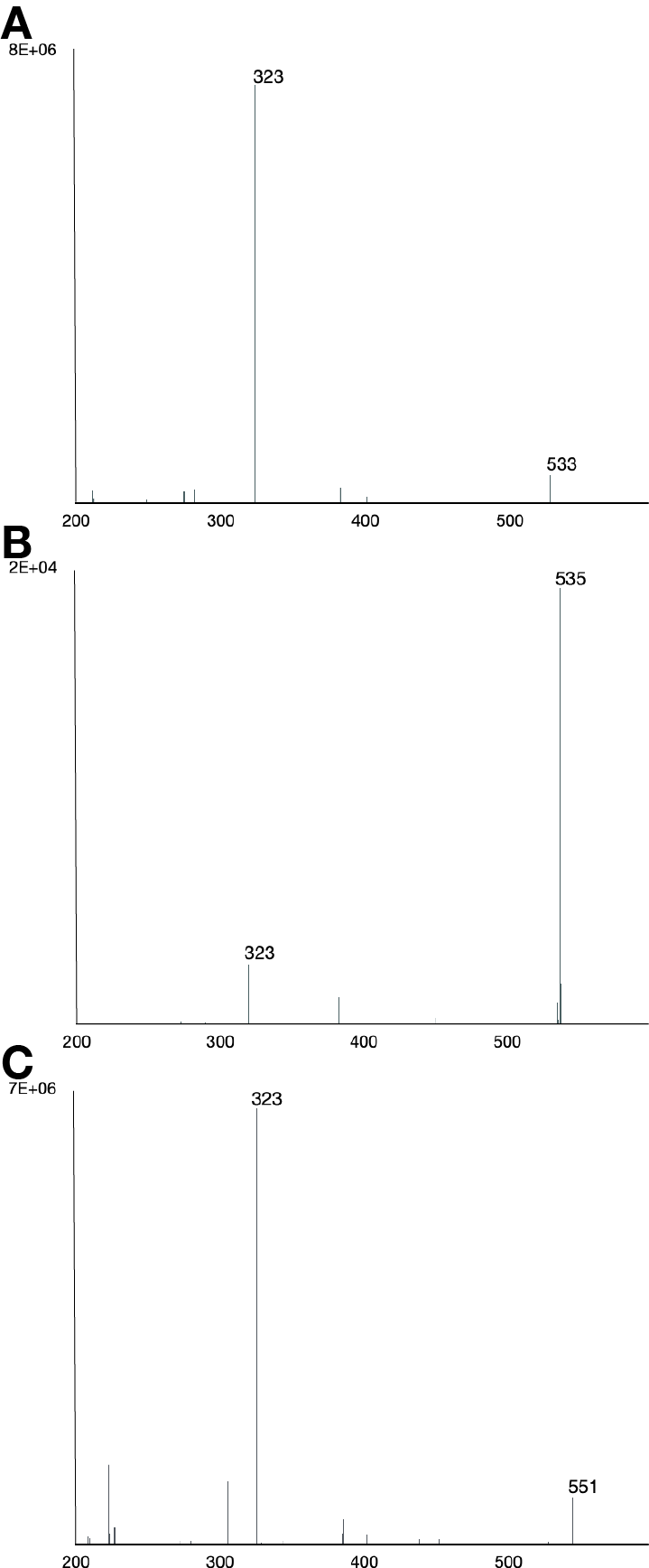


**Figure S4: Turnover optimization. A.** Cofactor screening. 50mM HEPES pH 8.5, 1mM UDP-GlcA, 1mM NAD^+^, 0.35mg/mL EvdS6. **B.** pH screening. 50mM HEPES, 1mM UDP-GlcA, 1mM NAD^+^, 0.38mg/mL EvdS6. **C.** Reaction timing. 50mM HEPES pH 8.5, 1mM UDP-GlcA, 0.1mM NAD^+^, 0.42mg/mL EvdS6. Oxidized product shown in green. Reduced product shown in blue.


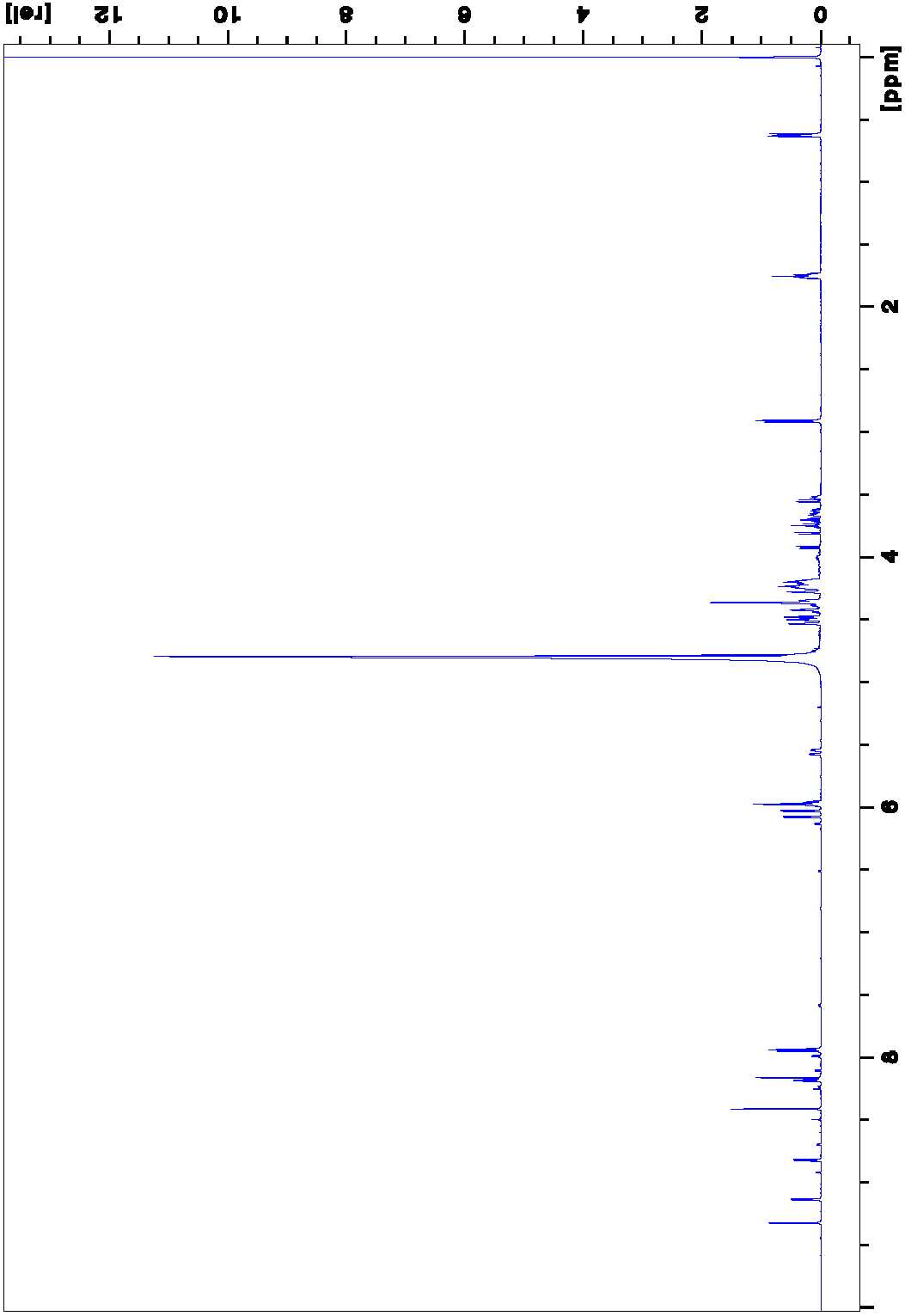


**Figure S5: ^1^H spectrum of the EvdS6 reaction.** 800 MHz water pre-saturation experiment utilizing a 5mm CPTCI probe, sweep width of 14 ppm, 2.93 sec acquisition, 2 sec recycle delay and 16 scans. The data was processed with 90 deg. shifted squared sinbell apodization and one-time zero filling. Spectrum was calibrated using DSS as a standard.


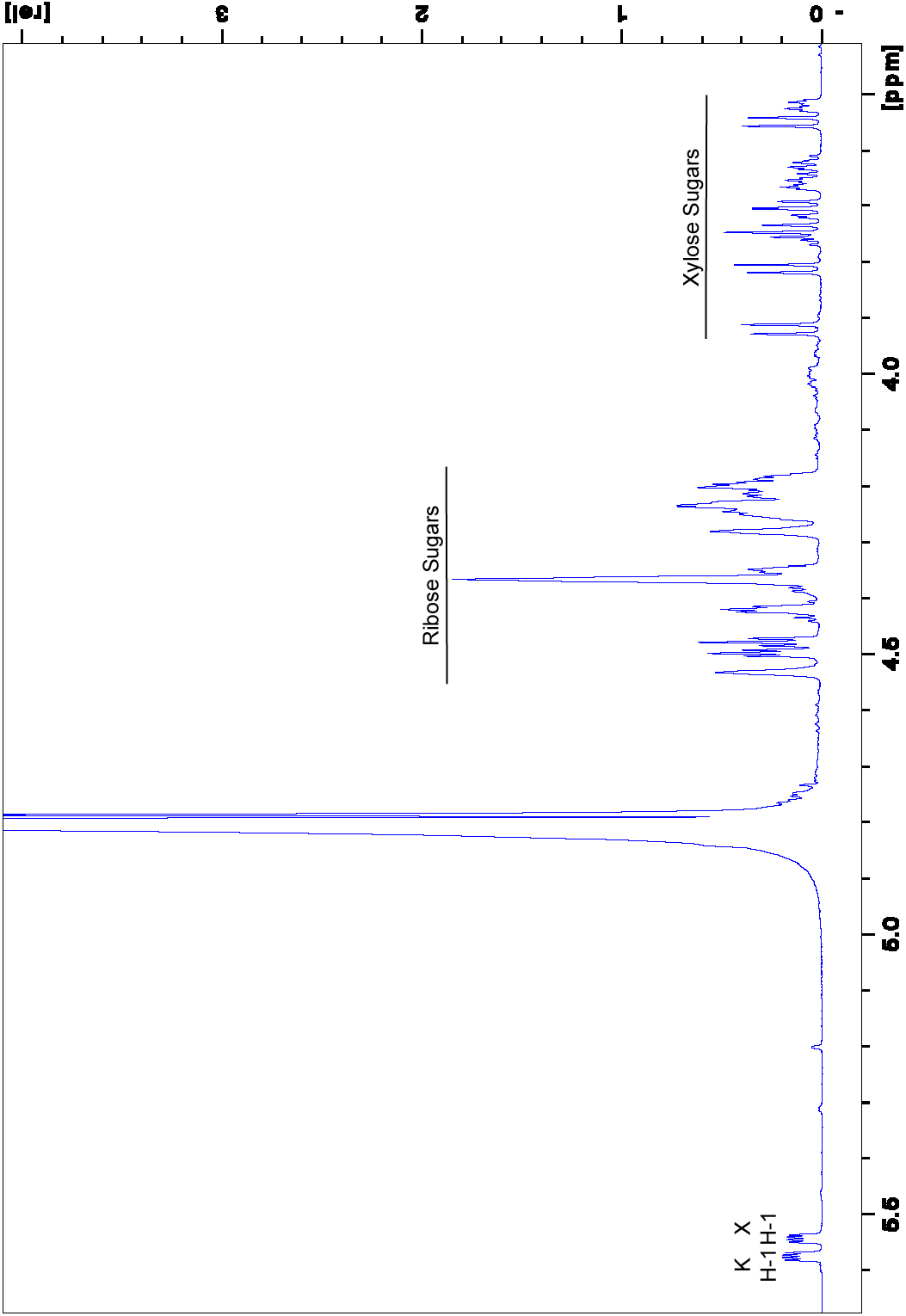


**Figure S6: Expansion of ^1^H spectrum of the EvdS6 reaction (Fig. S5).** Anomeric peaks of the two products are labeled. K: 4-keto-xylose and X: xylose. Ribose sugar peaks are from UDP of both products, NAD^+^ and NADH. Xylose sugar peaks refers to the product sugars.


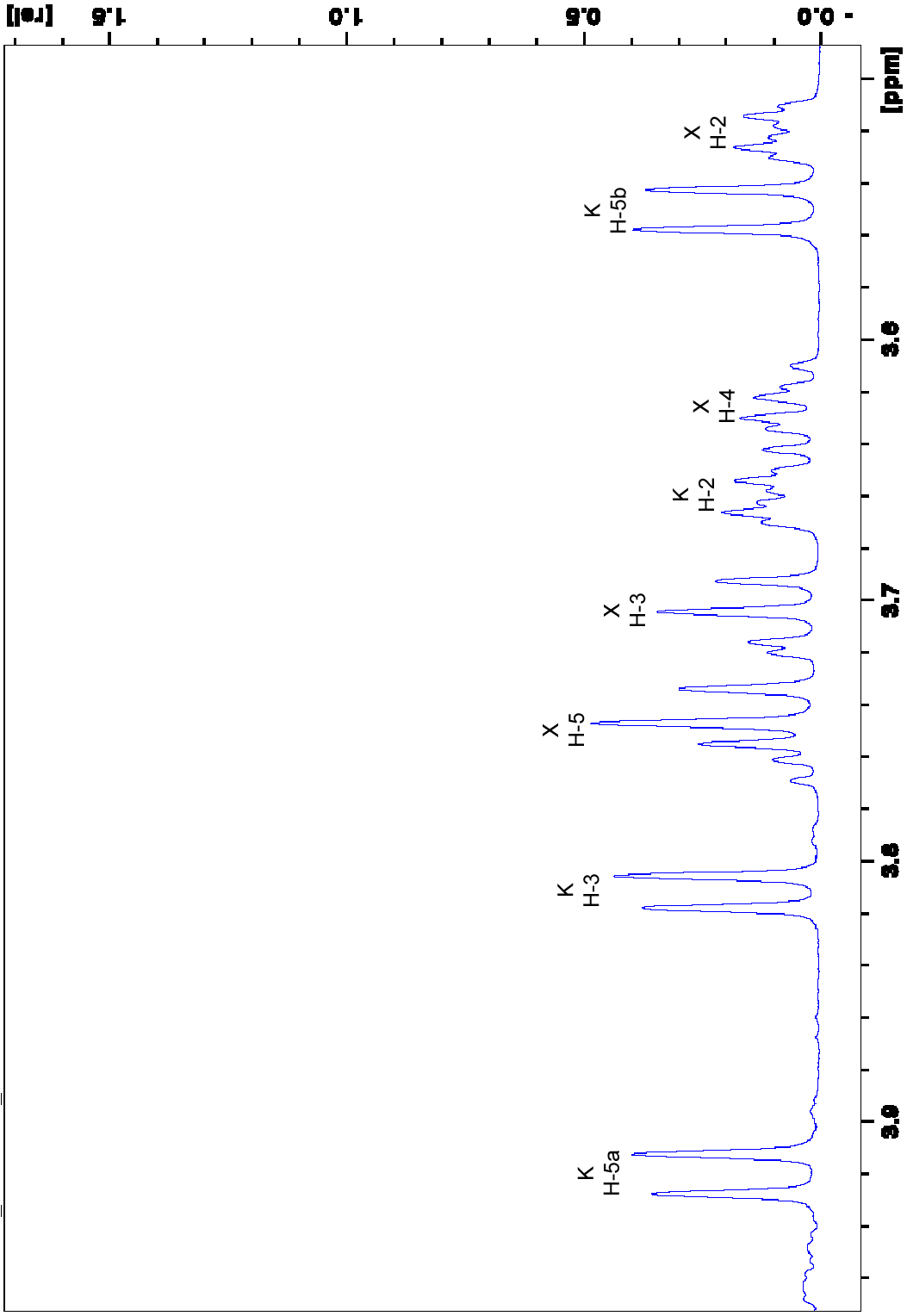


**Figure S7: Expansion of ^1^H spectrum of the EvdS6 reaction (Fig. S5).** Showing the xylose product peaks in greater detail. Peaks are labeled for the two products. K: 4-keto-xylose and X: Xylose.

**Figure S8: ^13^C{^1^H} spectrum of the EvdS6 reaction.** 800 MHz spectrometer utilizing a 5mm CPTCI probe, sweep width of 222 ppm with the offset at 105 ppm, 367 ms acquisition, 1.5 sec recycle delay, and 8192 scans. The data was processed with zero filling and exponential apodization of 2 Hz line broadening**.** Spectrum was calibrated using DSS as a standard.


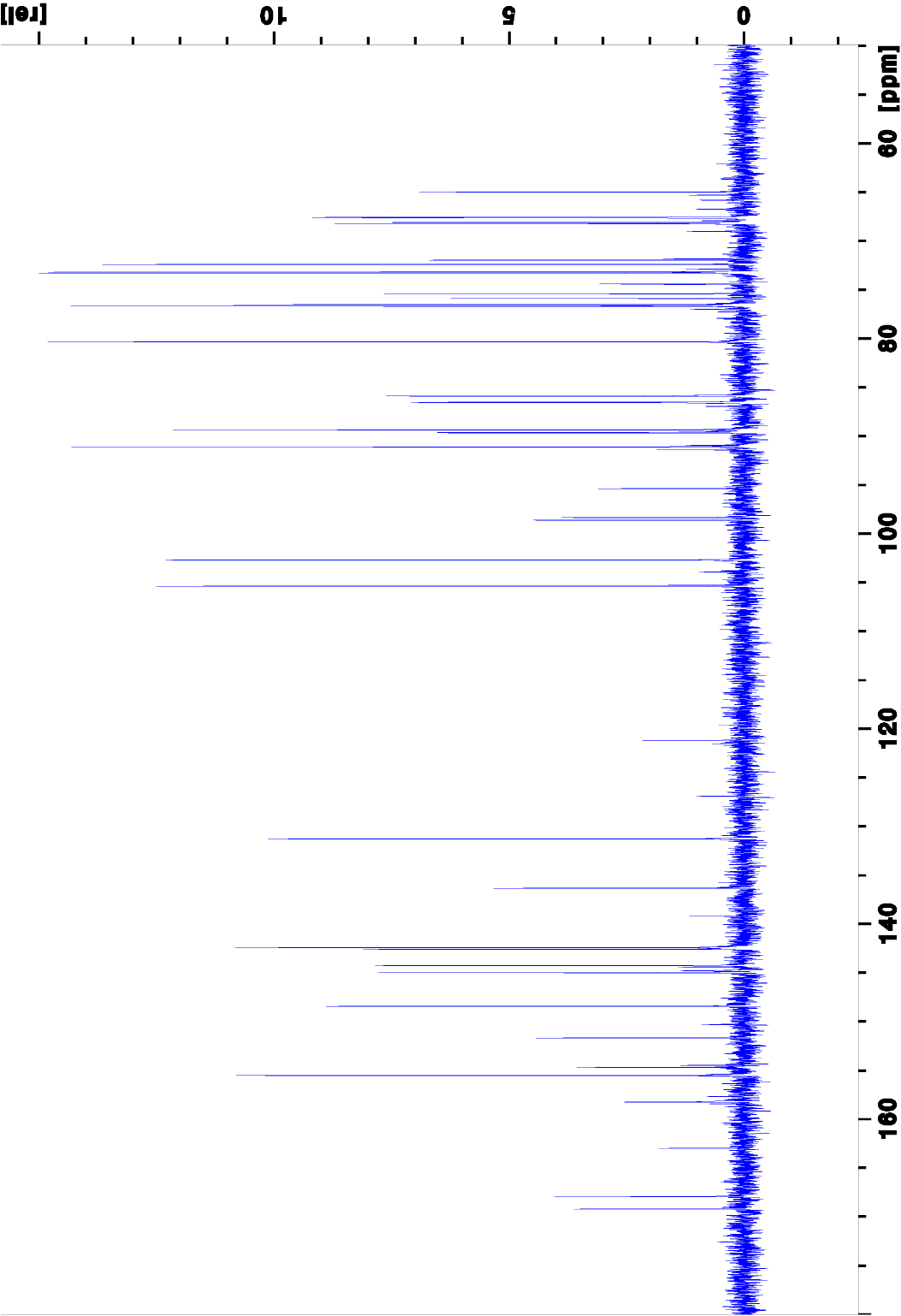

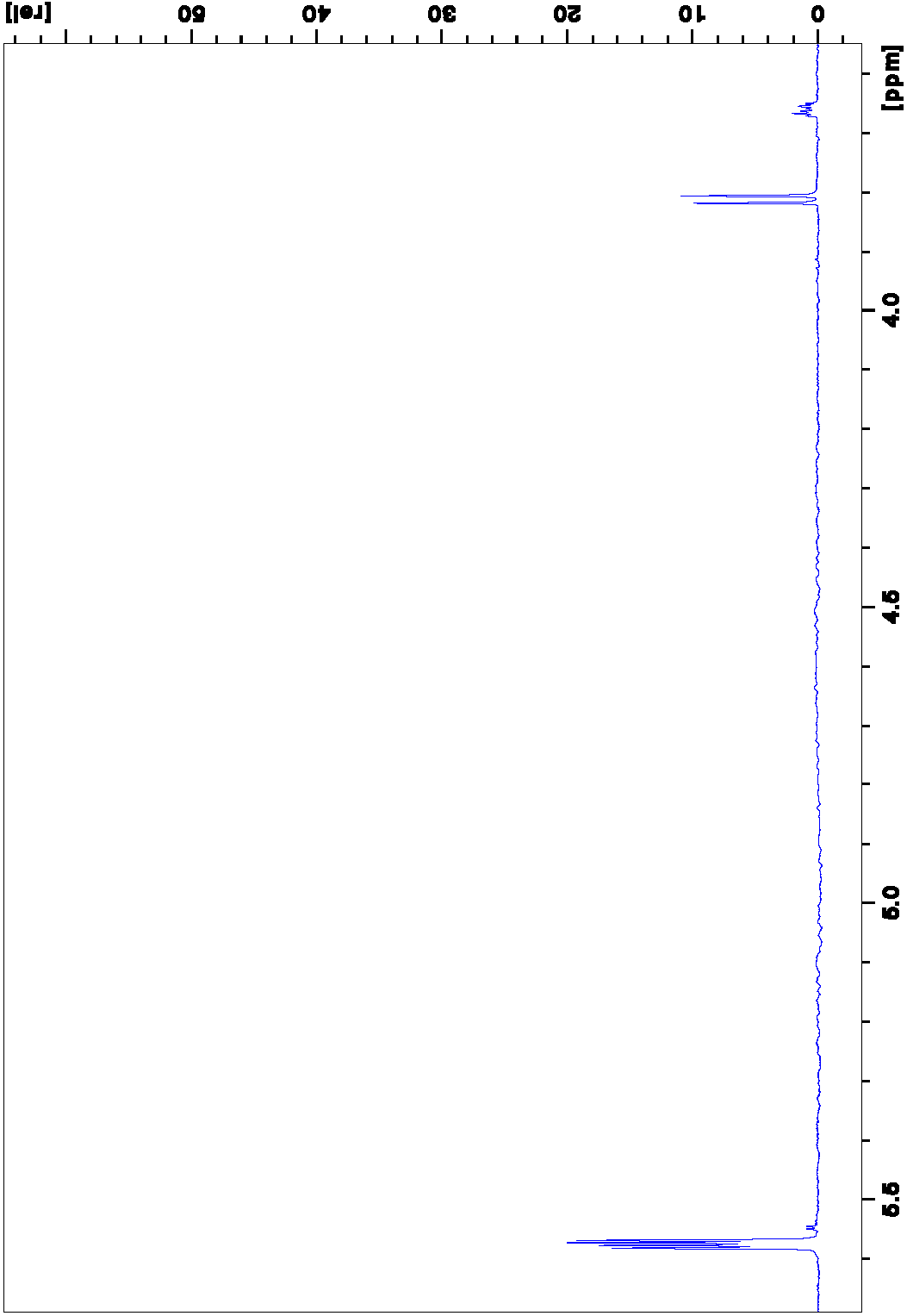


**Figure S9: Selective 1D TOCSY spectrum for the peak at 5.575 ppm.** Showing the spin system from H-1 to H-3 of the 4-keto-xylose product. Measuring conditions were as followed: 800 MHz water pre-saturation experiment utilizing a 5mm CPTCI probe, sweep width of 14 ppm, 2.93 sec acquisition, 2 sec recycle delay and 512 scans. Selectivity was achieved with a 73.5 ms Gaus180 pulse and the spinlock had a field of 10 kHz for 100ms The data was processed with an exponential apodization of 0.3 Hz line broadening, a gaussian (0.2 ppm) water filter, and one-time zero filling.


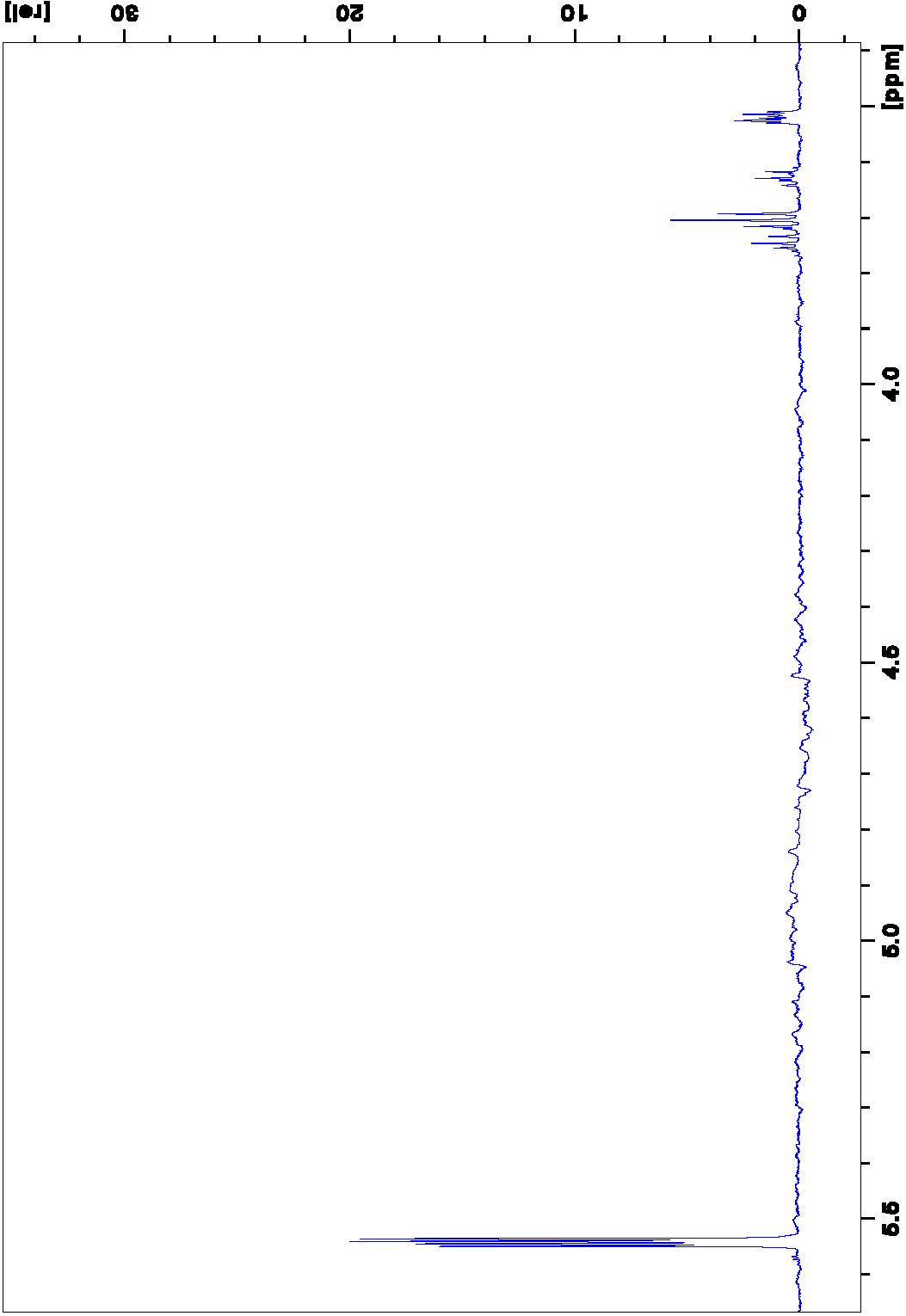


**Figure S10: Selective 1D TOCSY spectrum for the peak at 5.542.** Showing the spin system from H-1 to H-5 of the xylose product. Measuring conditions were as followed: 800 MHz water pre-saturation experiment utilizing a 5mm CPTCI probe, sweep width of 14 ppm, 2.93 sec acquisition, 2 sec recycle delay and 512 scans. Selectivity was achieved with a 72.9 ms Gaus180 pulse and the spinlock had a field of 10 kHz for 100ms. The data was processed with an exponential apodization of 0.3 Hz line broadening, a gaussian (0.15 ppm) water filter, and one-time zero filling.


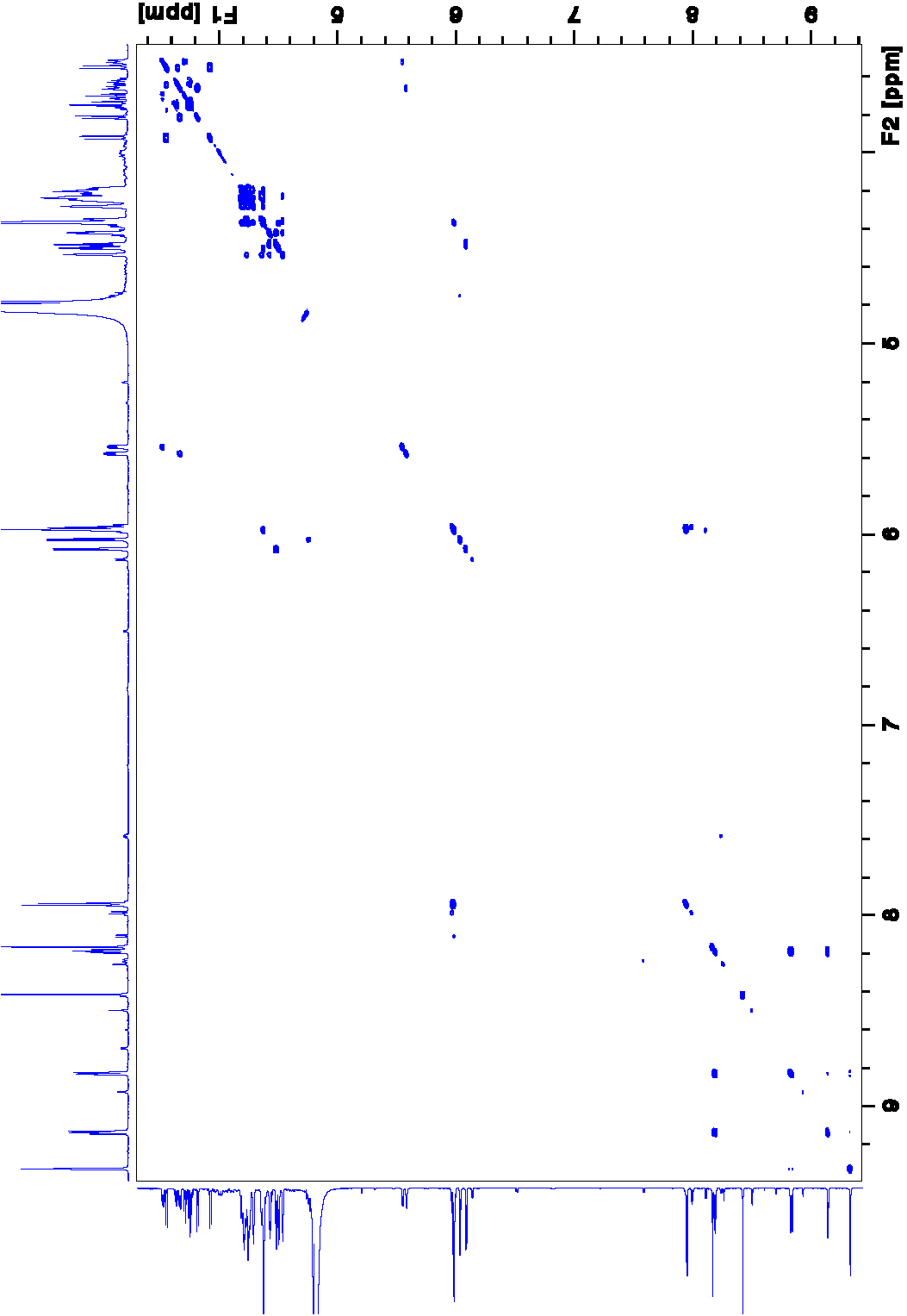


**Figure S11: COSY spectrum of the EvdS6 reaction.** Spectrum was calibrated using the ^1^H spectrum with DSS. Measuring conditions were as followed: 800 MHz water pre-saturation, magnitude COSY experiment utilizing a 5mm CPTCI probe, sweep width of 10.52 ppm, 121.6 and 118.8 ms acquisition in the direct and indirect dimension, respectively, 2 sec recycle delay and 16 scans. The data was processed with a squared sinbell apodization, 2048 points in both dimensions, and a gaussian (0.1 ppm) water filter.


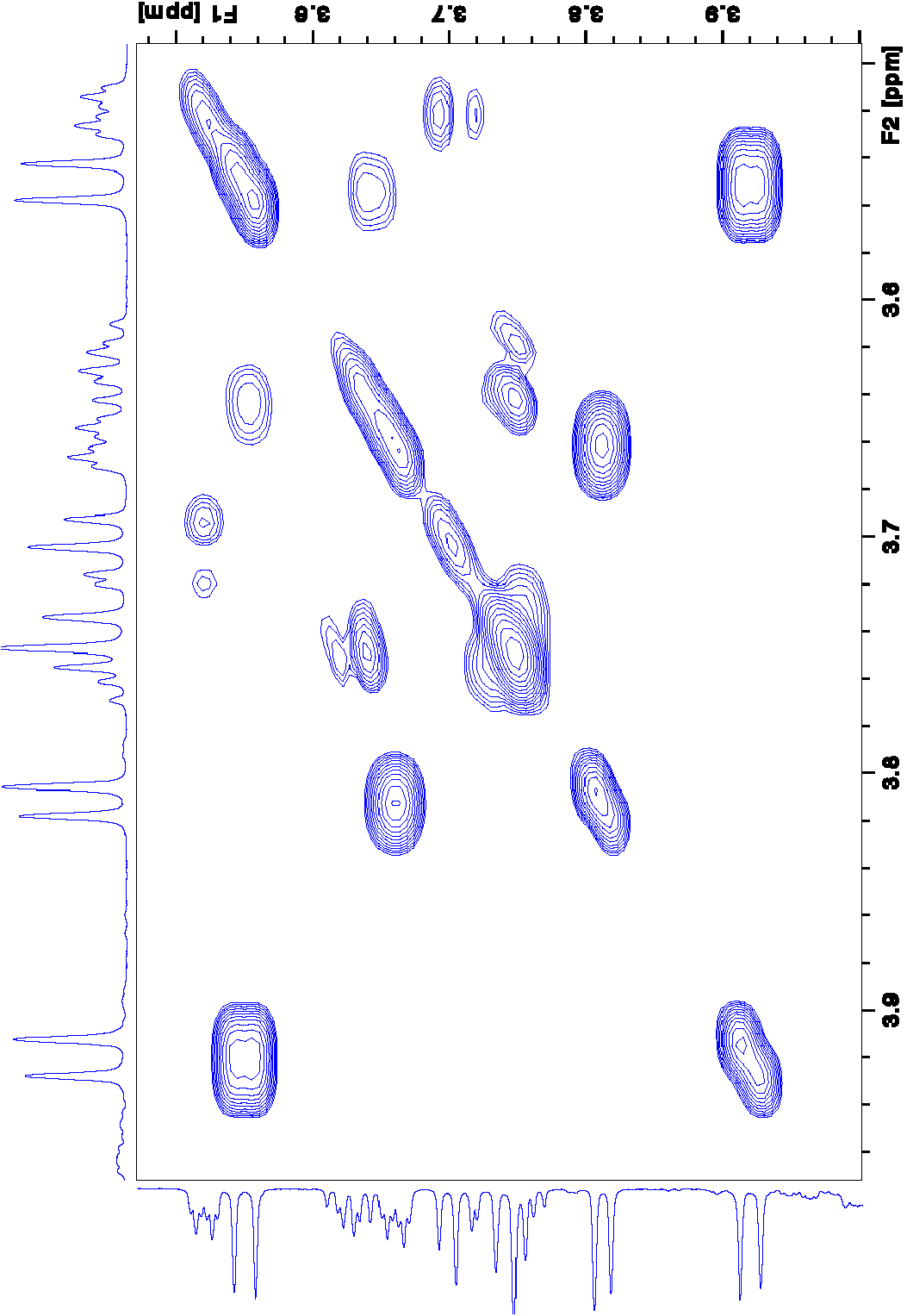


**Figure S12: Expansion of the COSY spectrum of the EvdS6 reaction (Fig. S11).** Showing the reaction product peaks in greater detail.

**Figure S13: HSQC spectrum of the EvdS6 reaction.** Spectrum was calibrated using the ^1^H and ^13^C spectra with DSS. Measuring conditions were as followed: 800 MHz E/A – TPPI HSQC experiment with multiplicity editing, J_C-H_ = 150 Hz, utilizing a 5mm CPTCI probe, spectrum center at 4.7 and 105 ppm, 105.5 and 38.3 ms acquisition and 2048 by 256 points each in the direct and indirect dimension, respectively, 1.5 sec recycle delay and 32 scans. The data was processed with a 90 deg. shifted squared sinbell apodization, 2048 points in both dimensions, and a polynomial water filter.


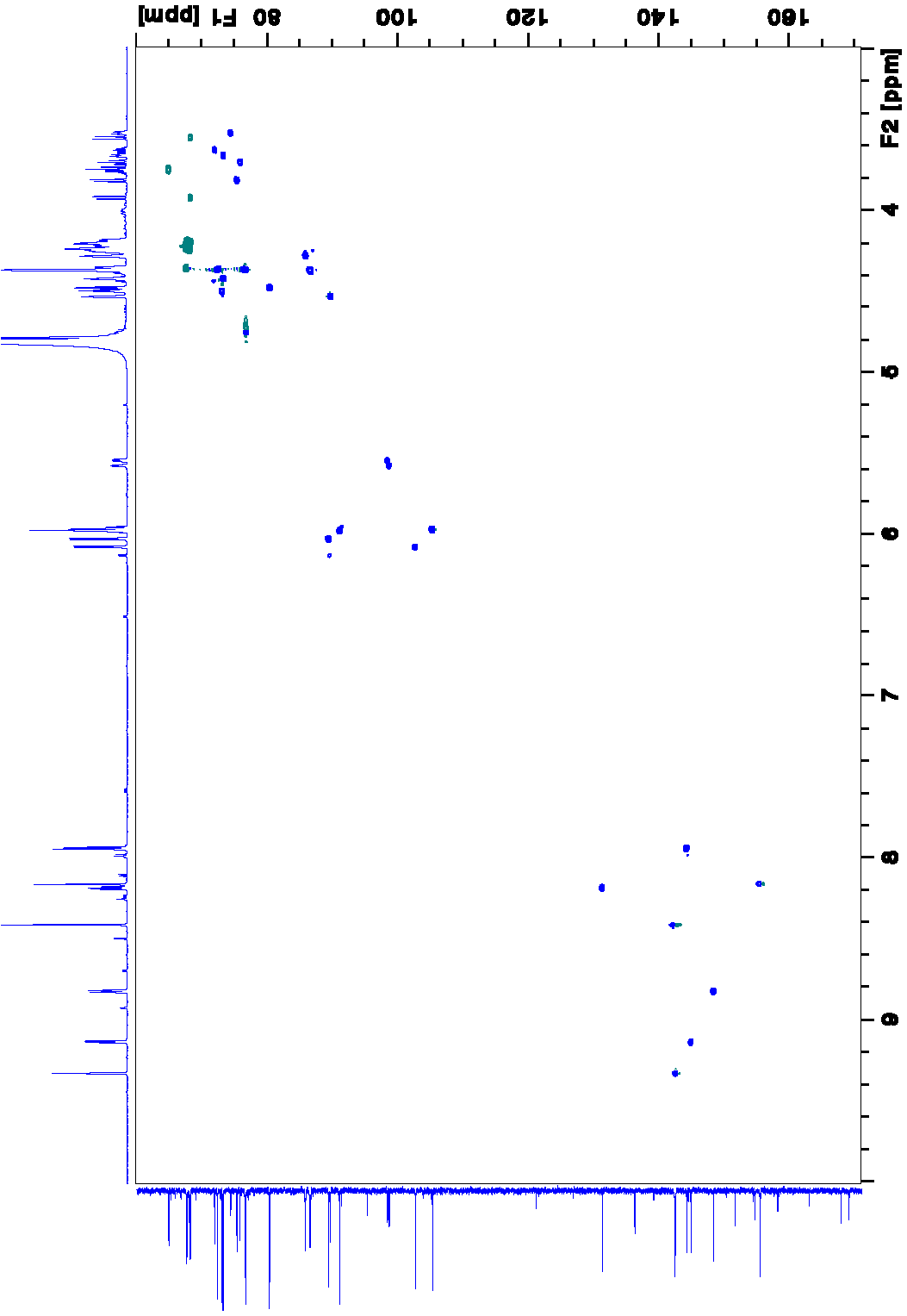


**Figure S14: Expansion of the HSQC spectrum of the EvdS6 reaction (Fig. S13).** Showing the reaction product peaks in greater detail.


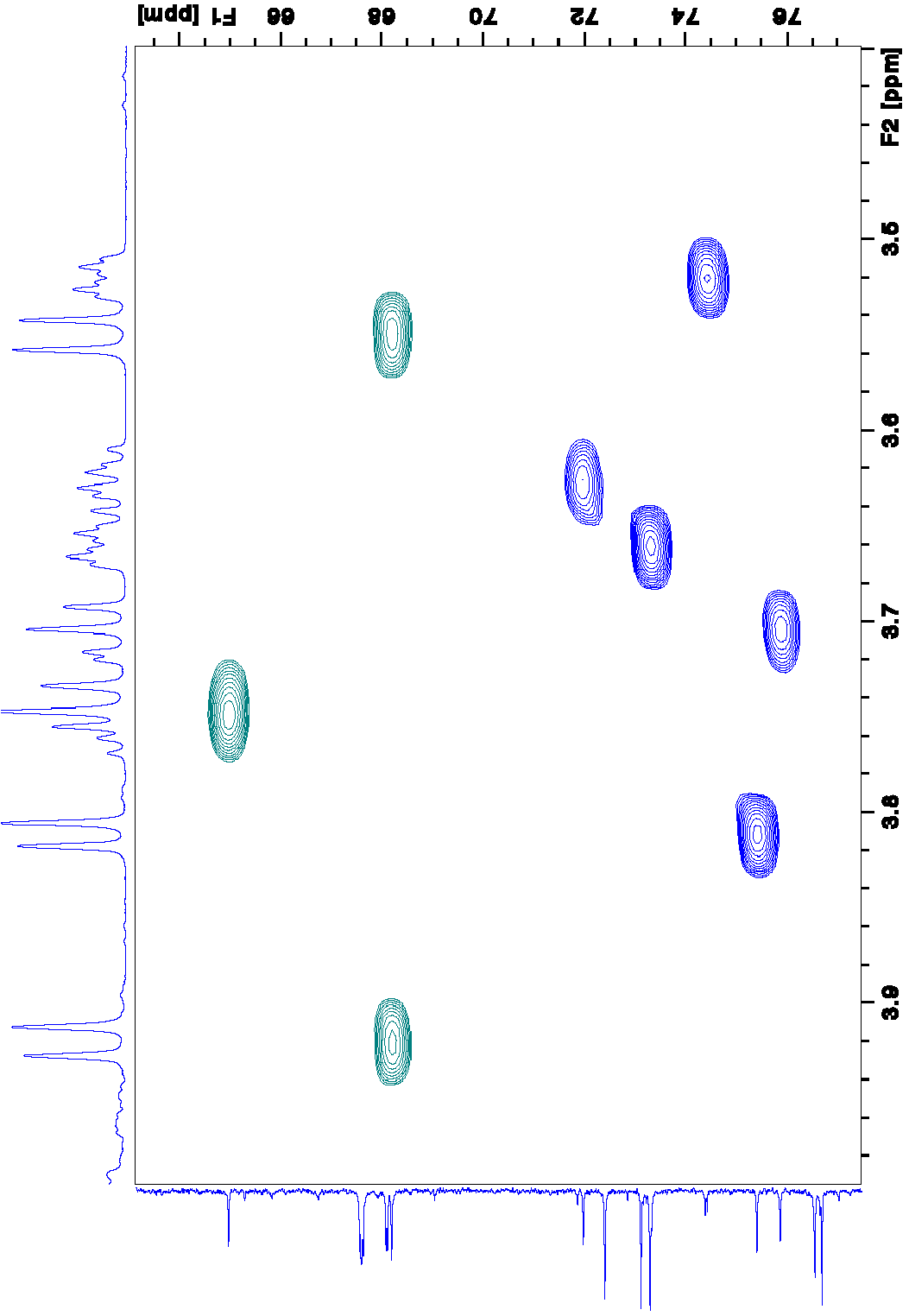


**Figure S15: HMBC spectrum of the EvdS6 reaction.** Spectrum was calibrated using the ^1^H and ^13^C spectra with DSS. Measuring conditions were as followed: 800 MHz presaturation, magnitude HMBC experiment with low-pass J-filter utilizing a 5mm CPTCI probe, spectrum center at 4.7 and 135 ppm, 60.8 and 29.2 ms acquisition, and 1024 by 512 points each in the direct and indirect dimension, respectively, 1.5 sec recycle delay and 128 scans. Delays were optimized for J_C-H_ = 150 Hz and 8 Hz. The data was processed with a 90 deg. shifted squared sinbell, 2048 points in both dimensions, and a polynomial water filter.


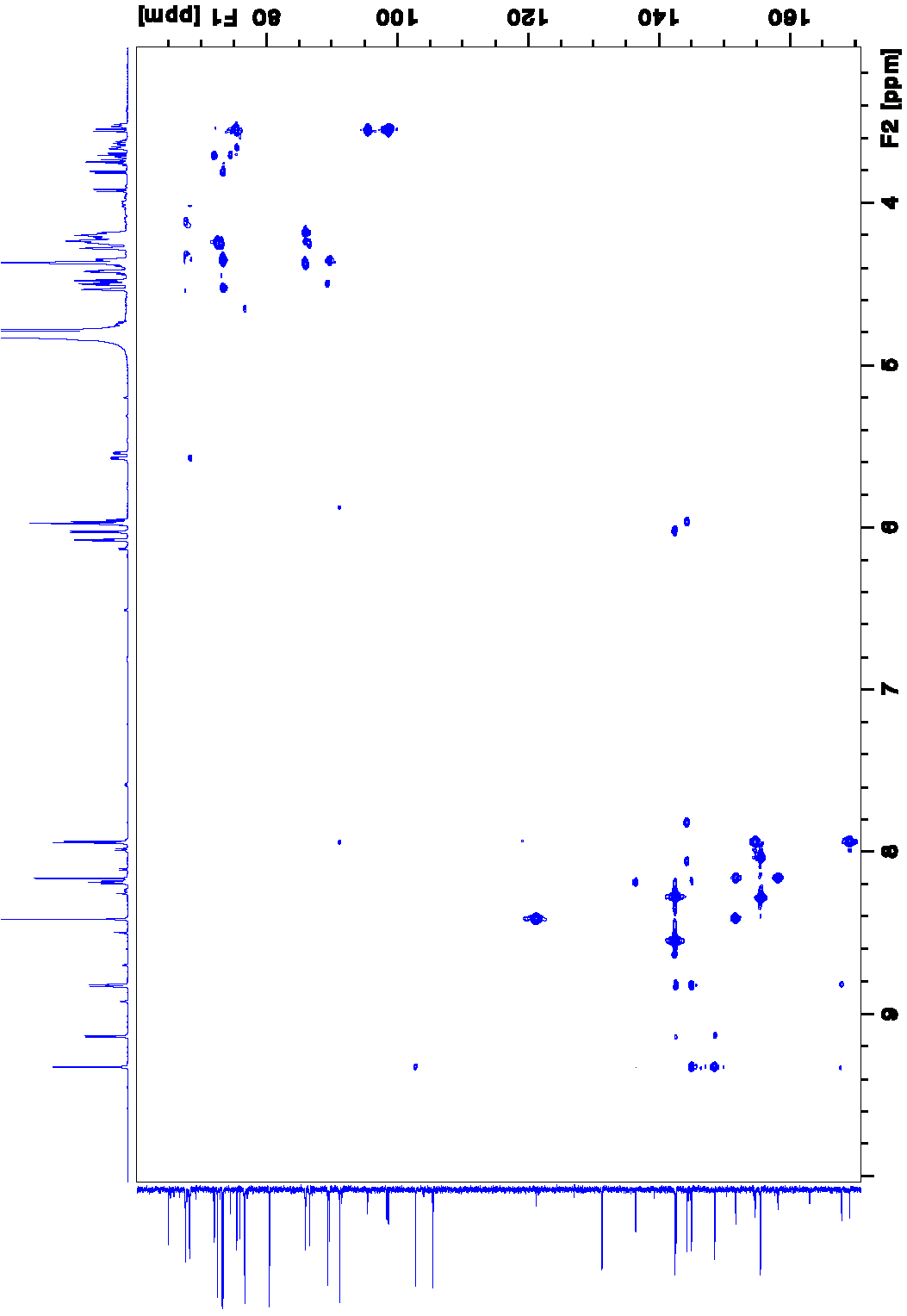


**Figure S16: Expansion of the HMBC spectrum of the EvdS6 reaction (Fig. S15).** Showing the reaction product peaks in greater detail.


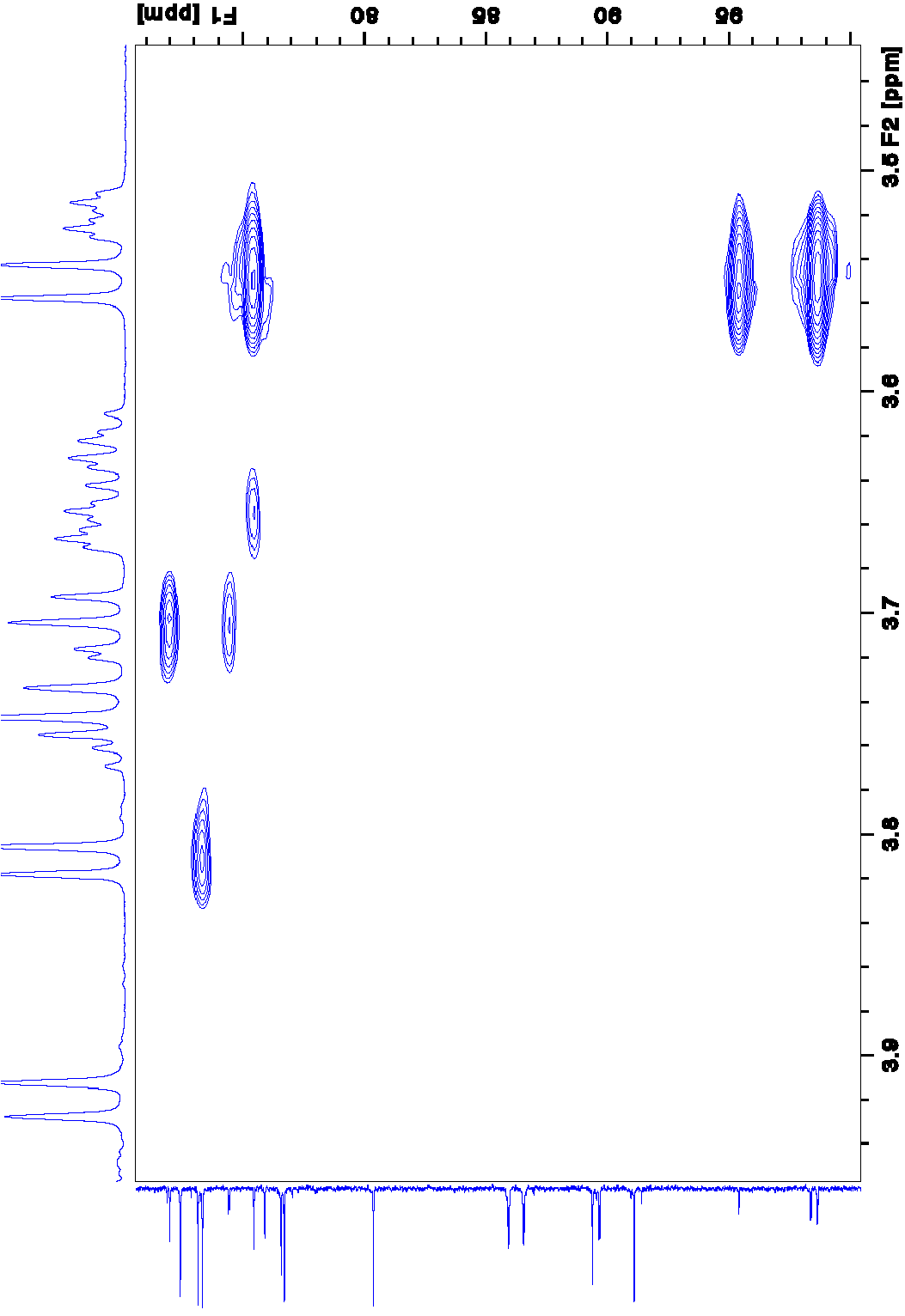

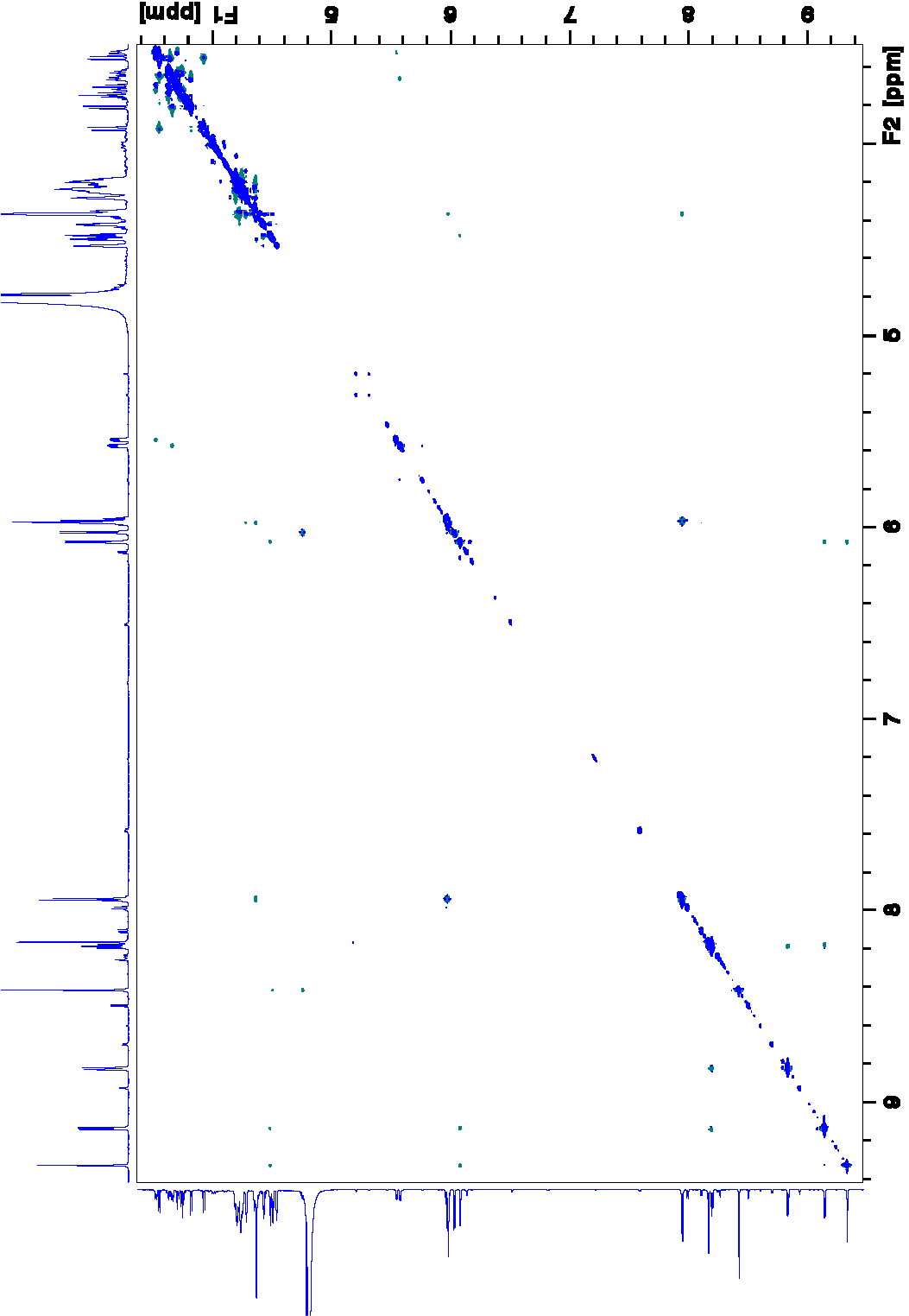


**Figure S17: NOESY spectrum of the EvdS6 reaction.** Spectrum was calibrated using the ^1^H spectrum with DSS. Measuring conditions were as followed: 800 MHz States-TPPI NOESY experiment with excitations sculpting for water suppression utilizing a 5mm CPTCI probe, sweep width of 10.51 ppm, 121.8 and 119.0 ms acquisition in the direct and indirect dimension, respectively, 500 ms NOE mixing time, 2 sec recycle delay and 16 scans. The data was processed with a 90 deg. shifted squared sinbell apodization, 2048 points in both dimensions, and a gaussian (0.05 ppm) water filter.


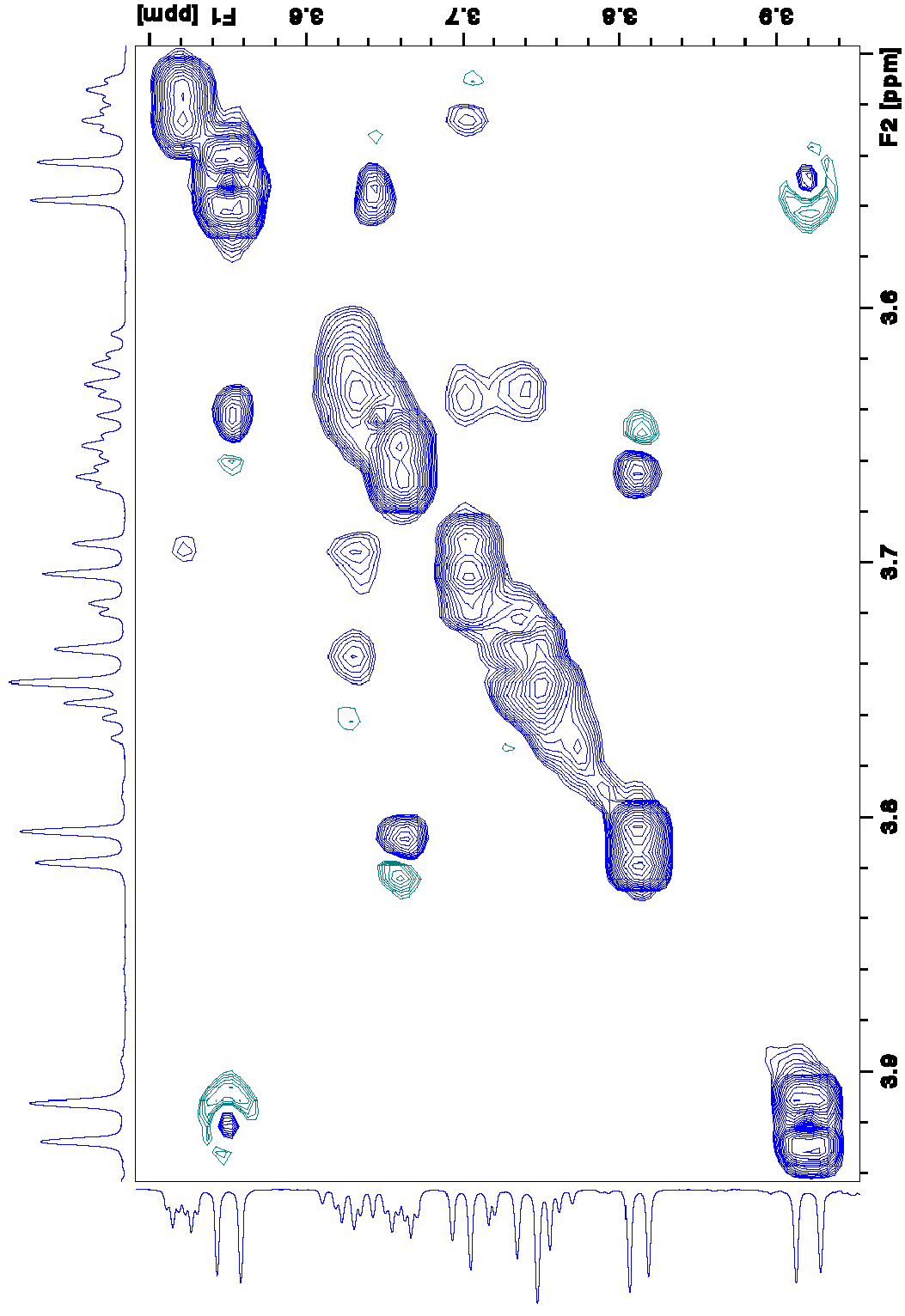


**Figure S18: Expansion of the NOESY spectrum of the EvdS6 reaction (Fig. S17).** Showing the reaction product peaks in greater detail.


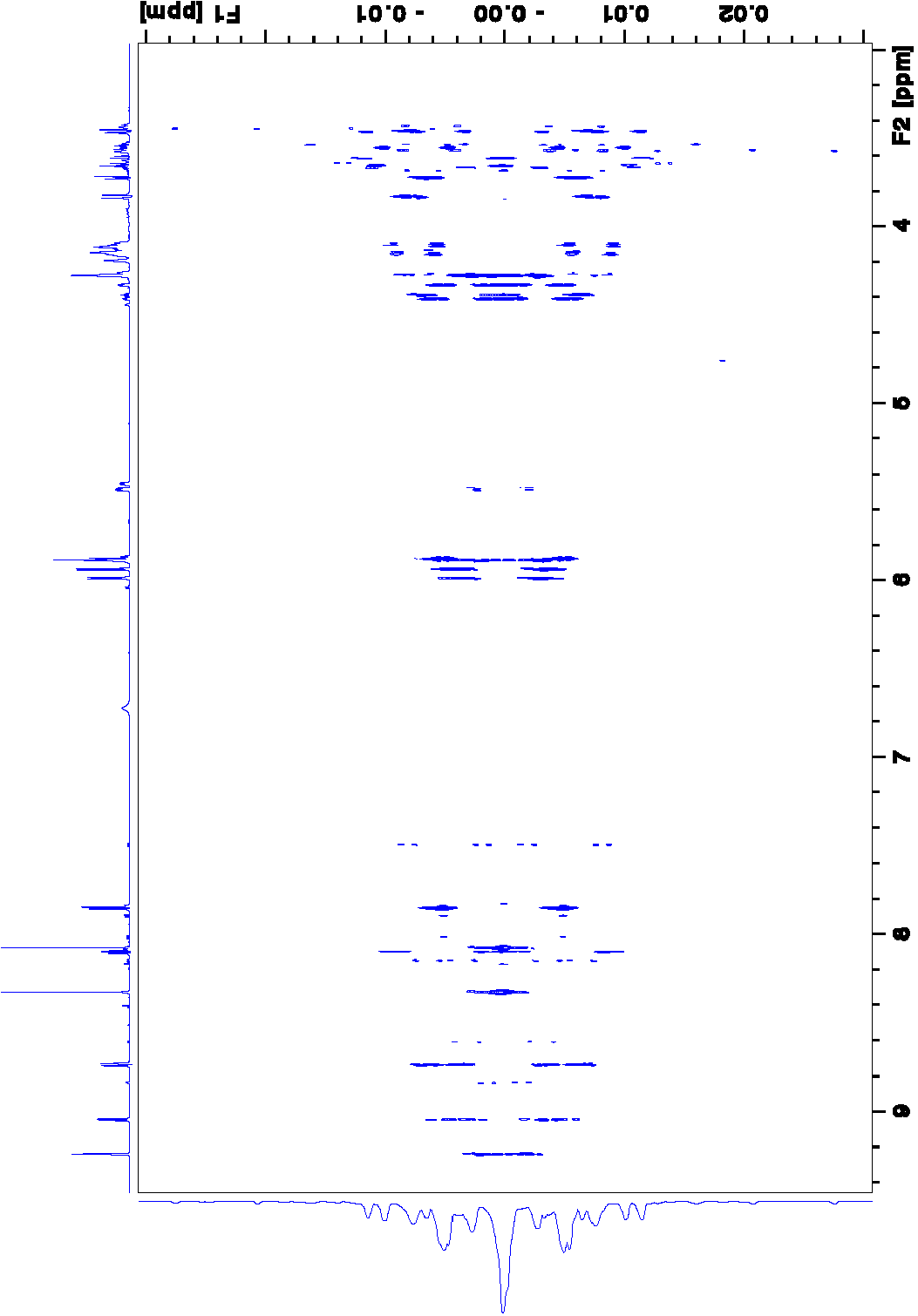


**Figure S19: Homonuclear J-resolved spectrum of the EvdS6 reaction.** Measuring conditions were as followed: 800 MHz pre-saturation, magnitude experiment utilizing a 5mm CPTCI probe, sweep width of 10.02 ppm and 50 Hz, 1.022 and 2.56 sec acquisition in the direct and indirect dimension, respectively, 2 sec recycle delay and 16 scans. The data was processed with a sinbell apodization, 2048 points in both dimensions, and a gaussian (0.05 ppm) water filter.

**Figure S20**: **The structure of EvdS6 in the ligand-free state.** Structure of EvdS6 dimer in ligand-free state is shown in ribbon (cyan) representation. The co-factor NAD^+^ is shown as a stick representation in yellow.


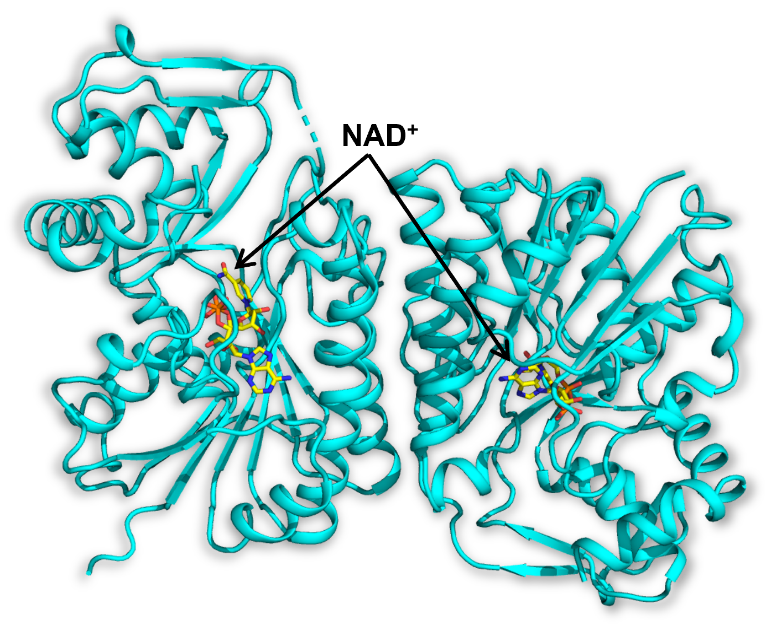


**Figure S21:** **Interaction interface between the EvdS6 dimer.** **A.** Structure of EvdS6 dimer with co-factor NAD^+^ and TDP is shown in ribbon (cyan) representation. The co-factor NAD^+^ is shown as a stick representation with carbons yellow, oxygens red, nitrogens blue, and phosphorous orange. The ligand TDP is also shown as a stick representation with carbons magenta, oxygens red, nitrogens blue, and phosphorous orange. The interaction interface of the dimer is shown in surface representation. **B.** An alternative view of EvdS6 dimer rotated 70° around x-axis as shown in **A**. The interaction interface of the dimer highlighted and is shown in ribbon and surface representation.


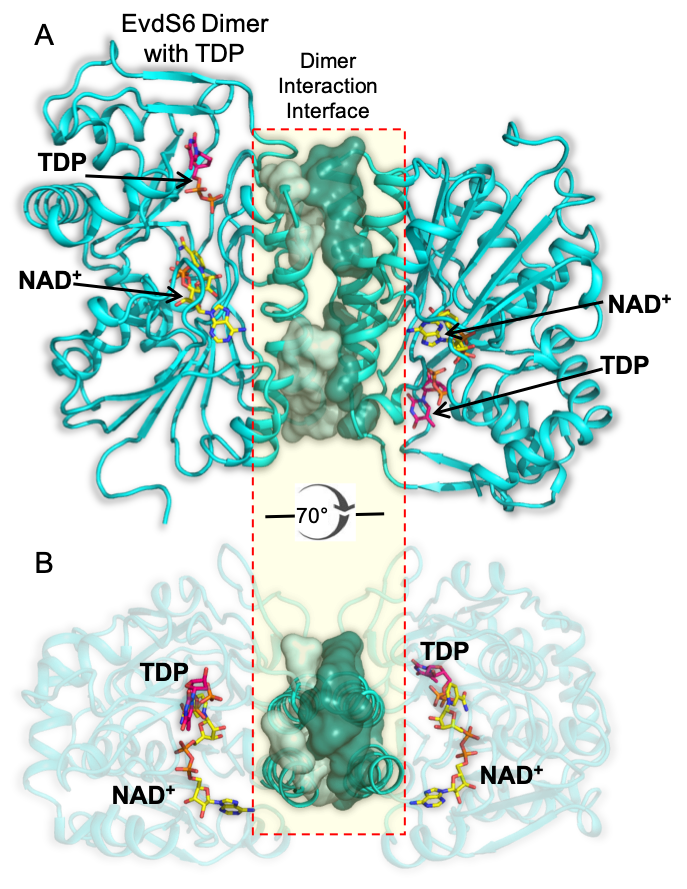


**A**

**B**

**Figure S22:** **Comparison of flexibility of EvdS6 crystal structures.** **A.** Ribbon representation of EvdS6 dimer in TDP bound state. The width and color of the ribbon represent flexibility with thin ribbon and blue color represent higher stability and think ribbon and color gradient from green to red represent higher flexibility. The unstructured active site loop of both subunits is represented in rectangle. This region show some flexibility when TDP is bound to the active site of EvdS6. **B.** Ribbon representation of EvdS6 dimer in ligand free state. The unstructured active site loop in this state show higher flexibility as compares to ligand bound state of EvdS6 in **A**. In fact, some residues of this loop become highly flexible in ligand free state as they cannot be traced with interpretable electron density.


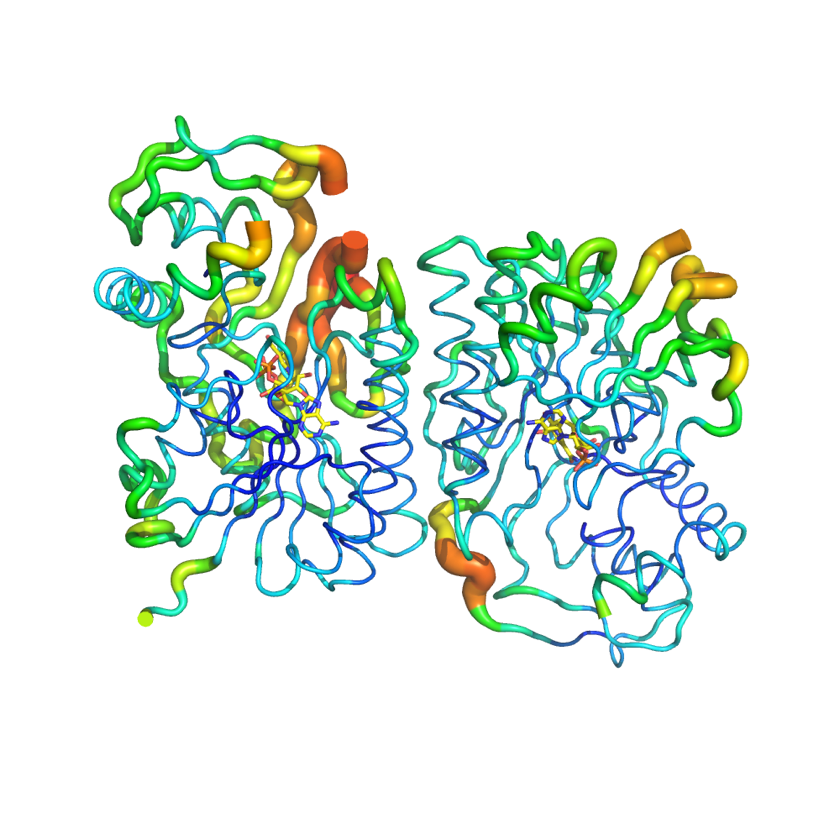


EvdS6 active site

in ligand-free state

Unstructured active site loop


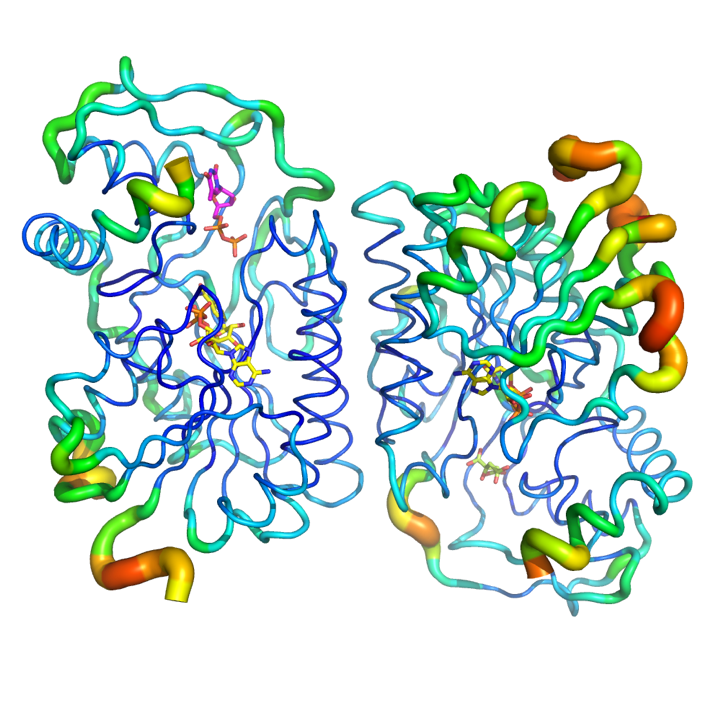


EvdS6 active site

in ligand-bound state

Unstructured active site loop

**A**

**B**


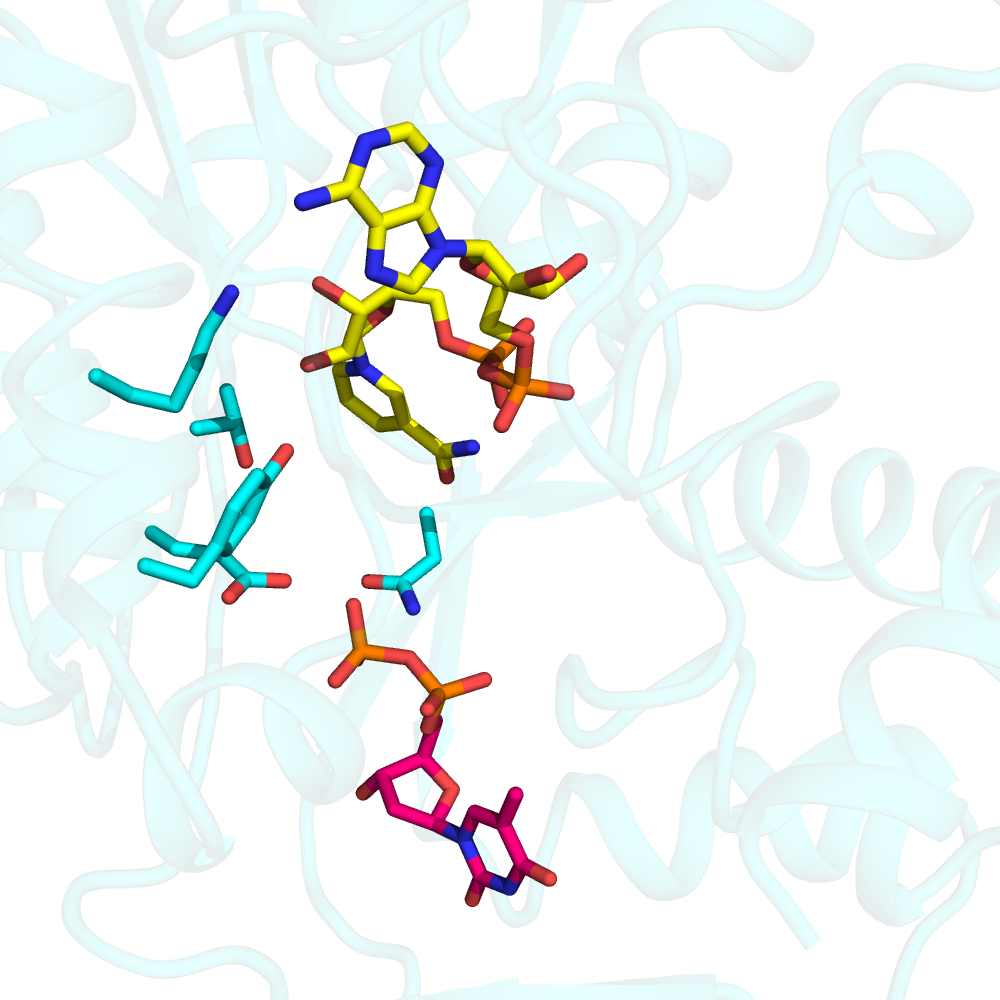


**NAD^+^**

T126

K154

E128

N179

Y150

**TDP**


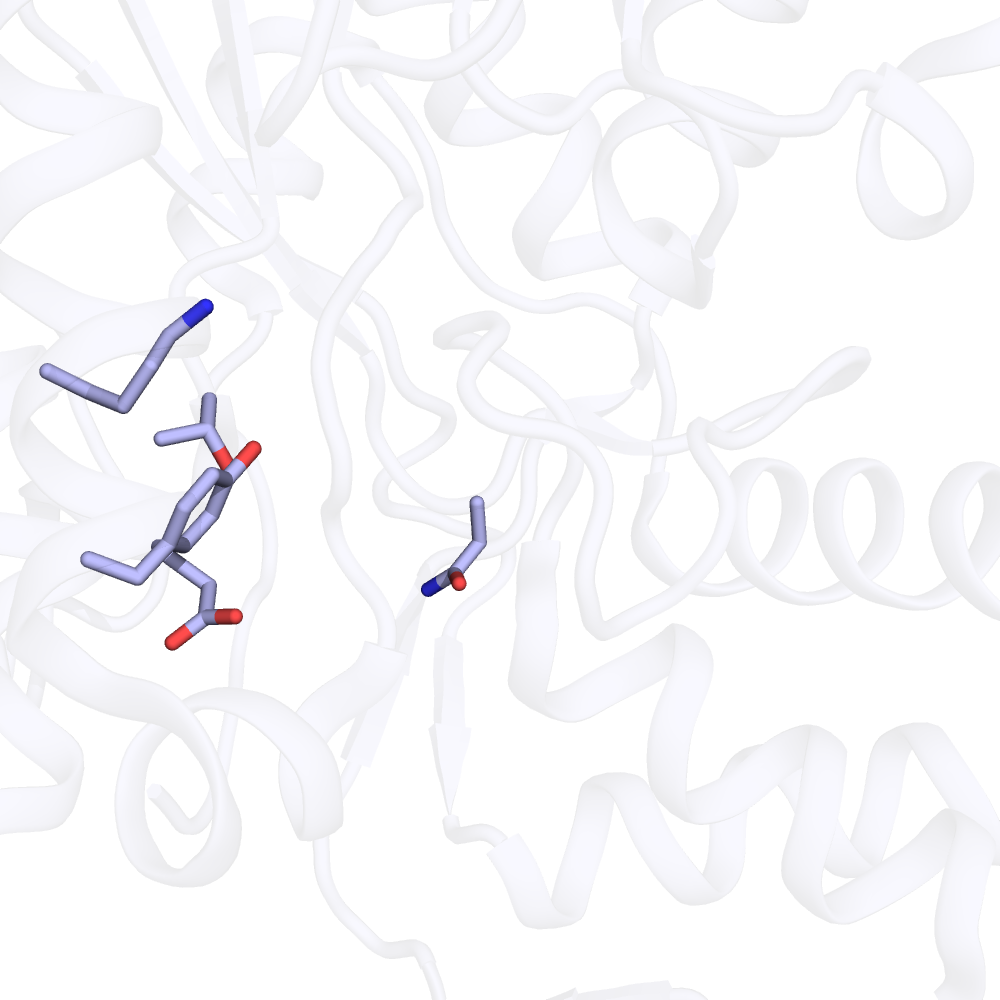


T432

K467

E434

N492

Y463


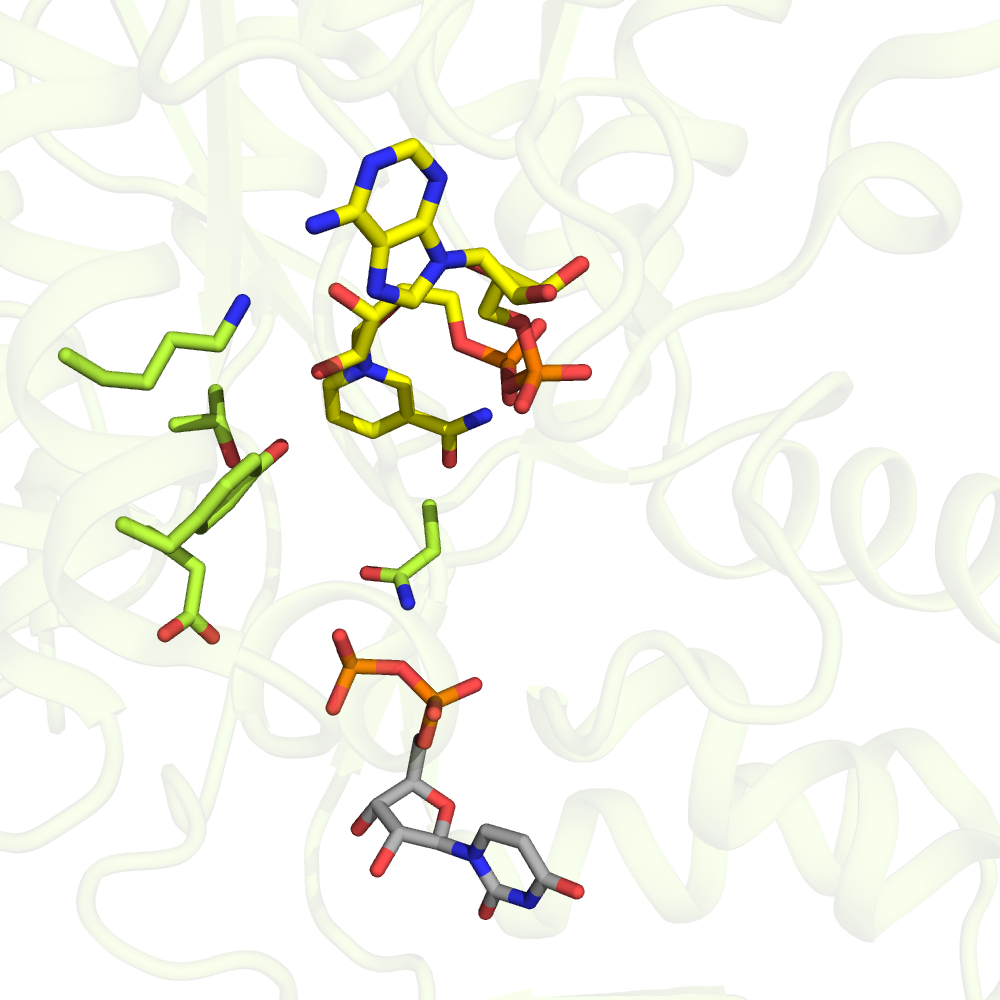


**NAD^+^**

**UDP**

T118

K151

E120

N176

Y147

**Figure S23: Active site residues.** The active site residues of EvdS6 and two other NDP-glucuronic acid decarboxylases are compared. The catalytic triad Y150, K154, and T126 (EvdS6 numbering) are shown along with E128 and N179, which have significant sequence identity across the subclass of enzymes. These residues are believed to define the sugar binding site. 2BLL is the *E. coli* protein ArnA involved in Lipid A biosynthesis. 2B69 is the human protein hUxs1 that produces xylose. NAD^+^ is shown in yellow, TDP is shown in magenta, and UDP is shown in grey.

**EvdS6**

**2BLL**

**2B69**


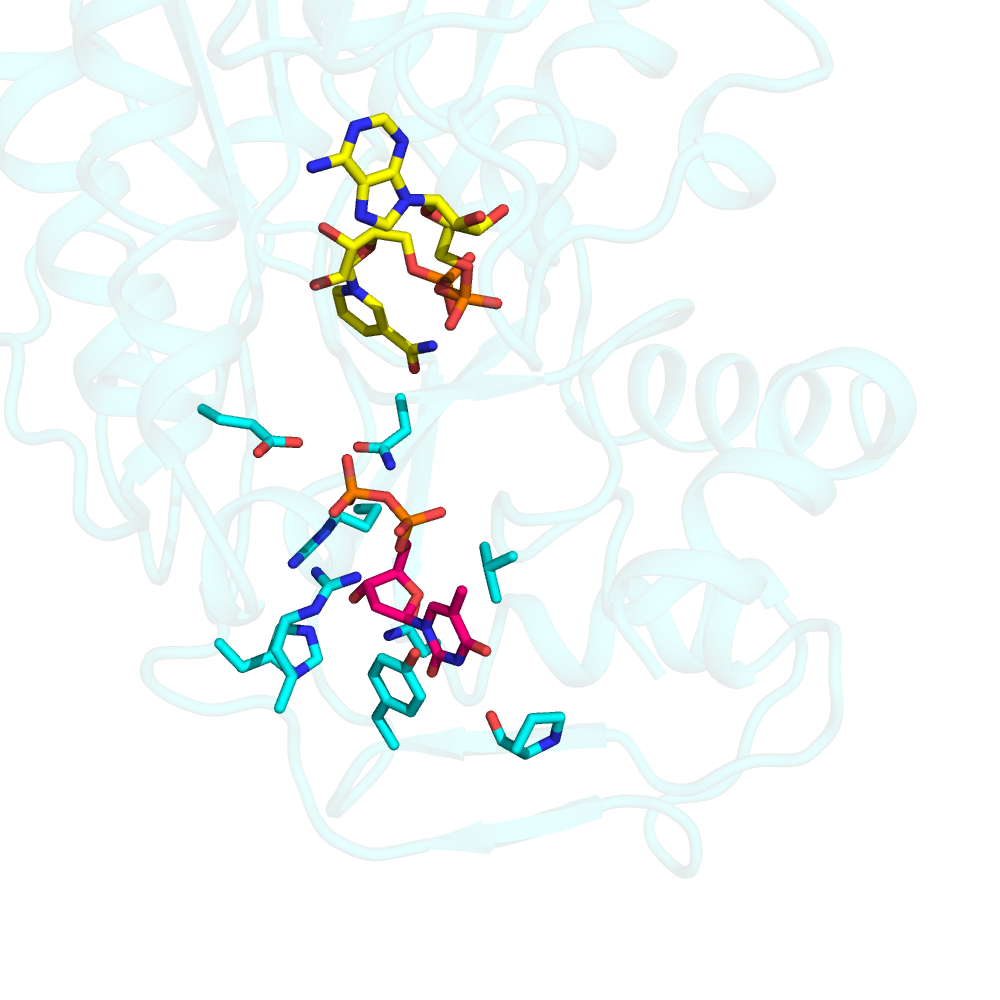


**NAD^+^**

N179

E128

N249

Y207

P205

H276

V190

R214

R273

**TDP**


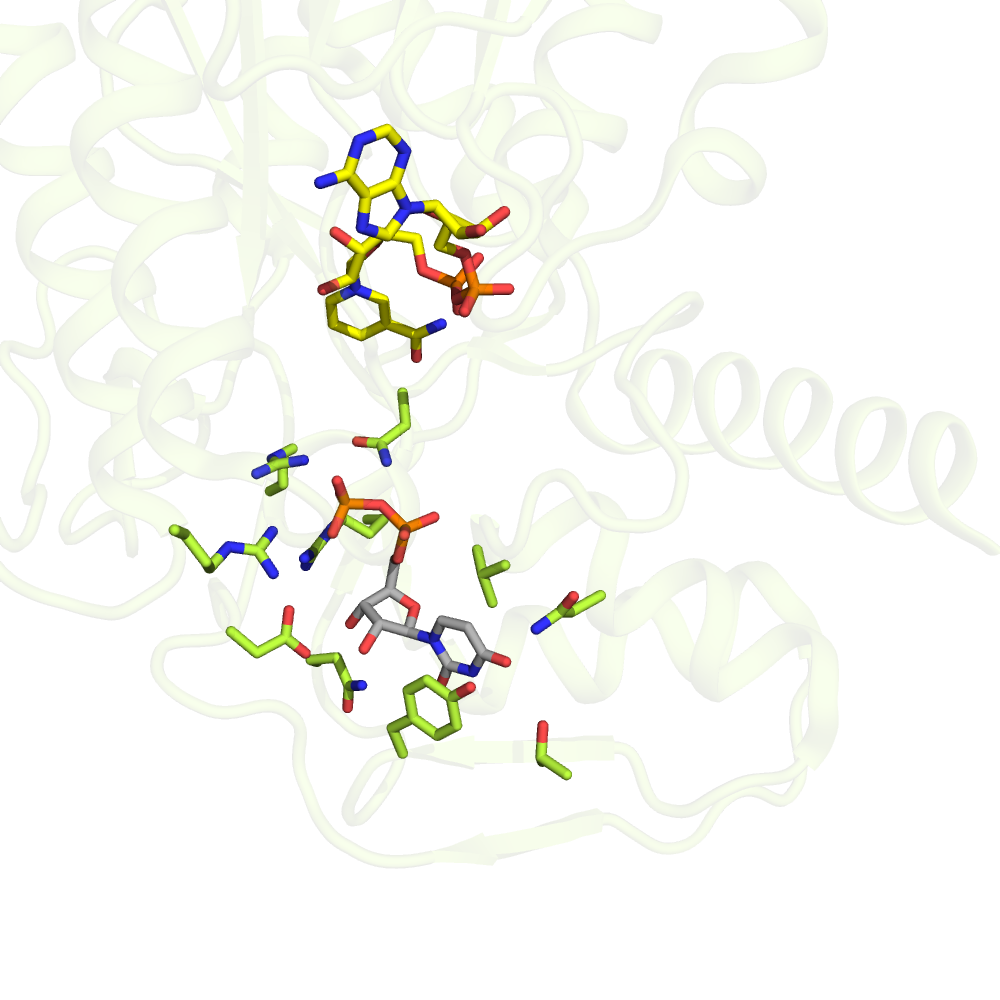


**NAD^+^**

**UDP**

R277

N176

R144

Q211

D273

Y206

T204

N192

V189

R213

**Figure S24: Residues involved in substrate binding.** The residues involved in binding the nucleotide diphosphate substrate fragment are compared. 2B69 is the human protein hUxs1 that produces xylose. NAD+ is shown in yellow, TDP is shown in magenta, and UDP is shown in grey.

**EvdS6**

**2B69**

**Figure S25: Active site loop.** The flexible active site loop is compared between the ligand-free EvdS6, TDP-bound EvdS6, apo 2BLL, and 2B69 structures. The flexile loop is colored in red, with missing electron density in both of the ligand-free structures is shown as a break in the structure. 2BLL is the *E. coli* protein ArnA involved in Lipid A biosynthesis. 2B69 is the human protein hUxs1 that produces xylose. NAD^+^ is shown in yellow, TDP is shown in magenta, and UDP is shown in grey.


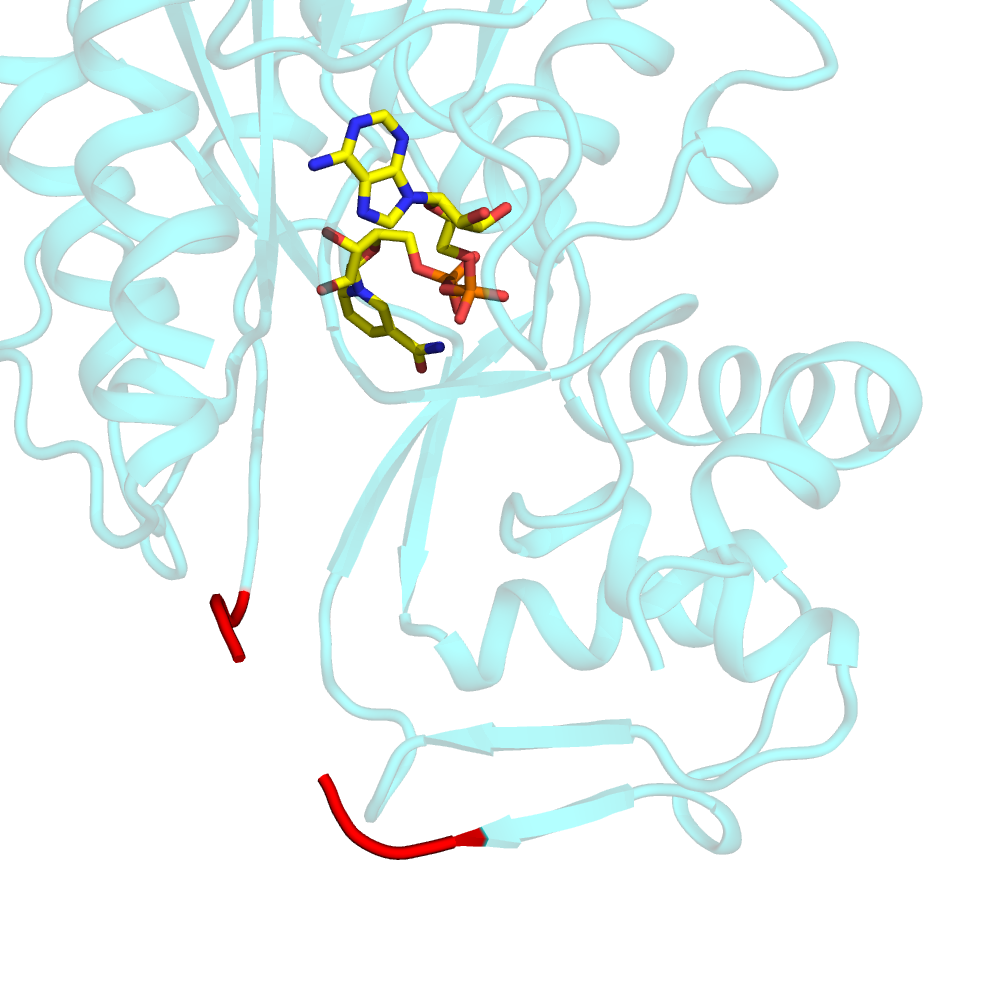


**NAD^+^**

**EvdS6**

**Ligand-Free**


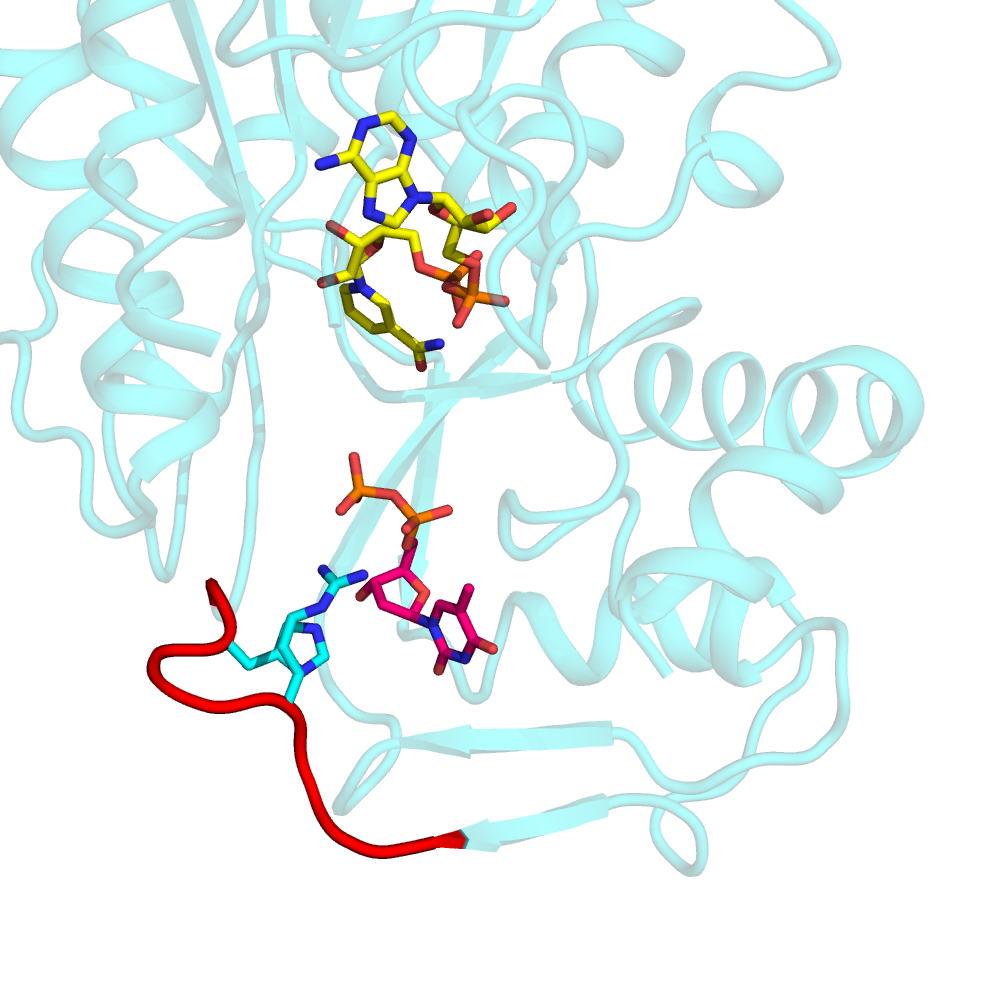


**NAD^+^**

**TDP**

H276

R273

**EvdS6**

**TDP-Bound**


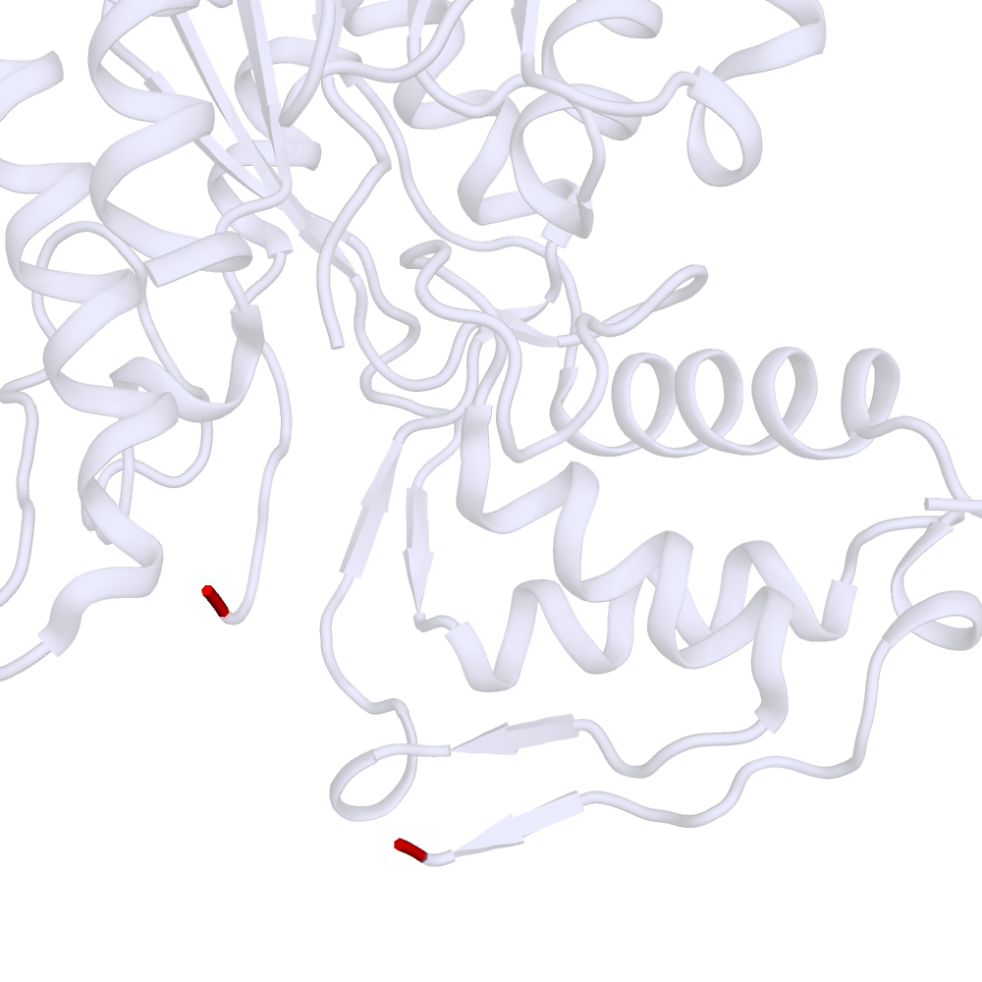


**2BLL**


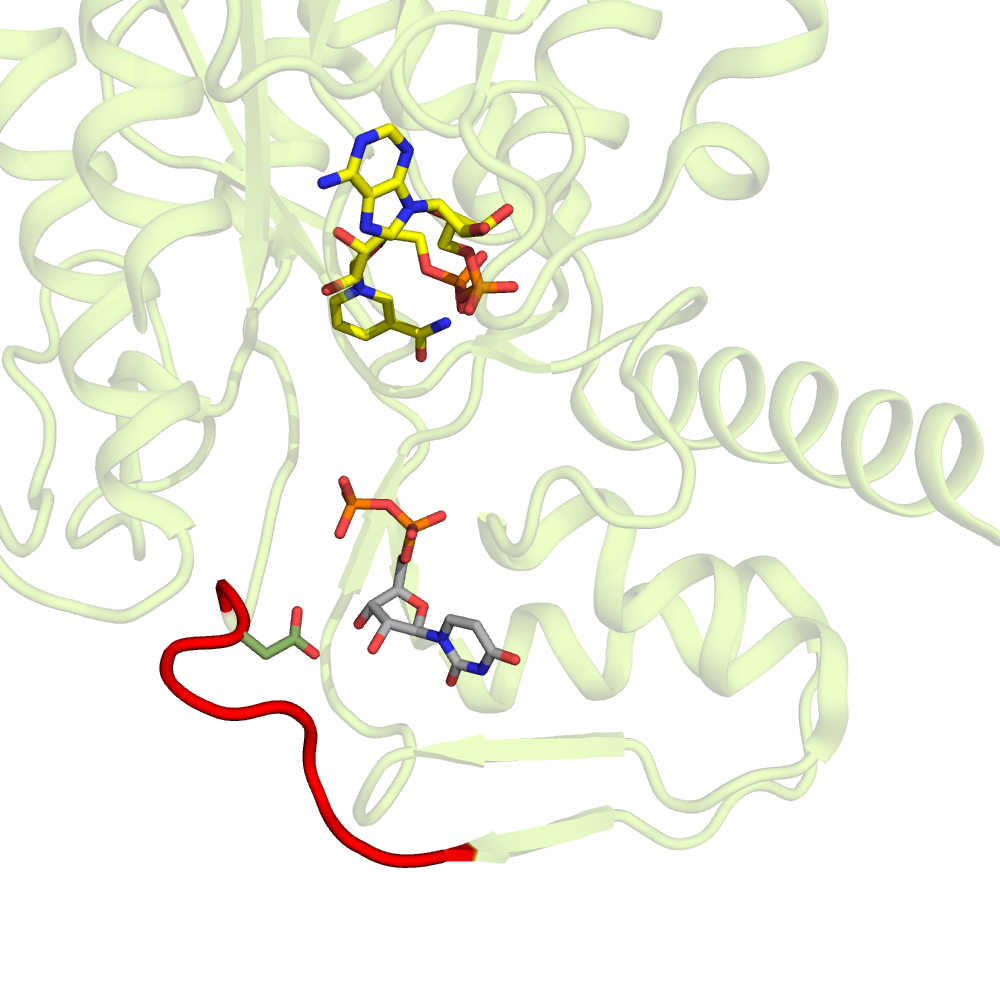


**NAD^+^**

**UDP**

D273

**2B69**

**Figure S26: Electron density of NAD^+^.** The F_o_-F_c_ map is shown as green mesh at σ =1.0 for NAD^+^ in the ligand-free state and the TDP-bound state are compared. There is minimal variation in the orientation of NAD^+^ between the two structures. NAD^+^ is shown in yellow sticks and TDP is shown in magenta sticks. EvdS6 is shown in transparent cartoon representation in cyan.


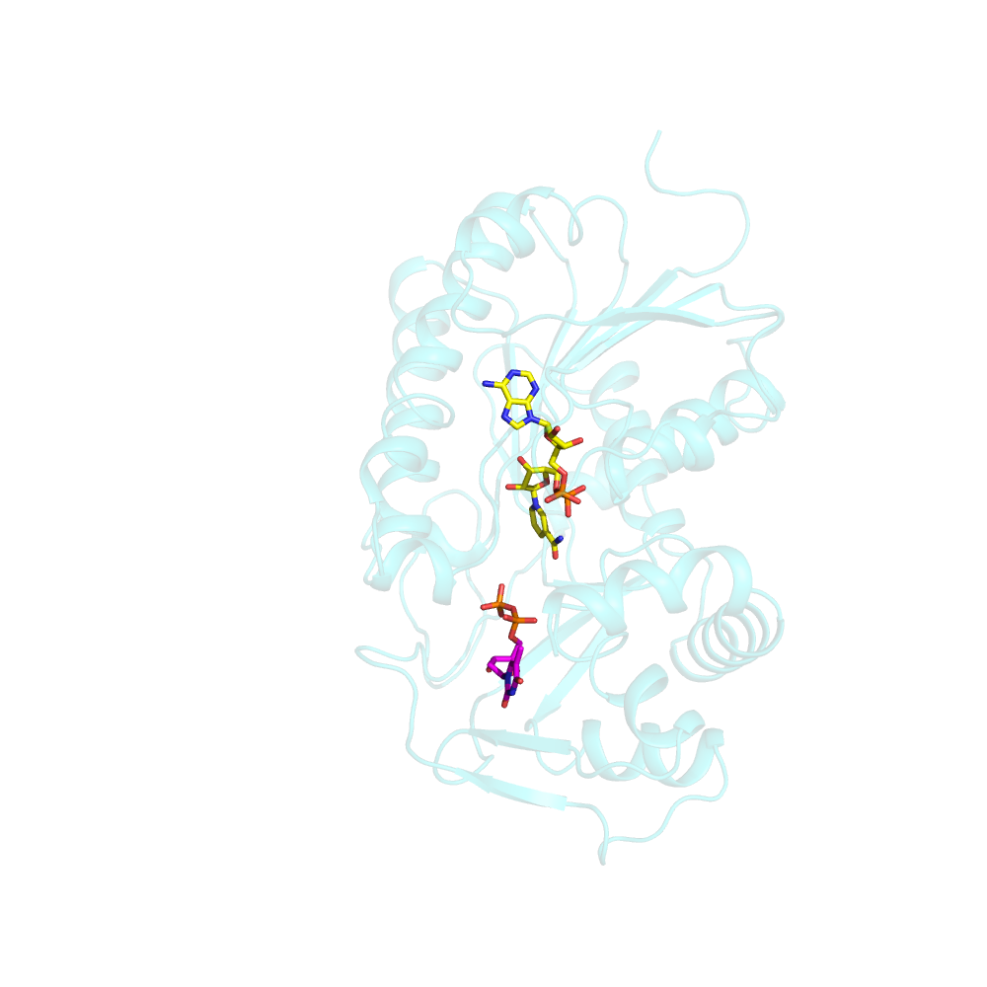

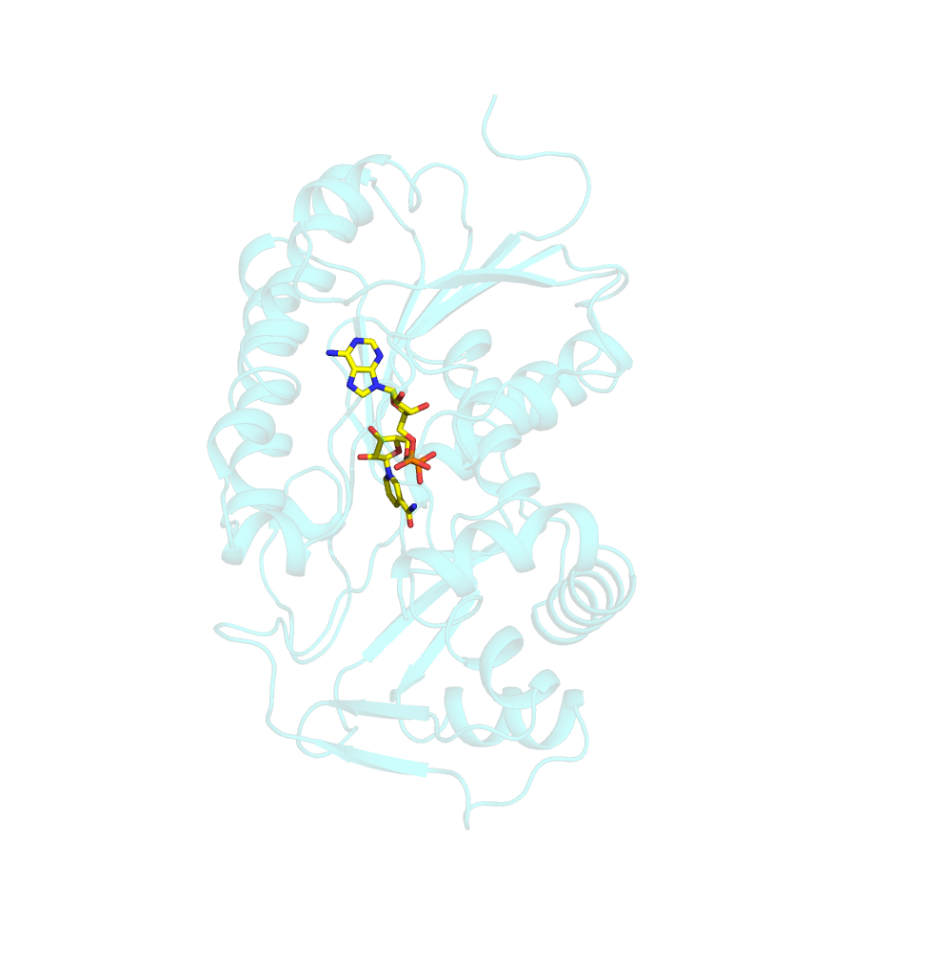

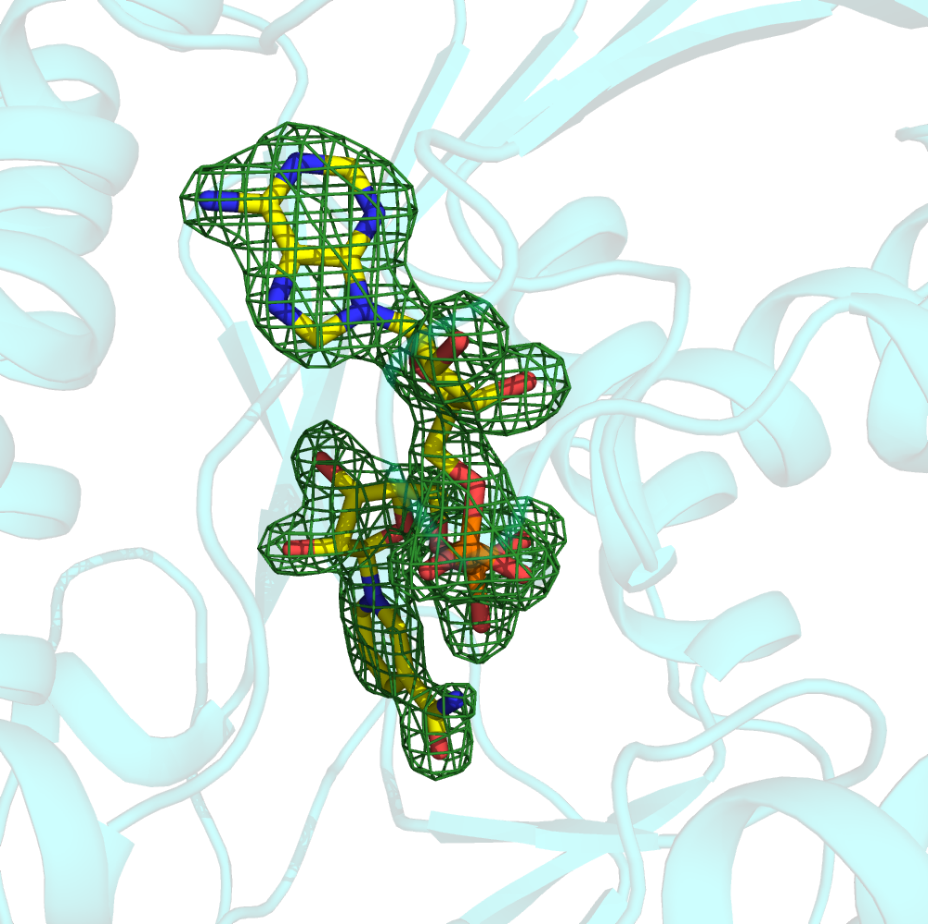

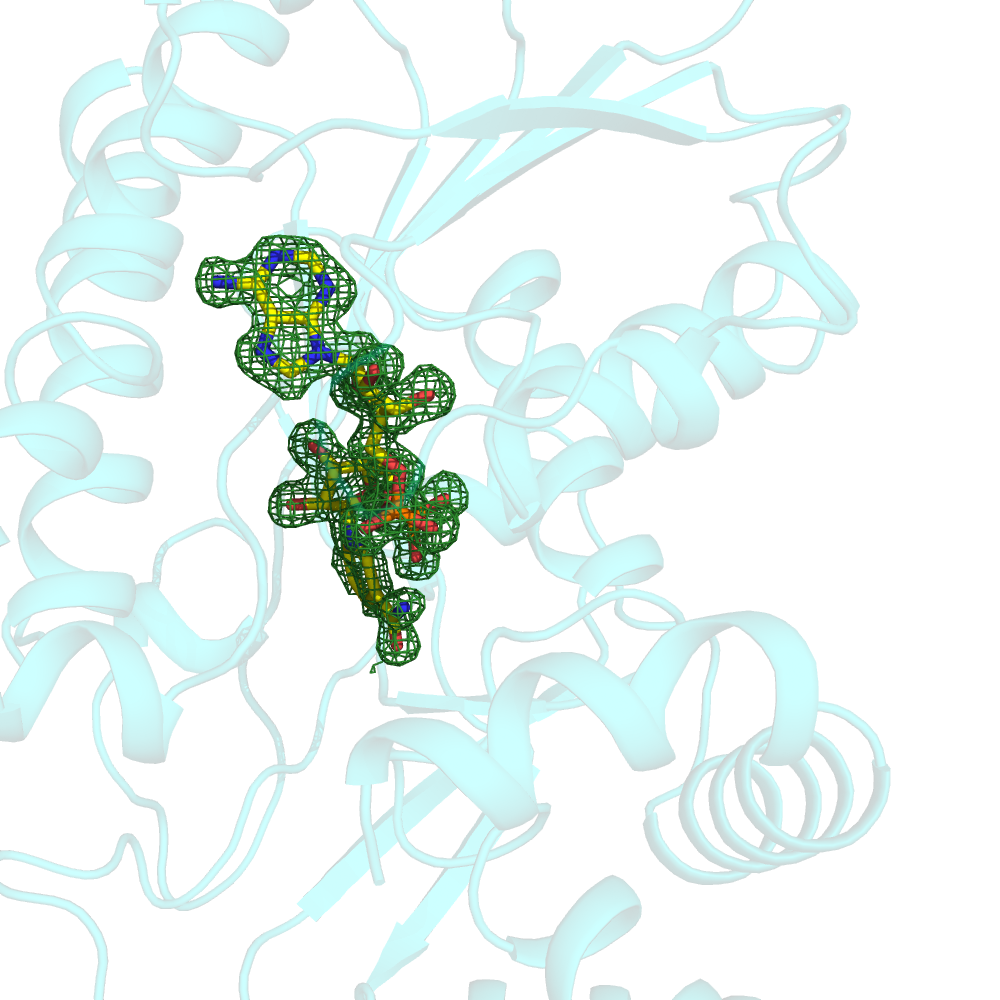


**EvdS6**

**Ligand-Free**

**EvdS6**

**TDP-Bound**


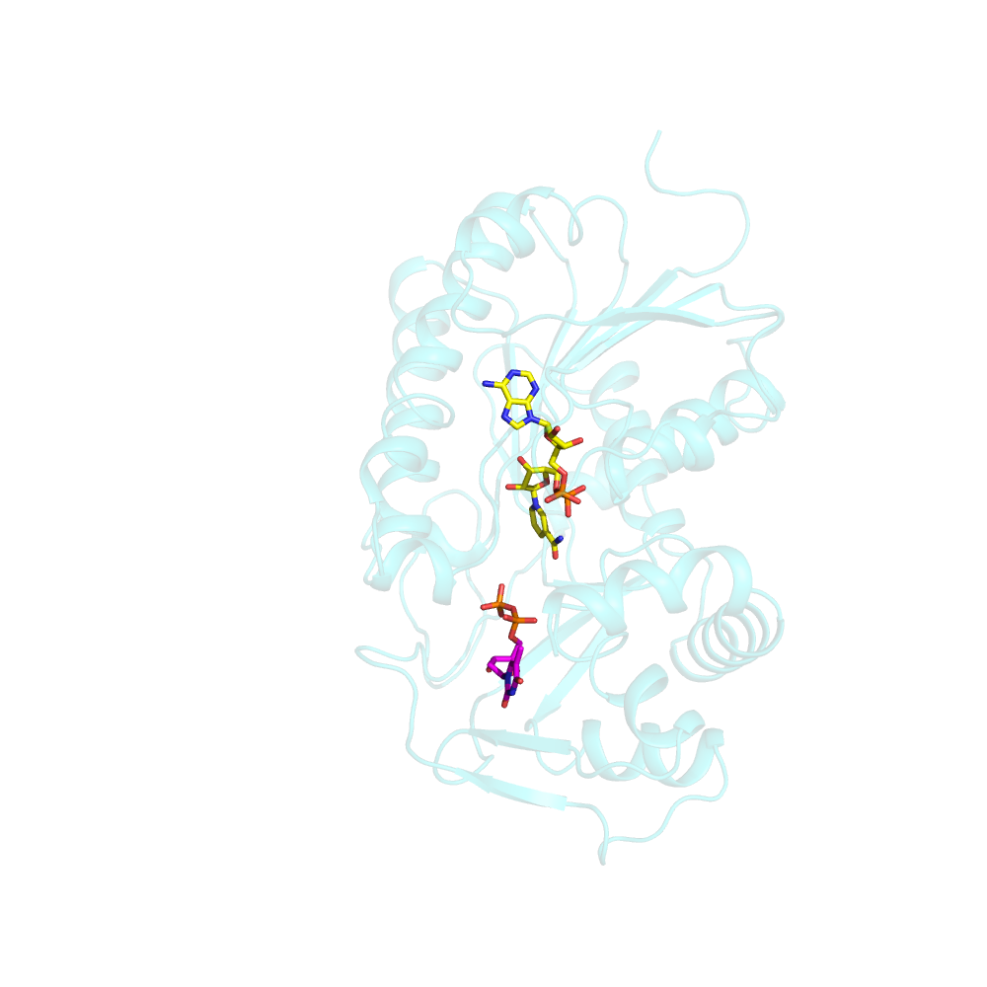

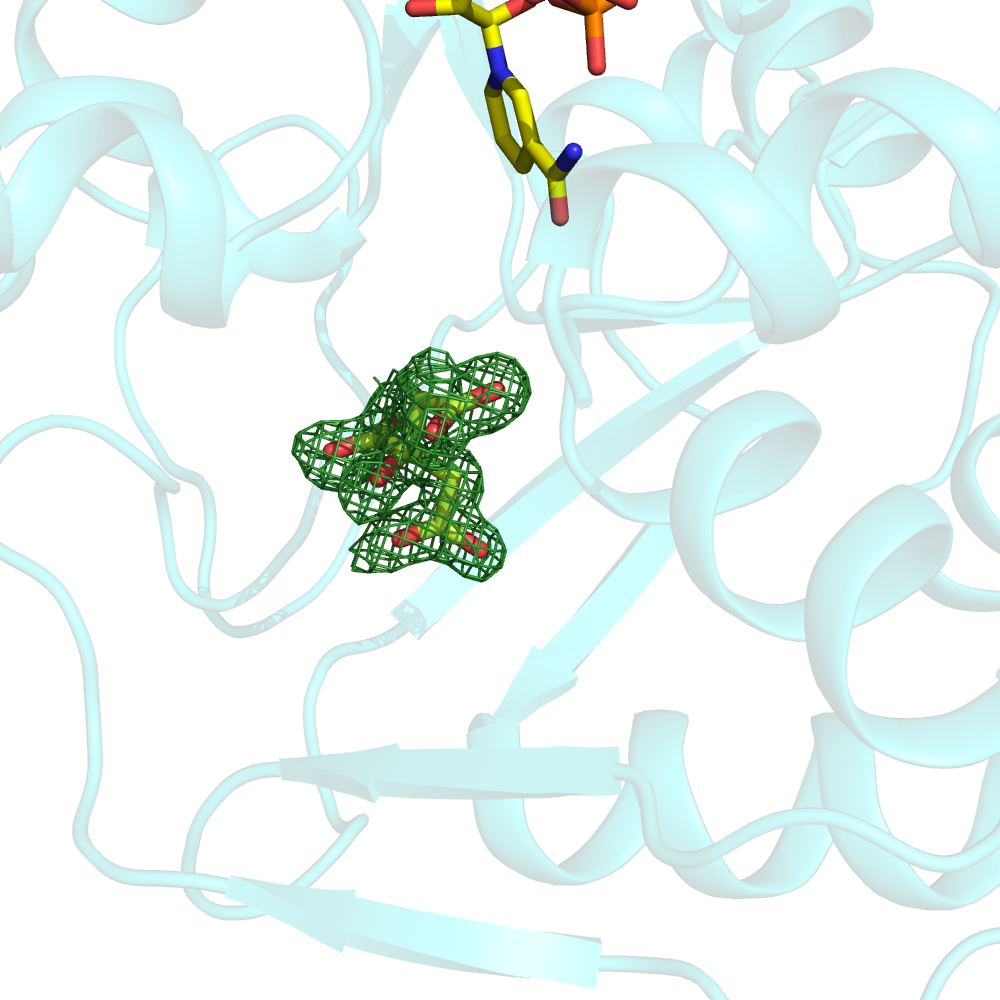

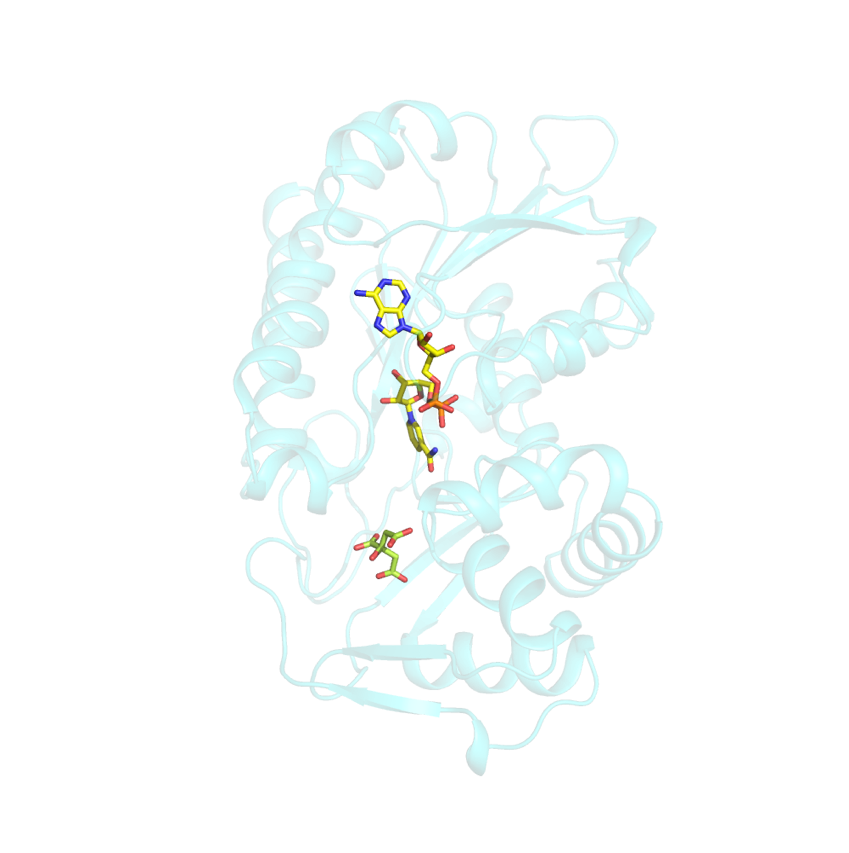

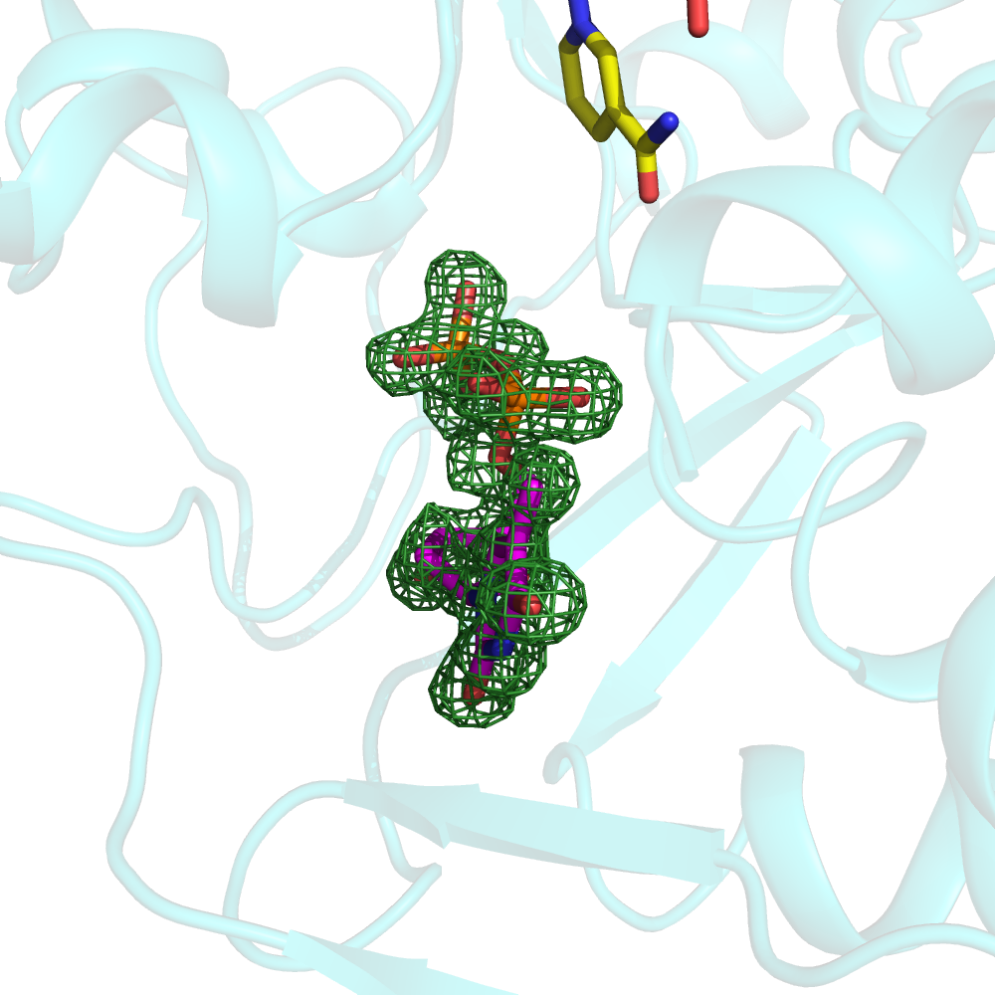


**EvdS6**

**TDP-Bound**

**EvdS6**

**Citrate-Bound**

**Figure S27: Electron density of bound ligands.** The F_o_-F_c_ map at σ =1.0 is shown as green mesh for TDP and citrate is shown. Both ligands bind in the same substrate binding pocket close to the nicotinamide ring of NAD^+^. NAD^+^ is shown in yellow, TDP is shown in magenta sticks, and citrate is shown in green sticks. EvdS6 is shown in transparent cartoon representation in cyan.

**Figure S28: Unresolved electron density at the active site of EvdS6 in the ligand-bound state.** **A.** Unassigned electron density around the active site of EvdS6 monomer in ligand bound state of the protein is shown as 2Fo-2Fc map at 0.4σ in blue mesh. **B.** Unsatisfied electron density is observed even after placing citrate molecule. The most probable reason being the presence of mixed states of the ligands around the active site. These unassigned five/six-member ring like electron densities depict the presence of multiple NDP-like molecules around the active site.


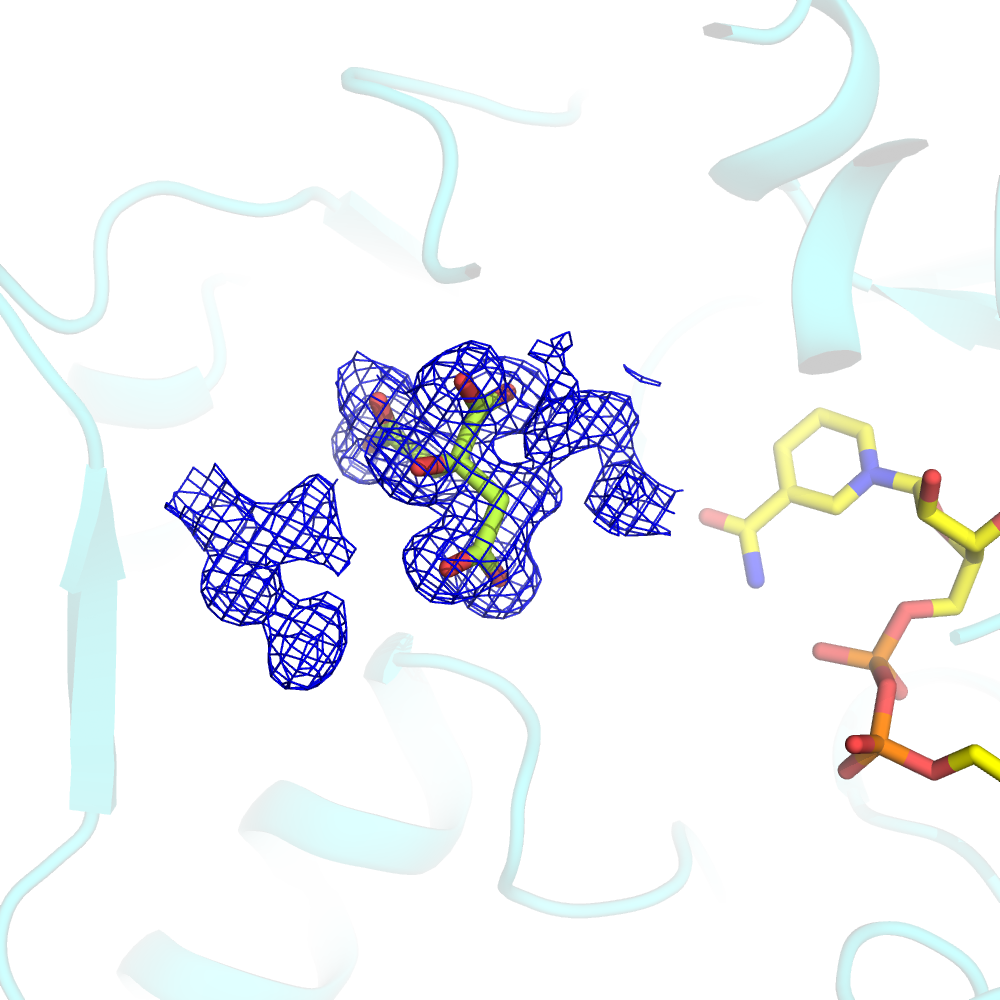

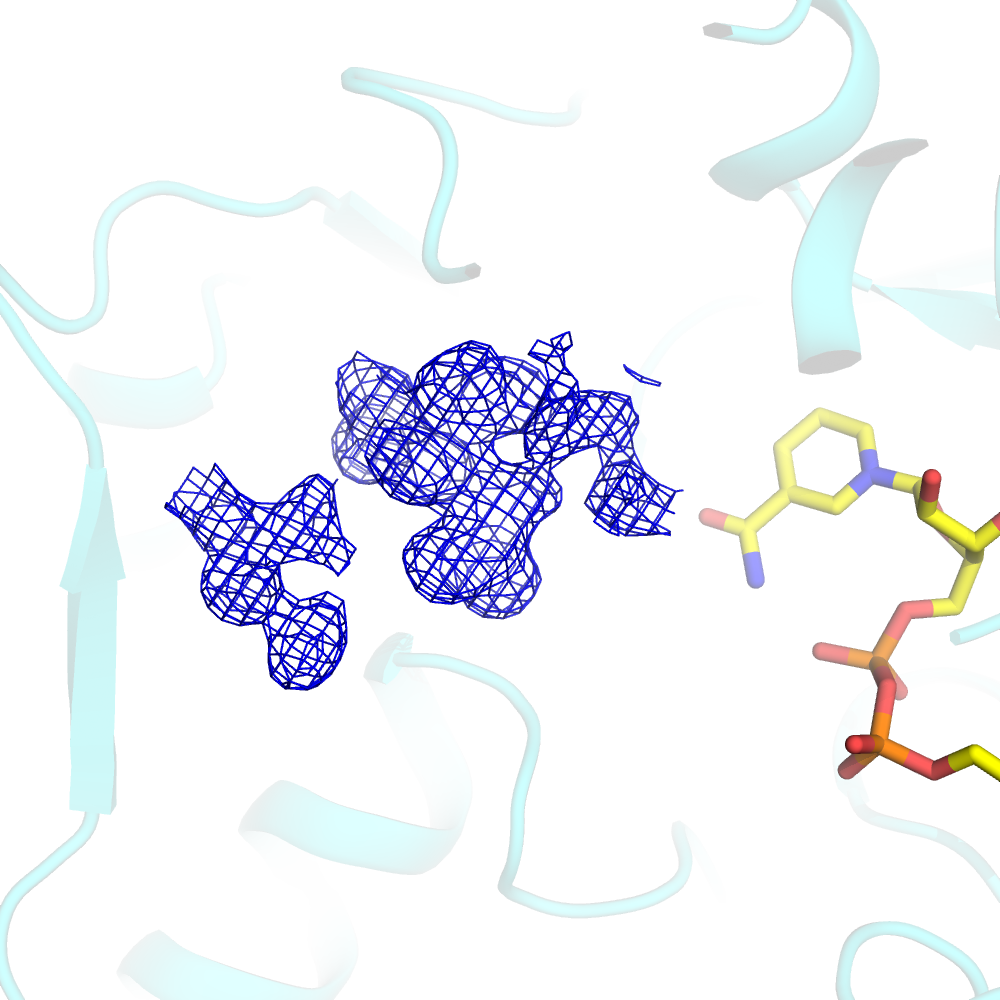


**NAD^+^**

**NAD^+^**

**A**

**B**
